# Recent Advances and Challenges of Nanomaterials-Based Hydrogen Sensors

**DOI:** 10.3390/mi12111429

**Published:** 2021-11-21

**Authors:** Bei Wang, Ling Sun, Martin Schneider-Ramelow, Klaus-Dieter Lang, Ha-Duong Ngo

**Affiliations:** 1 Department of Microsystem Technology, University of Applied Sciences Berlin, 12459 Berlin, Germany; 2 Fraunhofer Institute for Reliability and Microintegration IZM, 13355 Berlin, Germany; Martin.Schneider-Ramelow@izm.fraunhofer.de (M.S.-R.); Klaus-Dieter.Lang@izm.fraunhofer.de (K.-D.L.); 3 Department of Mathematics, Free University Berlin, 14195 Berlin, Germany; Ling.Sun@fu-berlin.de; 4 Center of Microperipheric Technologies, Technical University Berlin, 13355 Berlin, Germany

**Keywords:** hydrogen safety, hydrogen sensor, transition metals, catalytic sensing, micro and nanosensors, metal oxide semiconductors, graphene, graphene oxide, reduced graphene oxide

## Abstract

Safety is a crucial issue in hydrogen energy applications due to the unique properties of hydrogen. Accordingly, a suitable hydrogen sensor for leakage detection must have at least high sensitivity and selectivity, rapid response/recovery, low power consumption and stable functionality, which requires further improvements on the available hydrogen sensors. In recent years, the mature development of nanomaterials engineering technologies, which facilitate the synthesis and modification of various materials, has opened up many possibilities for improving hydrogen sensing performance. Current research of hydrogen detection sensors based on both conservational and innovative materials are introduced in this review. This work mainly focuses on three material categories, i.e., transition metals, metal oxide semiconductors, and graphene and its derivatives. Different hydrogen sensing mechanisms, such as resistive, capacitive, optical and surface acoustic wave-based sensors, are also presented, and their sensing performances and influence based on different nanostructures and material combinations are compared and discussed, respectively. This review is concluded with a brief outlook and future development trends.

## 1. Introduction

Climate change has called people to action to create a more sustainable future. Lower CO_2_ emissions have become a global target in coming decades. Applying renewable clean energies, such as wind, solar, hydropower, biomass, and hydrogen, is the first step towards carbon neutrality in line with the target agreed under the Paris Agreement in the United Nations Framework Convention on Climate Change (UNFCCC) in 2015 [1]. As one of the clean and renewable energies, the combustion of hydrogen (H_2_) emits power, heat, and water, making H_2_ the most promising energy carrier for our future. Moreover, in comparison with electric vehicles, hydrogen fuel cells present an enhanced driving range with the most extended range currently >300 m [2]. The energy transfer and consumption processes avoid CO_2_ emissions. 

Hydrogen is the lightest element in the periodic table. At room temperature, hydrogen shows the form as a gas, dihydrogen, formula H_2_. It is colourless, odourless, tasteless, and combustible. The flammability property of hydrogen is the main concern about its safety. It has a wide range of flammability limits (the mixture of hydrogen and air are approximately from 4% to 77%), very low ignition energy (the ignition energy of the mixture of 23 vol% hydrogen and air is 0.017 mJ, which is only 1/10 of the other gasoline-air mixtures) [3]. The flame propagation speed can even reach a subsonic velocity (975 m/s for a 29.6 vol% H_2_ in air mixture) [3]. Furthermore, hydrogen flame is nearly invisible. Hydrogen combusts very rapidly in oxygen and air and can cause sudden explosions. In hydrogen storage applications, the interaction of hydrogen and the material directly in contact can also cause embrittlement and mechanical degradation in the containment material, which leads to hydrogen spills or leaks [4]. According to the published report of the U.S. National Renewable Energy Laboratory (NREL), for the on-board safety of fuel cells vehicles, hydrogen sensor should have the ability to respond to 1 vol% of H_2_ within 1 s, and to achieve the lowest detection limit of 0.1 vol% of hydrogen operating in the range of temperature from −40 °C to +40 °C and relative humidity within 5% to 95% [5]. The US Department of Energy (DOE) has also published specific technical targets for hydrogen safety sensors, in terms of the measurement range (0.1–10%), operating temperature (−30–80 °C), the response time (less than 1 s), accuracy (5% of full scale), gas environment (ambient air, 10–98% relative humidity range), lifetime (10 years) and interference resistance (e.g., hydrogens) [6,7].

Therefore, future research and developments of very accurate and fast hydrogen sensors will play a crucial role in hydrogen safety. Studies in [3,4] proposed several criteria in consideration of hydrogen sensors, including response time, detection range, signal accuracy, recovery time, low cost and low power consumption, sensitivity to the environment, and so on. Regarding the efficiency of hydrogen leak detection, the gas response was mainly defined as the ratio of the electrical resistance variation in detection gas (R_g_) to that in the air (R_a_):

(1) $$\mathrm{Response}\;\left(\%\right)=\frac{\left|R_{a}-R_{g}\right|}{R_{a}}\times 100\%$$

The sensor response time was defined as the needed time for the sensor to reach 90% signal maximum of hydrogen in the sensor environment. Together with the response time, the recovery time means the time needed to lower the signal maximum of 90%. The lower the concentration of hydrogen that a device can detect, the better it is at preventing a potential explosion.

Detection of hydrogen correlates directly to the interaction of the hydrogen with a selected sensing material. These interaction effects can be catalytic-based, thermal conductivity-based, electrical and electrochemical-based, mechanical-based, optical-based, and even acoustic-based [4]. Among them, transition metals, such as palladium (Pd) and platinum (Pt)-based materials, have been favoured and are still undergoing more in depth research due to their high sensitivity to hydrogen [8,9]. The transition metal Pd in particular has a high-affinity property with hydrogen even at room temperature, making it the most promising hydrogen detection sensor. Another kind of sensing material is the metal oxide semiconductor (MOS), such as SnO_2_, TiO_2_, and ZnO. However, most metal oxide semiconductors exhibit gas sensing performance at elevated temperatures in the range of up to 500 °C [10], due to the energy barriers for electron mobility caused by their bandgaps. Materials like graphene, graphene-oxide, and reduced graphene-oxide have been introduced to gas sensing to enhance the sensing performance because of their mechanical and chemical advantages. Many researchers investigated sensing materials by modifying their forms, sizes, and combinations, developed innovative structures and synthesis methods to improve the sensor response, sensitivity, and selectivity on the one hand, and to reduce the energy consumption and production cost on the other hand. 

This work is divided into three parts. The first part mainly addresses sensing properties of transition metals, like Pd, in different forms in detection applications, including the operating theories, properties, current research, and their performances. The second part focuses on the hydrogen sensors based on metal oxide semiconductors (MOS) involving the bandgaps changes in n-type and p-type MOS during the hydrogen absorption/desorption processes and their performances when applied in hydrogen detection will be discussed. In the third part, we share insights into the applications of graphene and its derivatives-based hydrogen sensors. More interesting research will be explained and compared with performances and reliability.

We hope this work can give our readers an up-to-date overview of promising hydrogen sensors and offer some insights for future work in this area. 

## 2. Palladium-Based Hydrogen Gas Sensors

Palladium has been one of the most promising materials for gas sensing for decades. Due to its high hydrogen affinity and catalytic properties, Pd has been widely used as thin films or nanoparticles for hydrogen leak detection or hydrogen storage. This absorption process leads to lattice/volume expansion and resistance increase during hydrogen diffusion [11,12]. Moreover, Pd’s high state density at the Fermi level makes hydrogen absorption occur even at room temperature reversibly [13].

**Figure 1:**
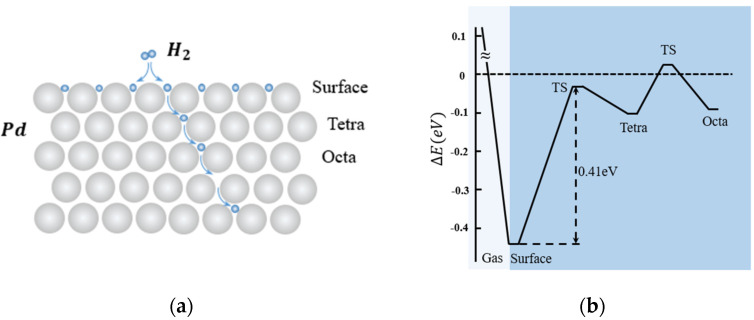
(**a**) Illustration of hydrogen adsorption in Pd (100) [16]; (**b**) diagram of the potential energy profile of hydrogen-absorption [17]. Copyright (2017), with permission from American Chemical Society.

Hydrogen molecules (H_2_) dissociates into hydrogen (H) atoms during hydrogen absorption, and the H atoms diffuse into palladium, occupying the interstitial sites of the Pd lattice as shown in Figure 1a. Most metals require energy input, such as high pressure or elevated temperature, to overcome an active barrier by hydrogen absorption. On Pd surfaces, the dissociative adsorption of H_2_ molecules occurs with little or no activation energy barrier, as shown in Figure 1b [14,15,16,17]. Behind this catalytic activity is a bond alternation mechanism in which the dangling bond of the Pd surface plays an essential role [14,18]. Because of the lower kinetic energy of the surface, the dissociated H atoms are distributed and bonded with the Pd surface rapidly. As the surface reaches its saturation status, the H atoms slowly begin the penetration process into the Pd bulk, during its higher kinetic energy inside the lattices. In Figure 1b, the tetrahedral sites have higher kinetic energy (−0.097 eV) than the sites on the surface (−0.44 eV), and the diffusion to the octahedral sites (−0.092 eV) needs even more energy. The transition state (TS) in Figure 1b can be found by applying the complete linear synchronous transit/quadratic synchronous transit method [19] during the hydrogen diffusion process [17].

Palladium that absorbs dissociated H atoms forms a metal–hydrogen system, which can be labelled as palladium hydride PdH_x_. The absorption can be described in the following forms:

(2) $$H_{2}\to 2H$$

(3) $$\mathrm{Pd}+\mathrm{xH}\;\to {\mathrm{PdH}}_{x}$$

**Figure 2:**
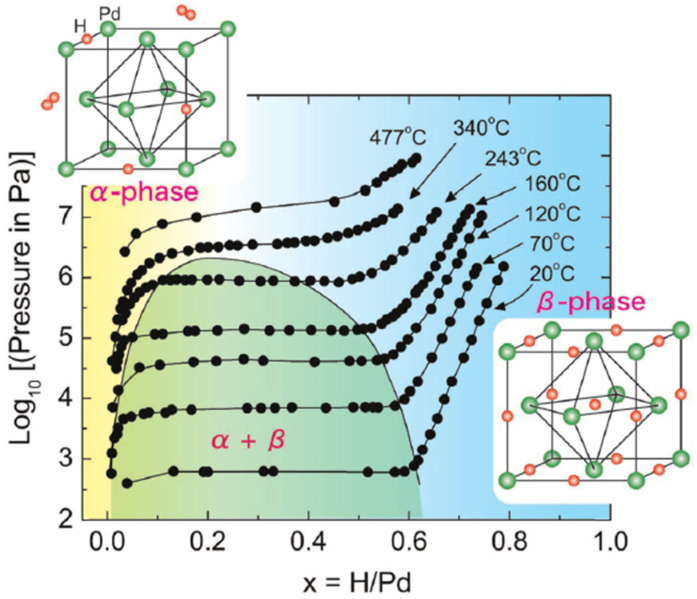
H_2_ pressure–composition–temperature (P–C–T) phase diagram of the Pd-H system [22,23]. Copyright (2019), with permission from Chemistry Europe.

Here x is hydrogen content. In many works, the hydrogen adsorption process is modelled as a kinetic process [11,12,13,14,20,21,22,23]. During this process, different phases can appear, coexist and transform from one to another at variant pressure P, temperature T, where the hydrogen composition x, i.e., H/Pd, changes consequently. Figure 2 shows such a pressure–composition–temperature (P–C–T) diagram [24,25]. When x is small, Pd and H atoms form an unsaturated solid solution, and the state of the Pd-H_x_ system reaches α-phase like in Figure 2 (PdH_0.015_ lattice constant = 0.3894 nm@298 K). Increasing the content of hydrogen x up to about the x ≈ 0.7, the PdH_x_ presents a saturation status β-phase (PdH_0.607_: lattice constant = 0.4025@298 K) solid solution, which leads to the lattice expanded [20,21]. When x is in between, the Pd-H system state is in the region of the coexisting region. This region is unstable and nd it is not clear where the system will go at the next moment, either converging towards the α-phase or evolving towards the β-phase at the next moment. As shown in Figure 2, the coexisting region of two phases (α- and β-phase) covers a wide H_x_ composition range, from about 0.01 to 0.62, while the kinetic energy in the environment (mainly, the pressure and the temperature) varies [24,26]. In the saturated state, the octahedral site of the Pd is fully filled with hydrogen atoms. This saturation can introduce about 10.9% volume expansion of the Pd material; thus, it is undesirable for the material to reach the β-phase. In a worst-case scenario, for instance, for the Pd films, the tensile stress consequently leads to the failure of sensing devices [27,28]. Additionally, previous works [28,29,30] have observed that some hysteretic behaviour in the resistance caused irreversible structural changes in Pd bulk and Pd films, which can lead to sensing stability problems.

**Figure 3:**
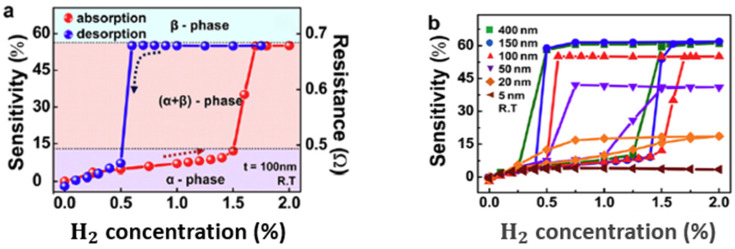
Hysteresis curve of sensitivity during the process of absorption and desorption of hydrogen from 0 to 2% concentration (**a**) for the Pd thin film with the thickness of 100 nm and (**b**) for the Pd thin film with thicknesses ranging from 5 to 400 nm [26]. RT means room temperature. Copyright (2010), with permission from Elsevier.

Available work went beyond the surface of the Pd material to determine how the thickness affects sensing performance. In [26], Lee et al. have examined Pd thin films’ sensitivity of variant thickness, from 5 nm to 400 nm. The results in Figure 3a show a sensitivity curve during the process of hydrogen absorption (red curve, from the bottom going towards higher H_2_ concentration until there is a quick increase of sensitivity), and the process of hydrogen desorption (blue curve, from the most upper right side, going towards lower H_2_ concentration until there is a sharp decrease of the sensitivity in the end). The cycle formed around the area of α- and β-phases is also called hysteretic behaviour. During the interacting process of hydrogen and Pd, free electrons from hydrogen can move through and thus affect the electrical resistance within the thin film. Let t be the thickness of a Pd film. As Figure 3b shows, by 5 nm thickness, the process of hydrogen absorption and desorption forms a relatively flat curve at a quite low sensitivity level. Each curve with *t* between 20 nm and 400 nm shows a hysteretic behaviour covering a lower and an upper sensitivity range, with H_2_ concentration ranging from 0.25% to 1.75%. Observation of these curves indicates that the range of sensitivity that can be covered increases when t increases from 20 nm to 150 nm. The highest sensitivity level that a Pd thin film can reach also increases when the thickness of the film increases. These curves reach the highest sensitivity value at 150 nm, where a larger t does not result in a better sensitivity level. This saturation of sensitivity can be attributed to the limited depth to which the hydrogen atoms can penetrate Pd material. This limited penetration depth also means that the phase transition occurs mainly near the surface region of the Pd thin film. However, the low sensitivity level of the 5 nm-thick films indicates that the too-small thickness can restrain the capacity of the absorption of hydrogen atoms and the Pd lattice expansion.

The response time of a hydrogen sensor is employed to describe the penetration/diffusion process of the dissociated hydrogen atoms from the surface into the sensing materials. Due to the penetration process of hydrogen atoms, hydrogen sensors based on Pd thin-film suffer from slow response and poor stability for sensing performance [31]. Instead of pure Pd, much of the literature studied palladium alloy and bilayers like Pd/Ag [32], Pd/Ni [33], Pd/Au [34], Pd/Mg [35] and Pd/ZnO [36] for faster responses or better durability. 

The hydrogen diffusion rate correlates with the temperature significantly. This relation [37] is defined by diffusion coefficient *D* as:

(4) $$D=D_{0}\exp\left(-\frac{Q}{RT}\right)$$

where $D_{0}$ is the diffusion constant, *Q* is the diffusion activation energy, *R* is the ideal gas constant and T the temperature.

With Fick’s 2nd law for the case of three-dimensional diffusion, the process can be written as:

(5) $$\frac{\partial \rho }{\partial t}=\nabla \cdot \left(D\nabla \rho \right)$$

where *ρ* is the density of hydrogen atoms.

**Figure 4:**
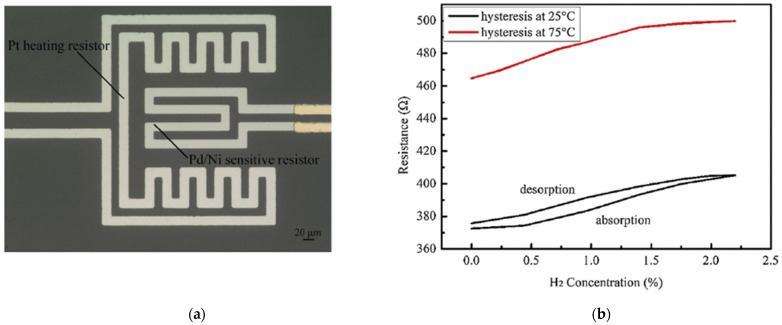
(**a**) The optical microscope image of the Pd/Ni sensor and (**b**) the hysteresis of hydrogen absorption and desorption at 25 °C and 75 °C [37]. Copyright (2019), with permission from Elsevier.

Liu et al. in [37] investigated the temperature dependence of Pd-based thin film, and developed a hydrogen sensor based on sputtered Pd/Ni alloy thin film with a Pt heating resistor as shown in Figure 4a. This sensor had significantly improved response and recovery time at 75 °C in comparison with the sensor based on the exact sensing mechanism [38]. Furthermore, Liu discussed the effect of different temperatures on the Pd/Ni film. At room temperature, the oxygen atoms of the environment at the grain boundary increased the resistance of the alloy film without hydrogen. When the hydrogen atoms diffused inside the film, they reacted with oxygen to form water, which removed oxygen from the film surface and caused a resistance decrease of the Pd/Ni film. The oxygen atoms were driven to leave the grain boundary by raising the working temperature, and more hydrogen atoms could permeate deeper into the film. This process built additional electrical pathways by filling hydrogen atoms in more lattice vacancies, thus increasing electrical conductivity and decreasing resistance in a reverse way. As the temperature rises to a specific value, like 100 °C, some absorbed hydrogen atoms on the surface are oxidized, which weakens the penetration process and leads to the sinking of the sensing response (1.85% decrease at 100 °C).

Interestingly, at 75 °C, the Pd/Ni film was reported with optimized sensing properties, such as better linearity than at 25 °C. Additionally, at a higher operating temperature, the hydrogen absorption and desorption processes rarely present hysteresis, as shown in Figure 4b, because the heating process accelerated diffusion and release of hydrogen atoms in absorption and desorption processes. On the other hand, the high operation temperature also has disadvantages. It can cause high power consumption and increase the noise signal, further limiting the performance and stability of applying Pd-film-based sensors. 

By contrast with the use of the electrical resistive principle, some researchers investigated sensors by applying the surface acoustic wave (SAW) [33,39] and optical reflection principles [40,41] to improve the sensor performance at room temperature. Wang et al. in [33] developed a Pd–Ni alloy thin film-coated SAW hydrogen detection sensor with high stability. In their study, the Pd–Ni thin-film was deposited by applying an RF magnetron co-sputtering from separate Pd and Ni targets onto a SAW propagation path for sensing, and the response time of the 40 nm Pd–Ni alloy thin-film can approach less than 10 s at room temperature. Moreover, their experiments also indicated a trade-off effect between film thickness and diffusion velocity of hydrogen atoms. As the thickness of the film increased, the diffusion of dissociated hydrogen slowed down with higher sensitivity. Another highlight of the study is the long-term stability. The sensor has been reported to have a half-year detection error of about 3.7%, which can be explained by the fact that Ni doping in Pd helps to suppress the lattice destruction in gas adsorption. In comparison to SAW sensing mechanism, sensors based on sputter-deposited Pd–Y alloy film in [40] use reflected optical signal intensity in hydrogen detection. As Song et al. reported [40], the sensor has a fast response/recovery time and sensitive signal value in hydrogen concentration ranging from 0.1% to 2% after the annealing process.

As a summary in this part, the Pd thin-film based or Pd alloy-based thin film-based hydrogen sensors could have high sensitivity due to their large contact surface. However, the thickness increase of the thin film slows down the hydrogen diffusion velocity, thus weakening the sensor performance, especially for the sensors using an electrical sensing mechanism. Another disadvantage is of the Pd thin film is the lattice expansion. For high H_2_ concentrations (more than 2%), the Pd lattice expansion leads to the Pd film buckling or collapsing, making the sensor unstable, irreversible, and unreliable. In order to improve the sensor performance accordingly, an external heater can be employed to accelerate the diffusion process. Moreover, the SAW-based and optical sensing mechanism-based sensor can also be used to enhance the sensing response of the sensors.

To better meet the requirements of high sensitivity, rapid response, and reliable performance, another small form of Pd has been investigated for decades by many researchers [42,43,44,45,46,47,48], the Pd nanoparticles. Due to its unique physical and chemical properties, drastic changes can be observed during the hydriding process using Pd nanoparticles, such as the H_2_ pressure-composition phase diagram [42,43,44], hysteresis [42,49,50], vibrational density of states [45,46], structures [47,48], and so on. 

**Figure 5:**
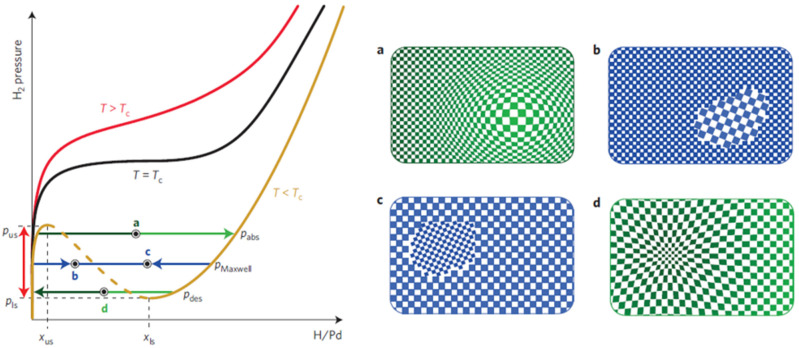
Schematic pressure-composition isotherms at temperatures above, equal and below the critical temperature Tc. The upper spinodal pressure $P_{us}$ and the lower spinodal pressure $P_{ls}$, together with the corresponding concentration $X_{us}$ and $X_{ls}$, are indicated for the gold isotherm with $T>T_{C}$. The full-spinodal hysteresis is indicated with the red arrow. The blue line corresponds to the incoherent pressure changes obtained using the Maxwell construction [55]. In bulk Pd H absorption occurs at $P_{abs}\approx P_{\mathrm{Maxwell}}$ and an expanded β-PdH_*x*_ phase nucleates incoherently in the dilute α-PdH_*x*_ phase (**b**), whereas during desorption at $P_{des}\approx P_{\mathrm{Maxwell}}$ the dilute α-PdH_*x*_ nucleates and grows in the β-PdH_*x*_ phase (**c**). In the case where coherent absorption occurs at $P_{abs}\approx P_{\mathrm{us}}$. (**a**) there is no coexistence of the α- and β-PdH_*x*_ phases [56,57]. The gradient in H concentrations leads to continuous spatial variations of the lattice spacing. The same occurs during coherent desorption at $P_{des}\approx P_{\mathrm{ls}}$. (**d**). According to Schwarz and Khachaturyan [56,57], the hysteresis is comparable to full-spinodal hysteresis. The black line corresponds to the critical isotherm at $T=T_{c}$ and the red line to a supercritical isotherm at $T>T_{c}$. [50]. Copyright (2016), with permission from Nature Publisher Group.

It is well known that significant lattice distortions accompany the hydrogen absorption and desorption [51,52], which essentially affects the reliability and performance of devices in applications like hydrogen sensing, and hydrogen storage. Due to their thermodynamic dependence on sizes [53], palladium nanoparticles have a coherent phase transformation process during the hydrogen absorption and desorption below the critical temperature Tc [50,54]. In that transformation, the variation in hydrogen concentration leads to a modulation of the host lattice accompanying coherency stress and elastic energy contributions to the enthalpy, as shown in Figure 5a,d. The pressure-composition isotherms of coherent transformation show a significant pressure difference between absorption and desorption processes, forming a large hysteresis phenomenon. 

By contrast, the absorption and desorption in bulk palladium or larger particles are consistent with incoherent phase transformation. The dilute α-phase and the concentrated β-phase coexist at thermodynamic equilibrium with the dislocation of lattices, which leads to minimizing elastic stress and more negligible hysteresis, as illustrated in Figure 5b,c. 

Several Pd-based hydrogen sensors applying micro- and nano-sensing structures have been investigated in the literature, such as Pd/Y [32], Pd/ZnO [39], Pd [58,59], Pd/TiO_2_ [60], Pd/Ni [61] and Pd/SnSe [61]. As synthesis methods, the direct current (DC)- or radio frequency (RF)-sputtering methods have been used to fabricate thin-film structures for both electrical resistive [58,60,61] and SAW sensors [33]. Sensing structures based on nanowires and nanoparticles were synthesized mainly with deposition methods [39,61,62,63,64,65,66,67,68,69,70,71,72,73,74,75,76,77,78,79,80,81,82].

**Figure 6:**
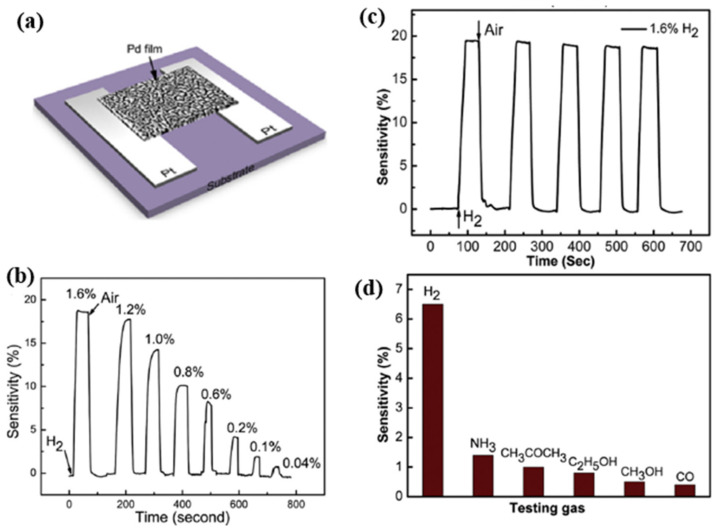
The hydrogen response of nanoporous Pd film sensors at room temperature. (**a**) The transient response to various concentrations of H_2_ in the air; (**b**) the H2 concentration-dependent response time and sensitivity; (**c**) repeatability; (**d**) selectivity [58]. Copyright (2016), with permission from Elsevier.

Hu et al. in [58] developed an innovative processing method to improve sensing properties through a new nanoporous-structure of Pd thin film for H_2_ sensing applications with a specific surface-volume ratio. In their work, a nanoporous structural Pd thin film was achieved through DC sputtering and followed by annealed process under vacuum conditions. Specifically, the porous structure was formed by the competition between the melting of ultrathin Pd film and the surface tension effect during the annealing process. With the optimized annealing temperature of 300 °C, the Pd film exhibited significant sensing properties, with a response time of ~25 s, and a recovery time of about 10 s due to the sizeable reactive surface of the porous structure. The sensor had a wide concentration range of 0.04–1.6%. Additionally, as Figure 6 shows, the sensor exhibits good repeatability, and its sensitivity towards hydrogen was about 6–10 times higher than other reductive gases, like CO, NH_3_, CH_3_OH., at room temperature. 

**Figure 7:**
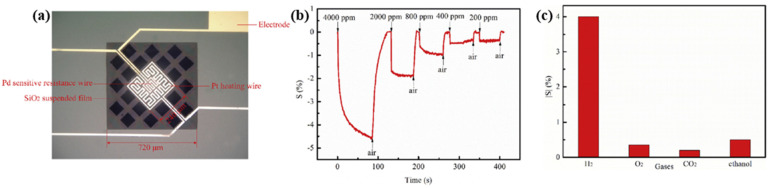
(**a**) Microscope image of a hydrogen sensor sample. (**b**) Responses of different H_2_ concentrations at 400 °C, and (**c**) responses of the sensor to various gases (4000 ppm) at 400 °C [59]. Copyright (2019), with permission from Elsevier.

Liu et al. integrated a Pt micro coplanar hotplate beside a suspended Pd-film sensing layer to control the sensor’s temperature [59]. At low temperatures, e.g., 50 °C, with a low power consumption of about 7.5 mW, the suspended sensor had a response time of about 30 s, and the recovery time was 17 s for 4000 ppm H_2_ concentration and sensitivity of 0.2%. As the power consumption of the Pt micro heater was increased to 65 mW, the working temperature rose to 400 °C, accordingly. This results in a significantly improved sensor response, with a more rapid response/recovery time of 10 s /15 s, a higher sensitivity of 4%, and better selectivity towards hydrogen at the same H_2_ concentration. As mentioned by Liu et al., the reaction mechanism of Pd films at high temperatures is different from that at room temperature. Firstly, Pd reacts with oxygen to form PdO on the surface rather than absorbing oxygen. Once the hydrogen gas is released in the test environment, Pd is reduced from PdO by reaction with H_2_. Secondly, due to the presence of PdO, the reduction reaction between hydrogen and PdO leads to a significant drop in sensor resistance, and the reaction of PdO with hydrogen produces a negative sensitivity signal, as shown in Figure 7.

Mao et al. [60] investigated a hydrogen gas sensor based on nanoporous palladium and titanium dioxide (TiO_2_) composite film. At the interface of the Pd and TiO_2_ layers, both Pd hydride and migrated H atoms react with the adsorbed oxygen on the TiO_2_ surface while injecting electrons into the TiO_2_. The reactions between migrated H atoms and the adsorbed oxygen can be described with Equations (6) and (7):

(6) $${\mathrm{PdH}}_{x}+\frac{x}{2}O^{-}\left(\mathrm{ads}\right)\to \mathrm{Pd}+\frac{x}{2}H_{2}O+\frac{x}{2}e^{-}$$

(7) $$O^{-}\left(\mathrm{ads}\right)+2H\;\to {\;H}_{2}O+{\;e}^{-}$$

The Pd hydride process leads to an increase in resistance on the one hand, while the reaction mechanism of TiO_2_ leads to a decrease in resistance on the other. Therefore, tuning the thickness of the TiO_2_ layer can adjust the sensing performance. As reported by Mao et al. in [60], the best performance was achieved with a 10 nm thickness of TiO_2_. At 0.4% H_2_ concentration, the recovery time of the gas sensor was 8 s, which was faster than 13 s at 0.8% H_2_ concentration. 

**Figure 8:**
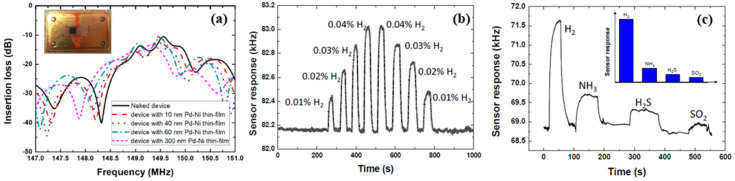
(**a**) Measured frequency responses of the proposed sensing device depending on the Pd–Ni thin-film thickness, inset: picture of the prepared sensing device; (**b**) measured sensor responses relating to the 40 nm Pd–Ni alloy thin-film coated sensing device to various hydrogen gas concentrations; (**c**) selectivity testing of the proposed Pd–Ni alloy thin-film coated sensing device [39]. Copyright (2017), with permission from MDPI.

Wang et al. in [33] investigated surface acoustic wave (SAW) devices based on Pd–Ni alloy thin films due to their fast response, low cost, satisfactory stability and remarkable sensitivity when used for hydrogen sensing. As reported in [33], the operation frequency of the devices decreases with the increasing thickness of the Pd–Ni film due to the mass loading effect. Furthermore, as shown in Figure 8, the enhanced acoustoelectric coupling effect in the thicker films raises the wave attenuation as the thickness of the Pd–Ni film increases. Increasing the thickness of the Pd–Ni film can slow down the diffusion velocity, leading to a slower response, but it can also adsorb more gas molecules, and therefore a higher sensitivity can be expected. The highest sensor response observed with a 300 nm thick Pd–Ni film-coated device was about 8.7 kHz. The fastest response was obtained with a 40 nm film with a relatively fast sensor response (2.75 kHz to 0.1% hydrogen) of ~7 s. As shown in Figure 8, the SAW-based gas sensor possesses significant working stability with a detection error of ~3.7% for six months and selectivity of 0.1%. Viespe et al. developed a SAW gas sensor by employing a Pd/ZnO bilayer for hydrogen sensing applications at room temperature [39]. The sensitive layers were deposited onto ST-X quartz substrates by pulsed laser deposition (PLD). The high laser deposition pressures enabled the sensing film to have a large porosity and a big surface area. As a result, the sensor has a fast response of 16 s at room temperature and a detection range from 2000 ppm to 20,000 ppm. 

**Figure 9:**
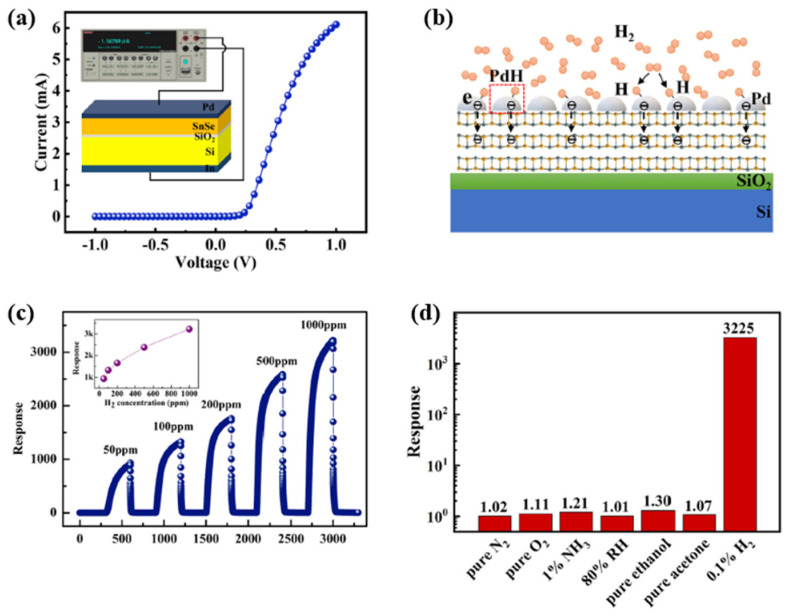
(**a**) The I–V curve of a SnSe/SiO_2_/Si heterojunction. The inset shows a schematic illustration of the measurements. (**b**) Schematic illustration of the sensing behaviour of the Pd-decorated SnSe/SiO_2_/Si heterojunction towards H_2_. (**c**) The consecutive dynamic response of the heterojunction upon exposure to the above concentrations of H_2_. The inset shows the dependence of the response on the H_2_ concentration. (**d**) The sensor’s response to pure N_2_, O_2_, ethanol and acetone, 10,000 ppm NH_3_, 80% relative humidity (RH) (H_2_O) and 1000 ppm H_2_ [61]. Copyright (2021), with permission from Elsevier.

Xu et al. investigated hydrogen gas sensors with two-dimensional (2D) layered tin monoselenide (SnSe) ultrathin films deposited on SiO_2_/Si substrate, which were decorated with a 15 nm-thick Pd layer on the surface [61]. The SnSe combination belongs to the IV–VI family and forms heterojunctions with enhanced electrical transporting characteristics at the interface. Figure 9 shows a typical I–V curve for the heterojunction of the sensor experimentally. The heterojunction exhibits an apparent rectifying behaviour with a large rectification ratio, indicating the formation of a high-energy barrier near the interface of the heterojunction. When H_2_ is released into a test environment filled with pure N_2_, the response (*S*) of the device is defined by the following equation:

(8) $$S=\frac{I_{H}}{I_{N}}$$

where *I_H_* and *I_N_* are the currents under the H_2_ and N_2_ atmospheres. According to the calculation in the paper [62], the fabricated Pd decorated with SnSe/SiO_2_/Si heterojunction shows an ultra-high response to H_2_ at room temperature. Its response at 1 V is ~3225 with an ultra-low detection limit of 0.91 ppb, which was calculated using the equation in [60]:

(9) $$LD=\frac{3\sigma_{noise}}{s}$$

where *LD* is the detection limit, $\sigma_{noise}$ is the root mean square of noise, and the denominator s is the slope of the response versus concentration plot. 

As shown in Figure 9, the heterojunction responses to N_2_, O_2_, NH_3_, humidity, ethanol and acetone are approximately 1.02, 1.11, 1.21, 1.01, 1.30 and 1.07, respectively, which are much lower than the heterojunction’s response towards H_2_ (~3225). This quick response shows that the heterojunction exhibits good selectivity for H_2_.

Despite having several advantages mentioned above, Pd-based thin-film hydrogen sensors also have their disadvantages. For example, the hydrogen embrittlement susceptibility caused by lattice expansion of PdH_x_ intermediates may cause Pd membrane cracking [62]. As well as an irreversible structure, changes after several sensing cycles induced by multiple α-β phase transitions of the PdH_x_ intermediates can cause mechanical stresses on the resistors, which in return can lead to deformation, delamination, and a reduction in sensing durability [38,63]. 

Many researchers have attached significance to Pd- and its composites-based nanowires and nanoparticles [64,65,66,67,68,69,70,71,72,73,74,75,76,77,78,79,80,81,82,83,84,85,86,87,88,89,90,91,92,93]. These materials have unique physical and chemical properties. Compared to the thin sensing films, these nanowires and nanoparticles have a larger surface–volume ratio, more enhanced sensitivity, lower power consumption, faster sensing response and better durability. The ratio of length to diameter is defined as the aspect ratio. The nanostructures with aspect ratios less than 10 are called nanorods (NRs), the aspect ratios more than 10 are called nanowires (NWs), and additionally, hollow NWs are called nanotubes (NTs). 

**Figure 10:**
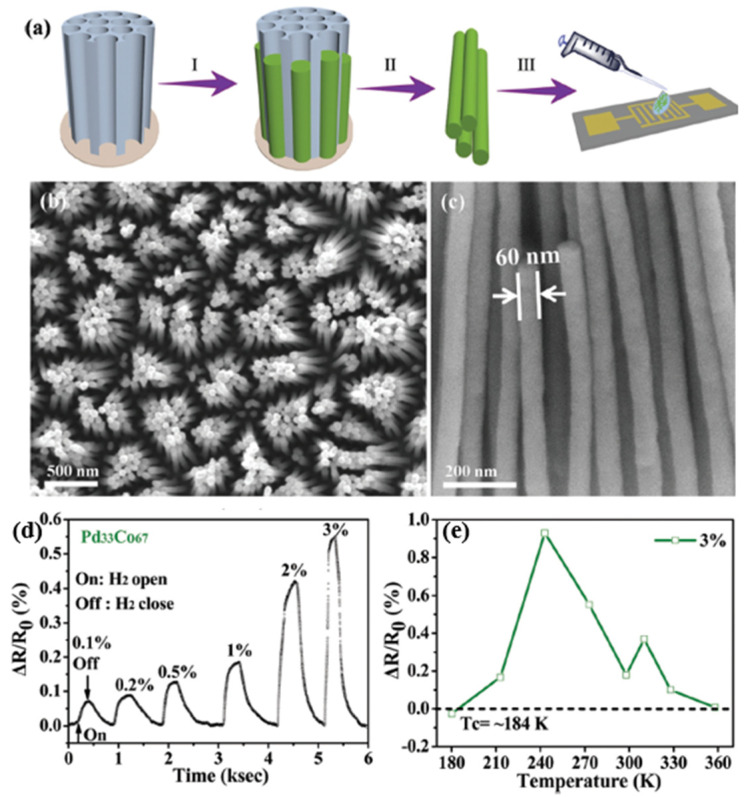
(**a**) Schematic for the synthesis and integration of PdCo nanowires (NWs) including (I) electrodeposition, (II) removal of anode aluminium oxide (AAO) and (III) integration. (**b**) The top-view and (**c**) side-view scanning electron microscope (SEM) images of PdCo NWs arrays. (**d**) The hydrogen sensing response of Pd33Co67 to 0.1–3% H_2_ at 273 K and (**e**) its temperature-dependent hydrogen response [64]. Copyright (2019), with permission from Royal Society of Chemistry.

Du et al. investigated PdCo NW sensors for hydrogen gas sensing over a wide range of temperatures [64]. As shown in Figure 10, the PdCo NW (diameter 60 nm) was fabricated by electrodeposition within an anode aluminium oxide (AAO) nanochannel and further integrated on the test interdigital electrodes (IDE). The sensing performance and stability can be modified by tuning the cobalt Co dopant content. Compared with different Co contents, ${\mathrm{Pd}}_{33}{\mathrm{Co}}_{67}$ has a better sensitivity of about 0.2% but a slower response/recovery time of over 200 s/500 s, at 1% H_2_ concentration at a temperature of 273 K, as shown in Figure 10d. Interestingly, the response of the PdCo NW decreases with increasing temperature. The critical temperature $T_{c}$ for the “reverse sensing behavior” of PdCo NWs is far below room temperature compared to the pristine Pd NWs ($T_{c}$ = 287 K) [65], see Figure 10e. The improvement expanded the low-temperature range and stability of hydrogen sensing.

**Figure 11:**
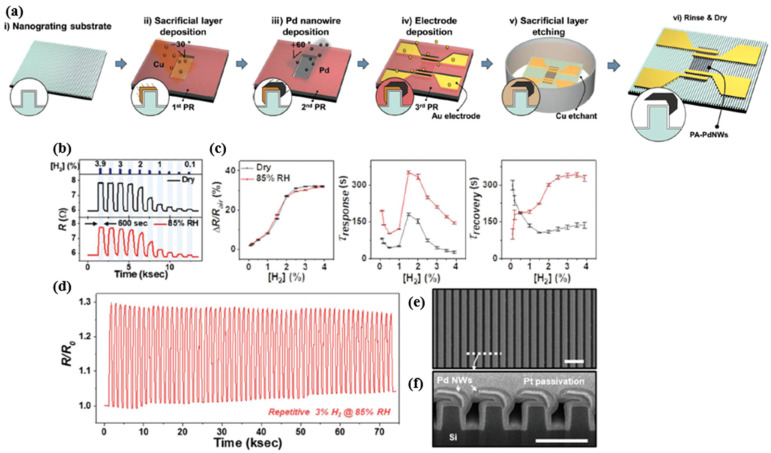
(**a**) Schematic illustration of the fabrication of partially anchored Pd nanowires (PA-PdNW) H_2_ sensor. (Insets: a cross-sectional view of a single nanograting pattern.). (**b**) Measured responses of the PA-PdNW sensors (tw ∼20 nm, la ∼50 nm, wire length equals 20 μm, the nanowire array width equals 30 μm) at varying H_2_ concentrations and different humidity levels. (**c**) The performance of the PA-PdNW sensors extracted from (**b**), namely the gas response, response time and recovery time, respectively. (**d**) Response to repetitive 3% H_2_ exposures at 85% relative humidity. (**e**) Top-view (scale bar equals to 1 μm) and (**f**) cross-sectional (scale bar = 500 nm) SEM images of PA-PdNW after over 50 times repetitive 3% H_2_ exposures of at 85% relative humidity (single nanograting pattern) [66]. Copyright (2019), with permission from Royal Society of Chemistry.

Lee et al. proposed a partially anchored Pd nanowire (PA-PdNW) to enhance the durability of Pd nanowire-based H_2_ sensors. Because most of the structure was not restrained from the volume expansion, PA-PdNW could relieve the internal mechanical stress and thus improve the reliability of the sensor device even it was repeatedly exposed to high concentrations of H_2_ [66]. Schematically, Figure 11a demonstrates the fabrication process of the PA-PdNW H_2_ sensor. Aluminium oxide was first deposited on a silicon nanograting and then further deposited with copper (Cu) at an oblique angle during the process. After selective etching, the Cu layer was processed by Pd-deposition for the sensing, forming a partially anchored and suspended Pd nanowire structure. Then, the Cu layer was removed as the sacrificial layer. Figure 11d shows the sensing response of Pa-PdNW sensors in dry and 85% relative humidity (RH) environments. However, the sensors showed no significant sensitivity difference in both cases. Although the solubility of H_2_ in Pd was almost unaffected by the two different humidity levels, the a-β-phase transition could still be observed at H_2_ concentrations greater than or equal to 2.5%. On the other side, the response/recovery time increased significantly with increasing humidity due to the thin water layer on the surface that delays the hydrogen absorption [67]. Increasing the temperature of the nanowire by applying a heater can significantly reduce the delaying effect caused by high humidity [68]. 

Additionally, the repeatability of the sensor properties performed stably after 50 cycles of β-phase transitions tests in Figure 11d. The SEM images in Figure 11e,f demonstrate the robustness of the partially anchored nanowire. This work in [66] provides a unique structural view for designing a Pd NW-based hydrogen sensor with high durability.

**Figure 12:**
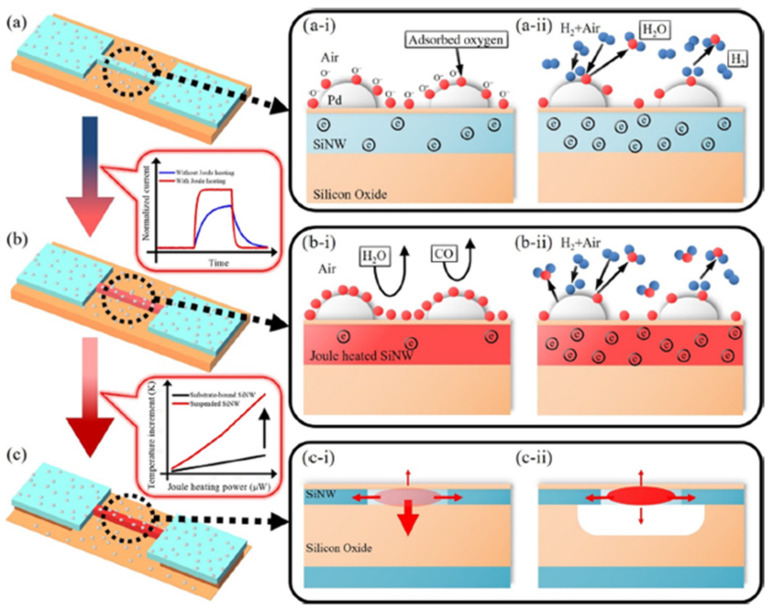
Working principle of H_2_ sensing of a Pd-SiNW. (**a**) At room temperature, (**a**-**i**) depletion of charge carrier (electron) in SiNW (n-type) by negatively charged adsorbed oxygen ions (red dots) and (**a**-**ii**) accumulation of charge carrier by desorbing oxygen with H_2_O formation under H_2_ gas exposure. (**b**) Faster and higher response with Joule heating of Pd SiNW because (**b**-**i**) more adsorbed oxygen causes more depletion of charge carrier and (**b**-**ii**) it has a fast reaction rate with H_2_, but a low interfering gas effect (H_2_O and CO) with Joule heating. (**c**) By replacing substrate-bound SiNW with suspended SiNW, the heat loss through the substrate is reduced, resulting in lower power consumption [69]. Copyright (2017), with permission from American Chemical Society.

In order to improve the sensitivity while lowering the power consumption, Yun et al. [69] developed self-heating, suspended, and palladium-decorated silicon nanowires (Pd-SiNWs) for high-performance hydrogen (H_2_) gas sensing. The suspended SiNWs can significantly lower the power consumption by reducing the heat loss through the substrate. Surface modification with Pd can react with H_2_ at room temperature, but this reaction is slow and can be influenced by humidity or carbon monoxide in the environment (see Figure 12a). Figure 12b,c show the Joule-heating mechanism of the self-heating and suspended structures at lower power consumption, increasing the reaction rate and reducing the degradation caused by humidity or carbon monoxide gas. According to available studies [70,71], the sensitivities of Pd nanowires for H_2_ gas are lowered when applying Joule heating because of the low solubility of H_2_ at high temperatures. In this work [69], Yun et al. developed a Joule heating method for Pd-SiNW, which can accelerate the response to H_2_ gas without sacrificing sensitivity. 

**Figure 13:**
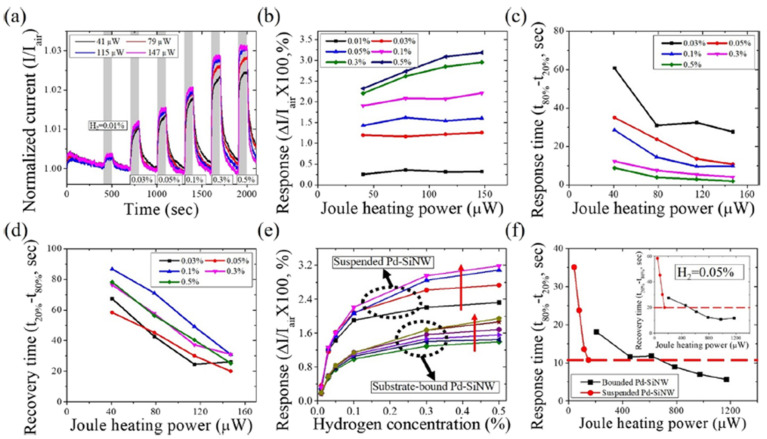
H_2_ gas sensing of the suspended Pd-SiNW sensor: (**a**) normalized current with Joule heating powers of 41, 79, 115, and 147 μW. The (**b**) response, (**c**) response time and (**d**) recovery time for variant H_2_ concentrations at four Joule heating powers. Comparison between the substrate-bound and suspended Pd-SiNW sensors: (**e**) responses at various Joule heating powers (red arrows: direction of Joule heating power increment (from 41 to 147 μW for the suspended Pd-SiNW and from 205 to 1172 μW for the substrate-bound Pd-SiNW)) and (**f**) response time and recovery time (inset in (**f**)) for 0.05% H_2_ (red dotted line indicates the similarity of transient behaviour between the suspended Pd-SiNW at a Joule heating power of 147 and the substrate-bound Pd-SiNW at a Joule heating power of 613 μW) [69]. Copyright (2019), with permission from American Chemical Society.

Figure 13 presents the H_2_ gas response of the suspended Pd-SiNW sensor to H_2_ at different Joule heating powers for H_2_ concentration from 0.01% to 0.5% under the varying Joule heating powers. The sensing performance of both suspended Pd-SiNW and substrate-bound Pd-SiNW is improved without sacrificing response due to the application of the Joule heating. Additionally, as shown in Figure 4e, the suspended Pd-SiNW sensor has a significantly higher response and lower conductivity than the substrate-bound Pd-SiNW sensor. In Figure 13f, the suspended Pd-SiNW at a Joule heating power of 147 μW has a similar transient response to the substrate-bound Pd-SiNW at a Joule heating power of 613 μW, indicating that at the same Joule heating power, the temperature of the suspending SiNW on the substrate is higher than that of the substrate-bound SiNW.

Cho et al. investigated the network structure of a half-pipe palladium nanotube (H-PdNTH) by using electrospinning and e-beam evaporation for high-performance hydrogen gas sensing applications [72]. The H_2_ gas sensing test revealed that reducing the thickness of the H-PdNTH is the main factor to improve the H_2_ sensing sensitivity and response time. The effect of the Pt catalyst on the H_2_ sensing performance was also investigated in the paper. In comparison, the Pt decorated H-PdNTN sensors respond nearly two times faster than that of the pristine H-PdNTN sensors. Wang et al. developed a SAW hydrogen gas sensor by employing Pd and Cu-coated nanowires (Pd/Cu NWs) as the sensitive interface [73]. The electrodeposited Pd/Cu was synthesized inside an AAO template. Their sensor was tested with fast response and recovery time within 4 s, a low detection limit of 7 ppm, and a sensitivity of 1.5 kHz/% at room temperature. Baek et al. decorated Pd on n- and p-type Si NW arrays to improve the sensing performance of H_2_ [74]. Their experiments indicated that the p-type Si NW arrays had ultra-high H_2_ sensitivity of 1700% at 1% H_2_ concentration, which was higher than that of the n-type Si NW arrays of 75% at 1% H_2_. This sensitivity difference was because the Schottky barrier in the n-type Si NW arrays changes to ohmic contact when exposed to H_2_, and the interface effect of Pd/Si diminishes with increasing H_2_ concentration. On the other hand, the ohmic contact in the p-type Si NW arrays changes to a Schottky barrier when exposed to H_2_. As the H_2_ concentration increases, the work function of PdH_x_ decreases, increasing the height of the Schottky barrier in the p-type Si NW arrays, thereby improving its sensitivity. 

**Figure 14:**
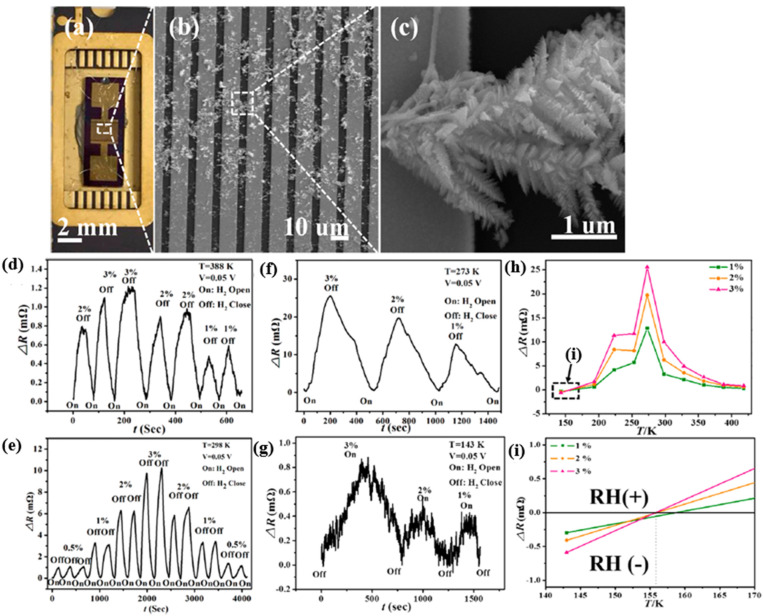
A hydrogen sensor of Pd/Bi/Cu HNAs, SEM images and elemental mapping analysis. (**a**) The sensor prototype. (**b**,**c**) SEM images of Pd/Bi/Cu HNAs on IDE electrode and the magnified SEM image from the dashed rectangle in (**b**). Response of hydrogen sensor integrated with multiple HNAs. (**d**–**g**) ΔR plots with the representative temperature at 388 K, 298 K, 273 K, and 143 K, respectively. (**h**,**i**) The summarized temperature-dependent ΔR plots of Pd/Bi/Cu HNAs sensors. The resistance modes, negative values (below T_C), corresponds to the RH (−) mode and positive ones to the RH (+) mode. Here, on: introducing Hydrogen, off: venting hydrogen, ΔR: alteration of resistance [79]. Copyright (2019), with permission from American Chemical Society.

Zheng et al. investigated palladium/bismuth/copper hierarchical nano architectures (Pd/Bi/Cu HNAs) for a hydrogen evolution reaction (HER) and hydrogen sensing applications [76]. In their work, the Pd/Bi/Cu NWs were synthesized by electrochemical deposition inside the nanochannels of AAO templates, forming “forest”-like crystalline microstructures, as shown in Figure 14a–c. The “trunk” of the hierarchical structure is around 2.5 µm long, and 200 nm-long secondary branches serve as a second “trunk”, generating further branches in succession. This kind of microstructures expanded the contact surface for hydrogen sensing via creating more chemical reactive sites. Furthermore, the author investigated the temperature-dependent “reversing sensing behaviour“, which changed the resistance sensing response at the critical temperature. Such a behaviour has been reported and explained by the fact that the crystalline transformation during the α-β phase transition in the hydride process generates new percolation paths that reduce the necessary distance for electrons to move through different percolation paths [65]. At the critical temperature, the sensing response appears as a negative resistance change ΔR (RH(−)). 

In contrast, for the working temperature above the critical temperature, the sensing response presented itself as a positive resistance change ΔR (RH(+)), as shown in Figure 14h,i. Compared with pristine Pd nanowires (NWs, 278 K), the designed microstructure of the Pd/Bi/Cu combination reduced the critical temperature of the “reversing sensing behaviour“ to about 156 K and extended the operating temperature range (about 156 to 418 K). The sensing response is illustrated in Figure 14d–i.

Pd-coated SnO_2_ nanofiber rods (NFRs) synthesized by electrospinning and magnet sputtering were introduced by Wang et al. in 2020 [77]. The deposited Pd played a role as a catalyst to improve the sensing performance. The gas sensors enhanced the sensing response at a temperature (160 °C) lower than the SnO_2_-based hydrogen gas sensor and obtained a fast response with a response time of 4 s at 100 ppm H_2_ concentration. The limit of detection can be achieved at an ultra-low hydrogen concentration of 0.25 ppm. 

**Figure 15:**
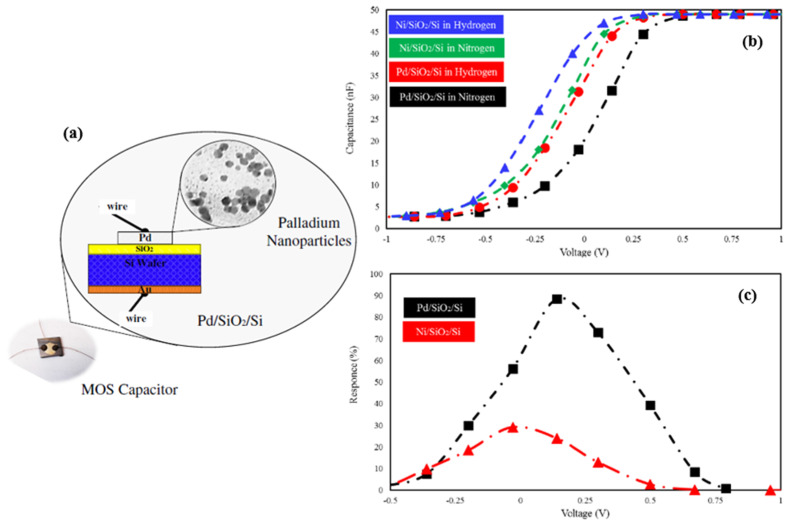
(**a**) Schematic diagram of the Pd/SiO_2_/Si capacitor. (**b**) Experimental values of the C–V curves for the Pd/SiO_2_/Si sensor at room temperature and the Ni/SiO_2_/Si sensor at 100 kHz frequency in a mixture of pure nitrogen and 1% H_2_–N_2_ at 140 °C. (**c**) The response (%) of the Pd/SiO_2_/Si and Ni/SiO_2_/Si MOS capacitor sensors in 1% H_2_–N_2_ mixtures [79]. Copyright (2017), with permission from World Scientific.

Generally, Pd nanoparticles were deposited on the substrate to enhance sensing performance towards hydrogen. Kumar et al. in 2015 deposited Pd layers directly on the SiO_2_/Si substrate by evaporating Pd for hydrogen gas detection [78]. After deposition, the samples were annealed at 400 °C for 2 h to form the isolated Pd nanoparticles. As investigated in the paper, the annealed sensor formed with Pd layers of thickness 1 nm and 5 nm had no sensing response during the release of H_2_. The Pd nanoparticles were isolated after annealing, and no electrical pathways were formed to transfer sensing signals. Up to 10 nm thickness, the annealing process formed electrical “channels” on the substrate leading to a sensing performance of 51.4% response at 2% H_2_ concentration in the test environment. The response time for the detection was 6 s, and the recovery time was 45 s. In contrast, Behzadi Pour et al. in 2017 investigated a hydrogen sensor based on a capacitive sensing mechanism synthesized by depositing palladium nanoparticles on a silicon oxide surface, as shown in Figure 15 [79]. For this type of metal-oxide-semiconductor (MOS) capacitor, the capacitance can be obtained by [80]:

(10) $$C={\left(\frac{t_{\mathrm{OX}}}{\varepsilon_{\mathrm{OX}}\varepsilon_{0}A}+\frac{X_{d}}{\varepsilon_{s}\varepsilon_{0}}A\right)}^{-1}$$

where $\varepsilon_{\mathrm{OX}}$ and $t_{\mathrm{OX}}$ are the relative permittivity and thickness of the oxide film, respectively, $\varepsilon_{s}$ is the relative permittivity of Si, A is the area of the Pd gate, and $X_{d}$ is the width of the depletion region. In an ideal MOS capacitor, the $V_{\mathrm{FB}}$ is defined as the boundary between accumulation and depletion regions as:

(11) $$V_{FB}=\frac{W_{MS}}{q}-\frac{Q}{C_{OX}}$$

where $W_{MS}$ is the work function difference between gate and substrate, and *Q* is the trapped charges in the SiO_2_ film. For Pd/SiO_2_/Si sensors, the $V_{FB}$ is 0.7 V. Thus, the capacitance of the MOS capacitor in the depletion layer can be obtained [81]:

(12) $$C_{T}=\frac{C_{OX}}{\sqrt{1+\frac{2\varepsilon_{\mathrm{OX}}^{2}\varepsilon_{0}\left|V-V_{\mathrm{FB}}\right|}{{\mathrm{qN}}_{D}\varepsilon_{s}t_{\mathrm{OX}}^{2}}}}$$

where $N_{D}$ is the donor carrier density in Si, and q is the charge of the electron. This gas sensor had a fast response time of 1.4 s and a recovery time of 14 s with a sensitive response of 88% at 1% H_2_ concentration.

**Figure 16:**
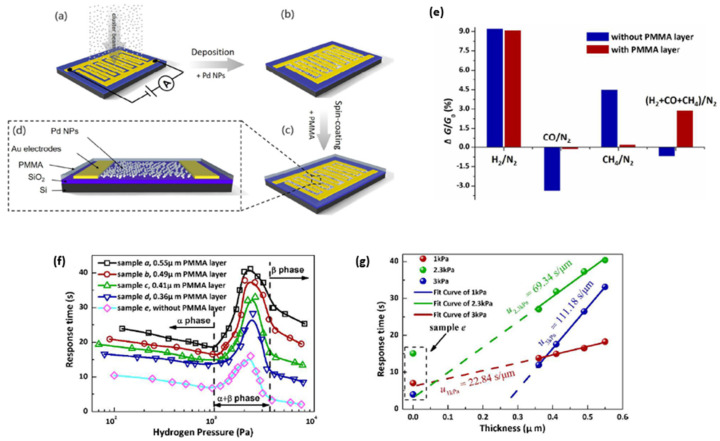
Schematic illustration of the procedure used to fabricate the hydrogen sensor based on a poly (methyl methacrylate) (PMMA)-membrane-coated Pd nanoparticle (NP) film. (**a**) The deposition of Pd NPs. (**b**) The Pd NP film formed on the interdigital electrode substrate after the NP deposition. (**c**) The PMMA-membrane-coated Pd NP film fabricated by spin coating. (**d**) The structure of the hydrogen sensor based on the PMMA-membrane coated Pd NP film. (**e**) The response of sensors with and without a PMMA membrane layer to target gas mixtures. The concentration of CO/N_2_, CH_4_/N_2_ and H_2_/N_2_ was 1000 ppm. The tested mixed gas of H_2_, CO and CH_4_ in N_2_ was formed by simultaneously filling the three kinds of gas into the test chamber. (**f**) Response time as a function of hydrogen pressure for five samples of different thicknesses (**a**–**e**). The dashed lines indicate the boundaries of the three hydride phase regions. (**g**) Plots of the response time versus the thickness of the PMMA membrane layer [82]. Copyright (2017), with permission from American Chemical Society.

Despite the advantages of employing Pd nanoparticles for hydrogen gas sensing, the low selectivity may reduce the sensor’s sensitivity towards hydrogen gas. Chen et al. in 2017 [82] investigated the H_2_ gas sensor with Pd nanoparticles deposited on SiO_2_/Si substrates for different target gases and gas mixtures. Without any filtration layer, this H_2_ gas sensor cannot accurately distinguish between cross-sensitive gases and gas mixtures such as carbon monoxide (CO), water vapour and methane (CH_4_). More importantly, CO is a highly toxic cross-sensitive gas for Pd catalysts, which causes a reduction in available dissociation sites on the Pd metal surface due to CO adsorption [83,84]. To address this issue, Lee et al. in [85] reported that an H_2_ sensor using a hybrid structure of Pd NP/single-layer graphene (SLG) coated with a polymer membrane achieved high selectivity towards H_2_. In comparison, Chen et al. reported in [82] that H_2_ sensors with a thin spin-coated layer of poly (methyl methacrylate) (PMMA) on the sensing surface deposited by Pd NP significantly improved the gas selectivity towards H_2_, as shown in Figure 16e. Due to the thickness of the PMMA membrane layers, the sensor response became correspondingly slower. This retardation effect of the sensing kinetics during the sensing reaction is mainly attributed to three mechanisms: the diffusion of H_2_ gas in the PMMA matrix, the nucleation and growth of the β phase in the α phase matrix of Pd hydride, and the stress relaxation at the interface between Pd NPs and the PMMA matrix. Accordingly, the response time was experimentally proved to be correlated with hydrogen pressure and the thickness of the PMMA layer, as presented in Figure 16f,g.

Even though Pd NPs can be deposited in hydrogen gas sensors as catalysts in hydrogen detection, their combination with metal oxide structures can still improve the sensing response by enhancing the electron conductive path. Much literature has discussed the use of Pd NPs decorated with various metal oxide structures for hydrogen gas sensing, such as ZnO [86], SnO_2_ [87,88], WO_3_ [89,90], MnO_2_ [91], and MoS_2_ [92].

A flexible hydrogen sensor based on ZnO nanorods (NRs) was developed by Rashid et al. in 2014 [86]. The ZnO NRs were synthesized from Ga-modified ZnO seed layers (GZO). The dopant Ga atoms were incorporated into ZnO material by replacing the Zn host atoms, releasing free electrons, and contributing to high conduction or improving carrier mobility. This kind of sensor (ZnO: 3% Ga-doped seed) is reported to have a high response factor of 91.2% and a fast response time of 18.8 s. Additionally, it also exhibits high selectivity when exposed to different target gases. In paper [93], Weber et al. in 2019 developed an H_2_ gas sensor based on ZnO NWs coated with a thin layer of boron nitride (BN) and decorated with Pd NPs, whereas the Pd NPs help the BNs to enhance the performance of different catalytic reactions [94,95]. It has been found that the hydrogen’s lowest detection level could be 0.5 ppm with a sensitivity of 1.34 at 200 °C. The Pd/BN/ZnO NWs responded at an H_2_ concentration of 100 ppm within an H_2_ releasing time of 160 s and a recovery time of 90 s ($R_{a}/R_{g}$ = 12.28).

**Figure 17:**
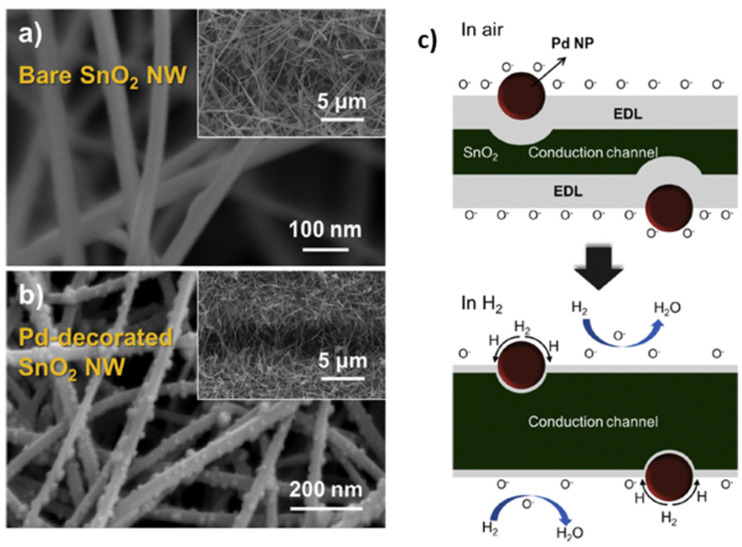
Field-emission scanning electron microscope (FE-SEM) micrographs of (**a**) bare SnO_2_ NWs and (**b**) Pd-decorated SnO_2_ NWs. Upper-right inserts are the top-view of the FE-SEM images. (**c**) The sensing mechanism of Pd-decorated SnO_2_ NWs [87]. Copyright (2017), with permission from Elsevier.

For the gas sensor based on the combination of SnO_2_ and Pd NPs, Li et al. [87] in 2017 synthesized SnO_2_-composite Pd nanoparticles with average diameters up to hundreds of nanometers via a solvothermal method. The hydrogen sensor using 10 mol% Pd-SnO_2_ composites showed enhanced performance compared to pristine SnO_2_. At 200 °C working temperature, the Pd-SnO_2_ composite-based hydrogen sensor performed a fast response/recovery time of 4s/10s and high sensitivity ($R_{a}/R_{g}$ = 315.34) for 3000 ppm H_2_ concentration. The lowest detection limit was achieved at an ultra-low concentration of about 10 ppm. By contrast with the work in [87], Kim et al. in 2019 synthesized in [88] Pd NPs-decorated SnO_2_ NWs for hydrogen sensing applications, as shown in Figure 17. The presence of Pd on the NW surface increased the electron depletion region of SnO_2_ NWs, which modulated the conduction channels inside NWs. Therefore, the initial conduction volume or initial electron concentration of SnO_2_ decreased accordingly. When H_2_ gas was adsorbed onto SnO_2_, it raised electron concentration at the surface and assisted in further elevating the Fermi level, which increased the potential barrier at the SnO_2_/Pd interface. This change at the surface made it difficult for electrons in SnO_2_ to move to the Pd side, decreasing the resistance in SnO_2_ and improving the sensing response, as presented in Figure 17c. The gas test results showed that the response of the decorated SnO_2_ NWs to 1 ppm H_2_ concentration at 300 °C increased from 6.88 to 16.95 ($R_{a}/R_{g}$).

**Figure 18:**
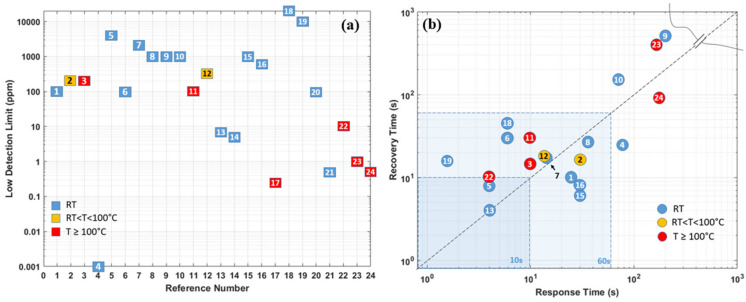
Regarding Table 1, schematic illustrations of the comparison of the reported Pd-based hydrogen gas sensors in literature: (**a**) low detection limits. The x-axis “Reference Number” represents the 1st column“Ref. Nr.“ in Table 1. (**b**) Response time/recovery time under experimental conditions as reported in the literature.

As the summary in the section of Pd-based hydrogen gas sensors, Table 1 compares the sensing performances based on different material combinations, operating temperatures, synthesis methods, low detection limits, and sensing responses (including response time/recovery time, sensitivity at target hydrogen concentration). Furthermore, the sensor performance responsible parameters are also schematically illustrated in Figure 18a,b. In general, Pd and its combination-based gas sensors can detect hydrogen gas at room temperature, and the size effect affects the sensing performance significantly. The smaller the sensing structure/cluster is, the more significant the sensor’s reactive surface, which promises better sensing performance towards hydrogen with lower power consumption. However, the low ambient operating temperature can raise the humidity rate on the sensor surface, which reduces the sensor response and sensitivity. Additionally, the small size and discontinuity of the sensing materials also lead to a very low conductivity of the nanostructures, which induces high electrical noise, and degrades the sensor response. 

## 3. Metal Oxide Semiconductor-Based Hydrogen Gas Sensors

Another classification of promising materials for gas sensing is the conductometric metal oxide semiconductor (MOS). They are generally crystalline or polycrystalline in nature and interconnected with different grains having grain boundaries. However, by contrast with transition metals, most metal oxide materials are essentially insulators at room temperature. As a result, they cannot be used directly for gas sensing due to their bandgaps between the conduction band and valence bands forming electron mobility barriers. Typically, the gas sensing suitable metal oxides have bandgap between 2.7 eV and 4 eV, like SnO_2_ (3.6 eV), NiO (3.5 eV), TiO_2_ (3.2 eV), ZnO (3.2 eV), WO_3_(2.7 eV) etc. [96,97]. With an elevated temperature in the range of up to 600 °C [98], the amount of mobile electrons can be generated by electrons thermally from the valance band into the conduction band participating in the charge transfer.

Meanwhile, the interaction between the MOS sensing materials and the surrounding chemical environment also affects the mobile carrier density inside metal oxide materials and changes their resistance consequently. In their gas sensing mechanisms, depending on the target gas (oxidizing or reducing) and semiconductor type (n- or p-type), the resistance can increase or decrease during the interaction with the target gas. In the case of n-type metal oxides with electrons (e-) as majority carriers, the resistance increases in oxidizing gases (acceptor) but decreases in reducing ones (donor), whereas the p-type metal oxide (e.g., NiO [99]) with holes as majority charge carriers has the converse resistance changes [100].

**Figure 19:**
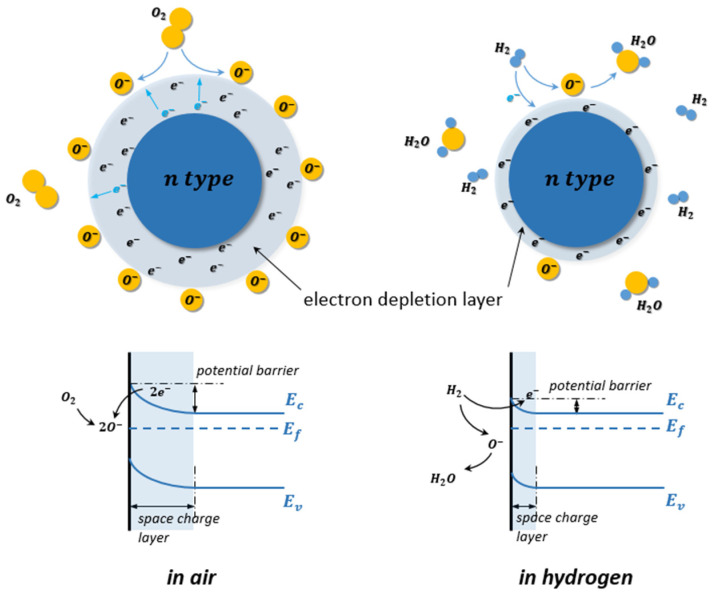
Schematic illustration of the gas sensing mechanism of n-type metal oxide semiconductor (MOS) and its energy band change in the air and hydrogen.

Taking n-type MOS such as SnO_2_ or TiO_2,_ for instance, at elevated temperature in the air atmosphere, the oxygen molecules (O_2_) are chemisorbed on the surface of the MOS, as shown in Figure 19. Since O_2_ molecules are oxidizing gas and act as acceptors of electrons, they are dissociated on the surface and ionized into species such as $O_{2}^{-}$, $O^{-}$, and $O^{2-}$ by trapping electrons from the conduction band of the n-type MOS, as indicated in Equations (1)–(4). The ionizations of these chemisorbed species are temperature dependent. The $O_{2}^{-}$ is dominant at temperatures lower than 150 °C, $O^{-}$ has its range from 150 to 400 °C, and $O^{2-}$ is at temperatures larger than 400 °C [101,102].

(13) $$O_{2\left(\mathrm{gas}\right)\;}\;\to {\;O}_{2\left(\mathrm{ads}\right)}$$

(14) $$O_{2\left(\mathrm{ads}\right)}+{\;e}^{-}\;\to {\;O}_{2\left(\mathrm{ads}\right)}^{-}$$

(15) $$O_{2\left(\mathrm{ads}\right)}^{-}+{\;e}^{-}\;\to 2O_{\left(\mathrm{ads}\right)}^{-}$$

(16) $$O_{\left(\mathrm{ads}\right)}^{-}+{\;e}^{-}\;\to {\;O}_{\left(\mathrm{ads}\right)}^{2-}$$

Due to the decrease of electron density of the n-type MOS, an electron depletion layer has been created around the MOS with a high potential barrier. This electron depletion layer leads to an electronic core–shell configuration. As the electron conduction of the MOS decreases, its resistance is therefore increased.

When reducing gases, such as $H_{2}$ are exposed to the MOS, the chemisorbed oxygen ion species on the MOS surface react with $H_{2}$, as indicated in Equations (17)–(19) by forming $H_{2}O$ and releasing electrons back into the electron depletion layer.

(17) $$2H_{2}+{\;O}_{2\left(\mathrm{ads}\right)}^{-}\;\to 2H_{2}O+{\;e}^{-}$$

(18) $$H_{2}+{\;O}_{\left(\mathrm{ads}\right)}^{-}\;\to {\;H}_{2}O+{\;e}^{-}$$

(19) $$H_{2}+{\;O}_{\left(\mathrm{ads}\right)}^{2-}\;\to {\;H}_{2}O+2e^{-}$$

Thus, the thickness of the electron depletion layer becomes thinner, accompanying the decrease of the resistance of the MOS hydrogen gas sensor [103].

**Figure 20:**
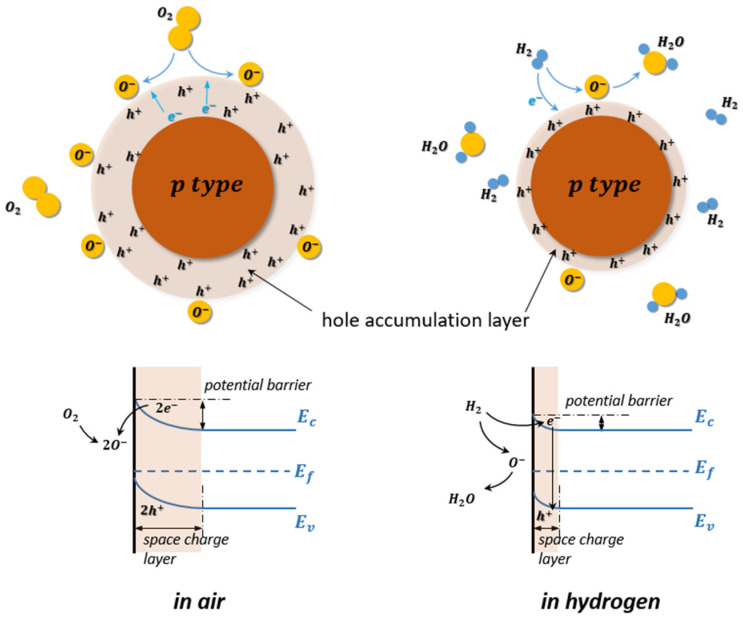
Schematic illustration of the gas sensing mechanism of p-type MOS and its energy band change in the air and hydrogen.

In contrast, when the p-type MOS hydrogen gas sensor is exposed at elevated temperature in the air atmosphere, the electrons from the valence band are captured on the surface, and a hole accumulation layer is formed around the p-type MOS core. Consequently, the resistance of the MOS hydrogen gas sensor decreases. Similarly, the chemisorption of air oxygen and ionization process can also be described with Equations (1)–(4). With $H_{2}$ as the target gas releasing in the test environment, the $H_{2}$ molecules react with oxygen ion species similarly and release electrons back into the hole accumulation layers. The receptor site accommodates electrons from the conductance band and causes a shrink of the hole accumulation layer, which leads to a resistance increase of the MOS gas sensor, as shown in Figure 20 [104]. Similar to the case for the n-type MOS gas sensor, the Equations (17)–(19) are also used to describe the reduction process for the p-type MOS gas sensor.

Since the MOS gas sensors can exchange electrons with a wide variety of gases, the selectivity of MOS gas sensors is inherently limited. Thus, optimization measures are also required to adjust the performance, such as a mixture with noble metal catalysts on the MOS surface, like palladium and platinum [87]. Furthermore, the optimization of microstructure and nanostructure can also improve the sensibility and response of the MOS gas sensors [105,106,107].

A Joule effect heating element is needed to raise the oxide layer’s temperature, to promote surface reactions, and to remove water produced from these reactions [108].

**Figure 21:**
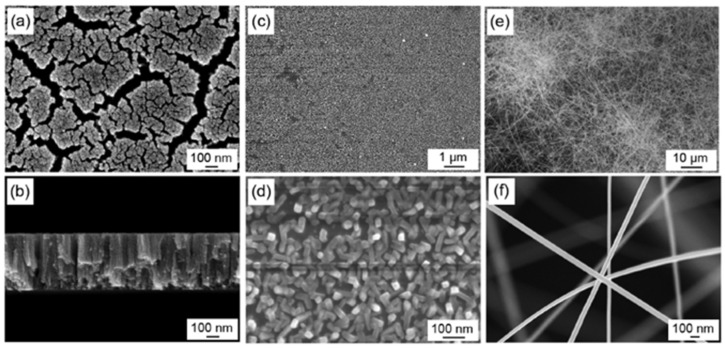
(**a**) Surface and (**b**) cross-sectional field of the emission scanning electron microscopy (FE-SEM) images of SnO_2_ nanofilms. (**c**) Low- and (**d**) high-magnification FE-SEM images of SnO_2_ nanorods. (**e**) Low- and (**f**) high-magnification FE-SEM images of SnO_2_ nanowires [105]. Copyright (2015), with permission from Elsevier.

**Figure 22:**
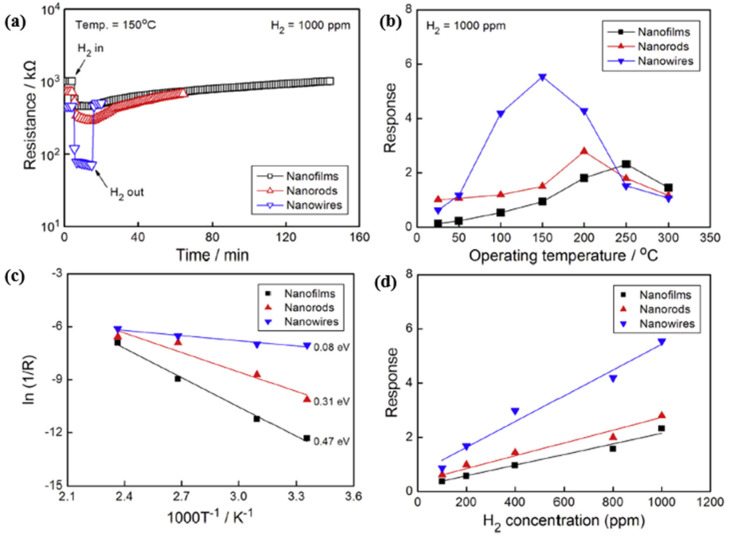
(**a**) Changes in the resistance of gas sensors based on SnO_2_ nanomaterials upon exposure to 1000 ppm H_2_ at an operating temperature of 150 °C. (**b**) Responses of SnO_2_ nanomaterials upon exposure to 1000 ppm H_2_ as a function of the operating temperature. (**c**) Arrhenius plots representing the electrical conductivities of SnO_2_ nanomaterials before H_2_ introduction versus the reciprocal of the operating temperature (1/T). (**d**) Relationships between the response and H_2_ concentration for SnO_2_ nanofilms at 250 °C, SnO_2_ nanorods at 200 °C, and SnO_2_ nanowires at 150 °C [105]. Copyright (2015), with permission from Elsevier.

SnO_2_, with a wide bandgap of 3.7 eV at 300 K (about 26.85 °C) [109], is one of the most widely used metal-oxide-semiconductors for gas sensing applications due to its high sensitivity and good stability. However, the SnO_2_ based gas sensors exhibited poor selectivity, long response/recovery time, and a need for high operating temperature, high power consumption and complex integration structure accordingly [110]. To resolve these issues, researchers have devoted their efforts to improve the sensing performance of SnO_2_ by applying various methods, such as morphological modifications [105,106,111] and metallic doping [87,112,113,114,115]. Since the gas-sensing performance of SnO_2_-based gas sensors depends strongly on the fabrication morphology, Shen et al. in [105] investigated nanofilms, nanorods and nanowires at various hydrogen concentrations at high ambient temperatures, as presented in Figure 21a,f. In their work, the SnO_2_ nanofilms were deposited on oxidized Si substrates using the sputtering method, and the SnO_2_ nanorods and nanowires were synthesized by the thermal evaporation method. When the sensors are exposed to H_2_ test gas of 1000 ppm concentration at 150 °C, the nanomaterial demonstrates low sensor resistance and good reversibility due to its sizeable effective surface. The response time and recovery time become shorter as the effective surface area increases. The resistance of the SnO_2_ nanowire is one order of magnitude lower than nanofilms and nanorods. At varying operation temperatures at the same H_2_ concentration, the SnO_2_ nanomaterials had their peak response at different temperatures, namely SnO_2_ nanofilms had 2.3 at 250 °C, SnO_2_ nanorods had 2.8 at 200 °C, and SnO_2_ nanowires had 5.5 at 150 °C. These results revealed that, as the effective surface area of SnO_2_ nanomaterials increases, the peak operating temperature decreases gradually, as shown in Figure 22b.

The relation between the sensor response and operating temperature of an n-type semiconductor has been described by employing the Arrhenius equation [116,117]:

(20) $$\sigma =\sigma_{0}\exp\left(-\frac{E_{a}}{k_{B}T}\right)$$

where σ is the electrical conductivity; $E_{a}$ represents the activation energy, $k_{B}$ is the Boltzmann constant, and T is the operating temperature in Kelvin. As shown in Figure 22c, the authors calculated the activation energies from the Arrhenius plots’ slope over a temperature range of 25–150 °C with values of 0.47 eV for SnO_2_ nanofilms, 0.31 eV for SnO_2_ nanorods, and 0.08 eV for nanowires. These results suggest that smaller activation energy leads to more straightforward sensing reactions and faster sensor responses. Moreover, as Figure 22d shows, there is a relatively linear response for target gas concentrations with increasing H_2_ concentrations at each peak response temperature. Furthermore, the response of SnO_2_ nanofilms, nanorods, and nanowires increases sequentially at the same H_2_ concentrations.

**Figure 23:**
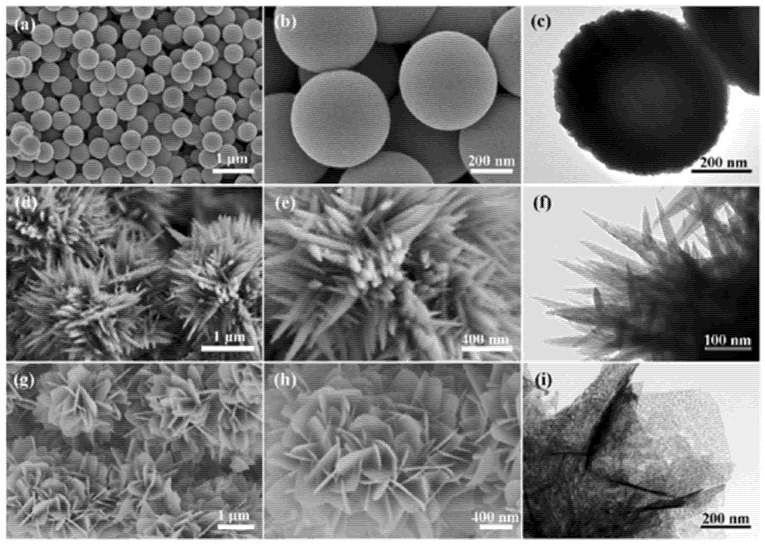
Scanning electron microscope (SEM) and transmission electron microscope (TEM) images of the SnO_2_ samples with different morphologies: (**a**–**c**) solid spheres (S0), (**d**–**f**) nano-needle assembled nano-urchins (S1), (**g**–**i**) nanosheet-assembled nanoflowers (S2) [106]. Copyright (2019), with permission from Elsevier.

**Figure 24:**
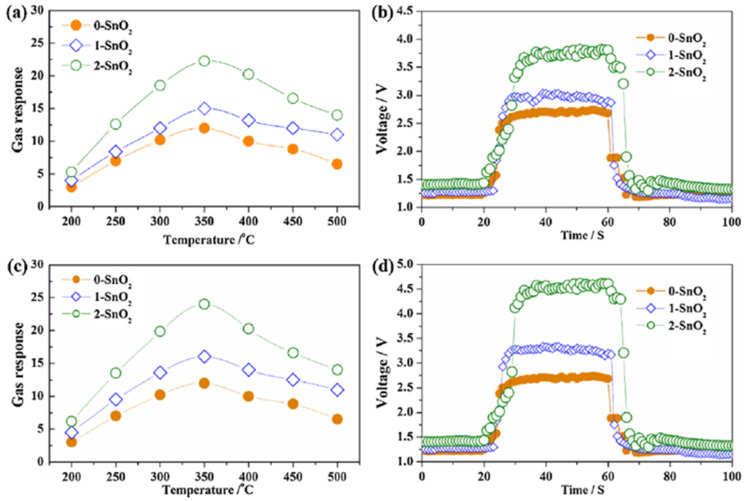
(**a**) Gas response of the three sensors exposed to 400 ppm H_2_ concentration at different temperatures in an atmospheric environment. (**b**) The response-recovery curve of the three sensors under 400 ppm H_2_ concentration at 350 °C in an atmospheric environment. (**c**) Gas response of the three sensors exposed to 400 ppm H_2_ concentration at different temperatures in a vacuum environment. (**d**) The response-recovery curve of the three sensors under 400 ppm H_2_ at 350 °C in a vacuum environment [106]. Copyright (2019), with permission from Elsevier.

Zhu et al. studied the hydrogen detection response of SnO_2_ nanostructures in a non-oxygen environment innovatively [106]. As shown in Figure 23, the three different nanostructures of SnO_2_ were synthesized by a thermal evaporation method: SnO_2_ solid spheres (of ~500 nm diameter) (marked as 0-SnO_2_), the SnO_2_ nano-urchins assembled by a nano-needle (of ~50 nm diameter and ~500 nm length) (marked as 1-SnO_2_), and SnO_2_ nanoflowers assembled by nanosheets (of ~50 nm average thickness) (marked as 2-SnO_2_). Among these three nanostructures, nanoflowers had the best sensing response at 350 °C. The reason behind this is that a nanoflower has the largest effective surface for H_2_ chemisorption in both aerobic/oxygenated and anaerobic/non-oxygenated environments, as shown in Figure 24. A similar trend can also be seen in the change in gas response of the sensors based on the nanostructures, i.e., an increase followed by a decrease with increasing operating temperature.

Interestingly, these sensors in a vacuum environment are more sensitive and have faster response and recovery times than those in the atmospheric environment. Furthermore, the (1 1 0) surface was the most thermodynamically stable among all the low-index SnO_2_ surfaces [118,119]. In turn, the sensing performance of the SnO_2_ nanostructure is strongly affected by its effective surface. Therefore, the authors in [120] investigated SnO_2_ (1 1 0) surface-based sensors’ mechanism in the two vacuums mentioned above and atmospheric test environments. The adsorbed oxygen is widely claimed to be the primary mediator of a gas sensing reaction between reductive gases and sensing materials, and adsorbed oxygen is considered as the electron transfer pathway. However, the results show that only relatively weak electron transfer occurs between the H_2_ and adsorbed oxygen atoms. Furthermore, the H_2_ gas molecule is more likely to react with its oxygen atoms in the SnO_2_ lattices on the surface than with adsorbed oxygen atoms, which is inconsistent with the conventional gas sensing mechanism of n-type semiconductors. Therefore, this discovery can probably open up a new route to improve the gas sensing properties of SnO_2_ and even other sensing materials.

Liu et al. in 2018 developed a hydrogen gas sensor based on hydrothermal synthesized SnO_2_ nanoflowers [111]. This nanostructure increased the effective surface significantly for oxygen adsorption. Additionally, a vacuum annealing treatment in the post-treatment process increased the surface area of SnO_2_ nanoflowers by another 8% and decreased the sensor conductivity. As the annealing process raised the number of active sites for adsorbed oxygen remarkably, the annealed sensor exhibited a sensitivity of about 80.2% for 1000 ppm H_2_ concentration at room temperature, compared to 27.1% for the unannealed one. The annealed sensor required a response time of 71 s, which was slightly slower than the annealed one, of 62 s. However, the recovery time for the annealed sensor was as long as about 500 s, compared to130 s for the unannealed one.

The metallic doped nanoparticles (NPs), such as palladium Pd [87,112], platinum Pt [113,114], gold Au [115], have also been proved to be an efficient method to improve the hydrogen sensing response of metal oxide semiconductor-based gas sensors.

**Figure 25:**
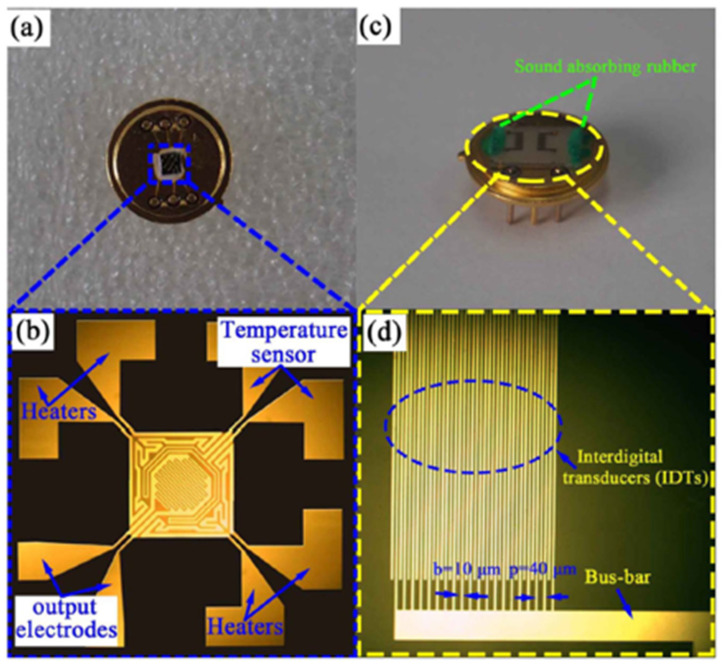
Two kinds of hydrogen gas sensors based on SnO_2_ films: (**a**) the chemi-resistive hydrogen gas sensor, (**b**) the micrograph of the chemi-resistive hydrogen sensor, (**c**) the delay-line surface acoustic waves (SAW) hydrogen gas sensor, and (**d**) the micrograph of interdigital transducers (IDTs) of the SAW hydrogen sensor [112]. Copyright (2017), with permission from Elsevier.

SnO_2_-composited Pd NPs with different Pd loadings (0, 2.5, 7.5, 10 mol% Pd) for hydrogen detection application were investigated by Li et al. [87]. In their work, the sensing materials were synthesized by the solvothermal method and then calcinated. At an operating temperature of 200 °C, for a hydrogen concentration of 3000 ppm, the 10 mol% Pd loaded sensor performed the highest response, with R_a_/R_g_ equal to 315.34 and a fast response/recovery time (4 s/10 s), more than eight times higher than pristine SnO_2_-based sensors. By contrast, Yang et al. in [112] compared Pd NPs-modified SnO_2_ thin-film hydrogen gas sensors synthesized with the sol-gel method and the magnetron sputtering method, as shown in Figure 25a–d. The hydrogen sensing properties were characterized using delay-line surface acoustic waves (SAW). The results showed that the sensor with the RF-sputtered Pd surface-modified layer and DC sputtered SnO_2_ layer obtained the highest sensitivity to 2000 ppm hydrogen gas at 175 °C.

A hydrogen gas sensor based on SnO_2_ nanorods has also been fabricated using a simple hydrothermal method, with the surface of the nanorods decorated with Pt NPs through irradiated photochemical reduction method. Surprisingly, the sensor exhibited a high sensitivity of 88.35% even at room temperature with an ultra-fast sensing response of 0.4 s in time for 1000 ppm hydrogen. This type of Pt-decorated SnO_2_ hydrogen gas sensor can efficiently reduce the power consumption [87], thus ensuring the sensor performance during the long-term operation process [21]. On the other hand, the low ambient temperature enhanced the negative impact of humidity on the sensor. As reported in [98], the entire sensor response decreased from 88.35% at 21.88% relative humidity (RH) to ~26.58% at 83.50% RH, indicating that the sensor response decreased significantly with increasing relative humidity.

Yin et al. [114] used screen-printed Pt-decorated SnO_2_ NPs on alumina substrates for H_2_ gas sensing. Before the screen-printing process, the decorated NPs were prepared by using sol-gel and hydrothermal methods. The results indicated that a 1 wt.% Pt-SnO_2_ sample gave the best sensing response to 100 ppm H_2_ at an optimum working temperature of 350 °C with the lowest detection limit of 0.08 ppm. The ideal response time for this sample was 29 s, and the recovery time was 36 s.

**Figure 26:**
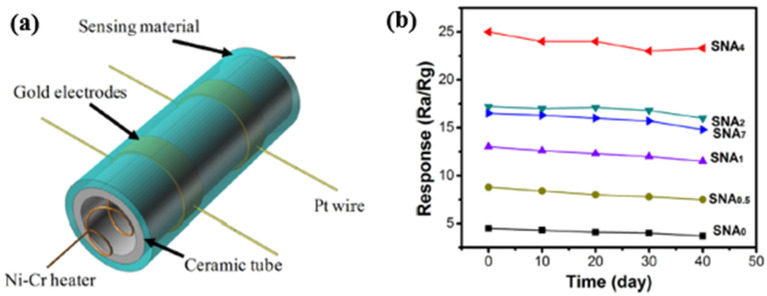
(**a**) Schematic diagram of SnO_2_ gas sensing element. (**b**) Long-term stability of the prepared gas sensors to 100 ppm H_2_ at their optimum temperatures [115]. Copyright (2017), with permission from Elsevier.

In 2017, Wang et al. [115] developed a hydrogen gas sensor based on Au-loaded SnO_2_ composite NPs, (see in Figure 26a). Firstly, the Au-loaded SnO_2_ was mixed with terpineol and ethyl cellulose, grounded with deionized water. Then, the mixture formed a sensing paste, which was coated on a ceramic tube. Next, the paste formed a functional sensing film towards hydrogen with a pair of Au electrodes and Pt wires. After analyzing the effect of Au concentration on sensing properties, it was found that the 4.0 wt.% Au-loaded SnO_2_ gas sensor exhibited a relatively high sensing response at the optimal working temperature of 250 °C. At this temperature, the sensor’s response/recovery time (1/3 s) was fast, and a low detection limit of 1 ppb H_2_ concentration was obtained. The paper [115] also verified the high selectivity of this sensor towards H_2_ and its excellent long-term stability, as presented in Figure 26b.

Another widely used metal oxide semiconductor is ZnO, an n-type semiconductor with a bandgap of 3.37 eV. ZnO-based gas sensors exhibit better performance because of their wide range and direct bandgap, high electron mobility and high excitation bind energy. However, like some SnO_2_-based gas sensors, many ZnO gas sensors require high operating temperature (up to 400 °C) in sensing applications, which causes high power consumption and thus leads to instability during long-term gas detection. Therefore, more thoughtful strategies were developed to enhance their sensitivity and selectivity with lower power consumption [107,121,122,123,124,125,126,127].

**Figure 27:**
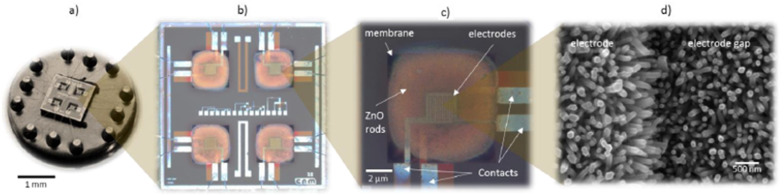
Photograph of the micromachined gas sensor device mounted on a standard TO-8 package (**a**). Optical microscope imaging of the four micromachined sensors in the array and (**b**) detailed view of a single sensor; (**c**) images were obtained using polarized filters to provide better contrast among the ZnO rods (orange), the electrodes (yellow), SiO_2_/Si_3_N_4_ micromachined membrane (black) and SiO_2_/Si bulk (grey). (**d**) SEM imaging of the aerosol-assisted chemical vapour deposited ZnO rods into the micromachined platform, showing the edge of an electrode [121]. Copyright (2018), with permission from Elsevier.

In 2020, Krishnakumar et al. investigated ZnO-based sensors for hydrogen sensing at a high operating temperature of 400 °C with fast response and recovery time (5 s/7 s) [107]. The sensor exhibited a relatively low sensing response (R_a_/R_g_ equals 10 at 2000 ppm H_2_). Earlier in 2018, Vallejos et al. synthesized ZnO rods-based hydrogen sensors via chemical vapour deposition [121], as shown in Figure 27. Due to the large effective surface, the ZnO rods exhibited better sensitivity for 100 ppm hydrogen concentration and at a relative lower temperature of 350 °C. The low detection limit was determined to be 10–90 ppm.

**Figure 28:**
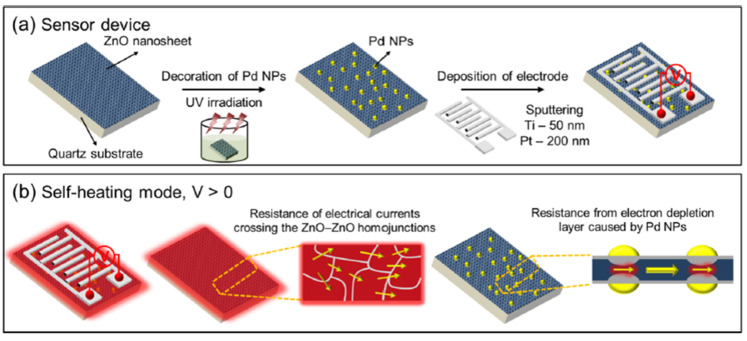
(**a**) Schematic of sensor fabrication and (**b**) operation in self-heating mode [122]. Copyright (2021), with permission from Elsevier.

**Figure 29:**
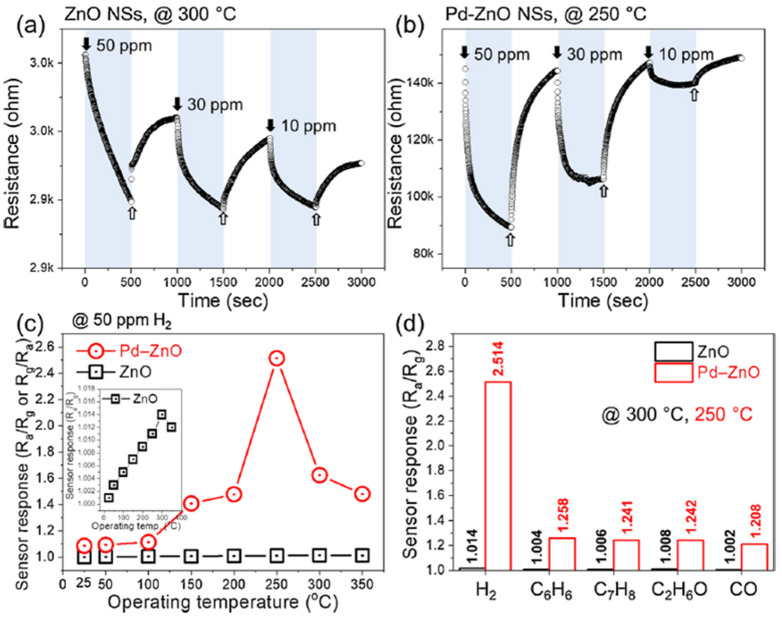
Dynamic resistance curves of (**a**) pristine and (**b**) Pd-decorated ZnO nanosheets to H_2_ gas. (**c**) The response of pristine and Pd-decorated ZnO nanosheets to 50 ppm of H_2_ gas at different sensing temperatures. The inset shows the response of a pristine sensor to H_2_ gas and (**d**) selectivity patterns of ZnO and Pd-decorated ZnO nanosheet at optimal temperatures [122]. Copyright (2021), with permission from Elsevier.

Kim et al. recently investigated two-dimensional (2D) ZnO nanosheets decorated with pristine and Pd for different sensing modes to lower the power consumptions in 2021 [122]. The ZnO nanosheets with an ultrathin thickness of 1 nm were synthesized using a facile hydrothermal method and decorated with Pd NPs using the UV reduction method, as shown in Figure 28a,b. Using the thin ZnO film nanostructure, the hydrogen gas sensors exhibited high performance with reduced power consumption. When sensors based on the pristine and Pd-decorated ZnO nanosheets were exposed in hydrogen, the pristine sensor showed no significant differences with increasing H_2_ concentration at the optimal working temperature of 300 °C, as shown in Figure 29a. On the other hand, at 250 °C, the Pd-decorated ZnO nanosheets exhibited higher response (R_a_/R_g_ = 2.5) to 50 ppm H_2_, and better selectivity towards H_2_ compared to other gases like CO, $C_{6}H_{6}$, $C_{7}H_{8}$, and $C_{2}H_{6}O$. The low detection limit of the Pd-decorated ZnO ultra-thin film sensor can reach 0.5 ppm (see Figure 29b–d). In self-heating mode, the Pd-decorated ZnO nanosheets exhibited relatively lower responses and poor selectivity among the test gases at the optimal applied voltage of 1 V to 20 V. In their investigations [122], the Pd-decorated ZnO thin-film sensor exhibited p-type behaviour at 25 °C, increasing resistance upon introducing H_2_ gas. In the range of 100–350 °C, the Pd-decorated ZnO nanosheets exhibited n-type behaviour, in which the resistance decreased during the introduction of H_2_ gas. Similarly, Kim et al. also developed a Pd-decorated-ZnO based gas sensor for H_2_ detection [123]. The sensing part was Pd-loaded ZnO nanofibers synthesized by a facile electrospinning technique. It was found that the Pd-loaded (0.6 wt%) ZnO nanofibers exhibited a high response of R_a_/R_g_ = 74.7 at 350 °C for a low concentration of hydrogen at 100 ppb.

The sensitivity of Pt/ZnO Schottky diode-based hydrogen sensors is reduced when switching to humid ambient $H_{2}$ conditions in the presence of water due to hydroxyl groups on the Pt surface. Consequently, Jang et al. [124] spin coated the PMMA on Pt/ZnO Schottky diode sensors as a protective layer to enhance hydrogen sensing performance. Due to its hydrophobicity, the PMMA layer can effectively serve as a moisture barrier and selectively filter the water vapour during hydrogen sensing. The hydrogen sensitivity of PMMA-coated diode sensors could be recovered to up to 805% under humid $H_{2}$ ambient conditions at room temperature. Under dry and humid conditions, ZnO diodes exhibited relatively fast and stable on/off switching with good repeatability in each cycle.

Nakate et al. in 2019 investigated an H_2_ sensor-based on hollow ZnO particles that exhibited remarkable sensitivity and selectivity towards hydrogen gas by heat treatment to polystyrene@ZnO core-shell microstructure [125]. At an optimized temperature of 225 °C, the sensor showed the lowest $H_{2}$ detection limit of 2 ppm with a response of 7%.

Other metal oxide semiconductors like ${\mathrm{TiO}}_{2}$ [128,129,130], ${\mathrm{WO}}_{3}$ [131,132] were also investigated intensively for hydrogen sensing applications. Krško et al. in 2017 fabricated a flexible ${\mathrm{TiO}}_{2}$-based gas sensor by depositing ${\mathrm{TiO}}_{2}$ on a 38 μm thick polyimide foil [128]. The sensor is highly sensitive at room temperature with the achieved response (R_a_/R_g_) of about $10^{4}$ for 1000 ppm H_2_ in synthetic air. Its low detection level was found to be about 30 ppm. The authors explained that such high sensitivity is due to the meager grain size (about 10 nm), which is comparable to the width of the depletion layer. Moreover, the ${\mathrm{TiO}}_{2}$ films exhibited good adhesion to the polyimide foil. The sensitivity values did not decrease after bending the sensor 1000 times, and no damage was evident. Chen et al. in 2016 in [129] presented a porous Pt-${\;\mathrm{TiO}}_{2}$ nanocomposite (${\mathrm{TiO}}_{2}$: P25, 80% anatase and 20% rutile; Pt power: particle sizes of ~10 nm) ceramics, synthesized by applying a pressure of about 10 MPa and sintering at 450 °C. Such sensors exhibited a high hydrogen sensitivity of about 6000 for 1000 ppm H_2_ balanced in $N_{2}$ and a fast response/recovery times of 10/20 s at room temperature. Because hydrogen molecules can easily penetrate the porous nanoparticles, this leads to increased hydrogen reactivity. Furthermore, the Pt aggregates in the nanocomposites improved the dissociation of hydrogen molecules and helped the migration of hydrogen atoms to migrate onto surfaces of ${\mathrm{TiO}}_{2}$ nanoparticles by spill-over process [130].

Tungsten trioxide (${\mathrm{WO}}_{3}$) exhibits chemochromic properties resulting from a change in the metal ions’ oxidation state, usually transparent crystalline oxide. With the decoration of catalytic metal NPs such as Pd or Pt, the ${\mathrm{WO}}_{3}$ enable a reversible colouration process upon exposure to hydrogen. Partially, the reduced oxide turns blue or black. The chemical reactions can be described as [132]:

(21) $${\mathrm{WO}}_{3}+{\mathrm{xH}}^{+}+{\mathrm{xe}}^{-}\;\to {\;H}_{x}{\mathrm{WO}}_{3}$$

(22) $$H_{x}{\mathrm{WO}}_{3}+\frac{x}{4}O_{2}\;\to {\mathrm{WO}}_{3}+\frac{1}{2}{\mathrm{xH}}_{2}O\;$$

**Figure 30:**
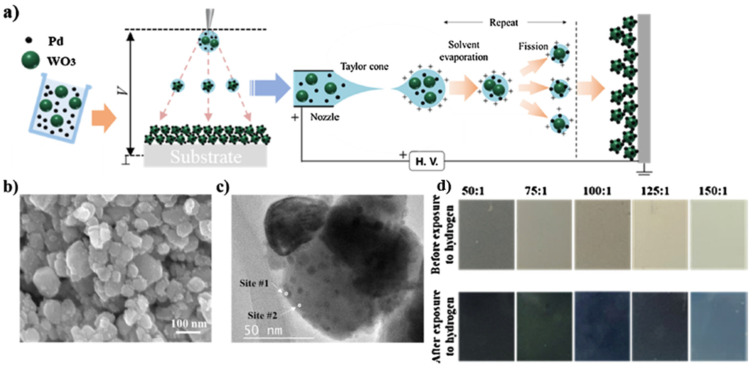
(**a**) Schematic of electrostatic spray deposition (ESD) process for fabricating ${\mathrm{WO}}_{3}$-Pd sensor, (**b**) SEM image of deposited ${\mathrm{WO}}_{3}$-Pd layer with a ${\mathrm{WO}}_{3}$:Pd ratio of 75:1, (**c**) TEM image of ${\mathrm{WO}}_{3}$-Pd, and (**d**) optical images of sensors with ${\mathrm{WO}}_{3}$:Pd ratios of 50:1, 75:1, 100:1, 125:1 and 150:1 before and after exposure to 1% hydrogen gas [131]. Copyright (2021), with permission from Elsevier.

Lee et al. in 2021 developed a cost-effective chemochromic gas sensor based on Pd and ${\mathrm{WO}}_{3}$ nanoparticles for hydrogen detection [131]. The sensing materials were fabricated via electrostatic spray deposition (ESD) of a solution of Pd and ${\mathrm{WO}}_{3}$ NPs. During the processing, fine droplets of the solution were broken down into sub-micron scales via electric forces, ensuring the uniform deposition of sub-micron droplets on a target substrate. Moreover, the ESD process enabled the sub-micro-scale structure with well-defined porosity and a high aspect ratio, facilitating gas diffusion. The Pd catalyst reduced the activation energy of hydrogen adsorption and allowed hydrogen adsorption on the surface of ${\mathrm{WO}}_{3}$ via the spill-over effect [133,134]. The adsorption of hydrogen ions and electrons changed the charge state of tungsten ions. Upon the reduction of $W^{6+}$ to $W^{5+}$, the chemochromic colour of ${\mathrm{WO}}_{3}$ changes from pale green ($W^{6+}$) to deep blue ($W^{5+}$) [135], as shown Figure 30. On these sensing layers, the chemochromic property of the ${\mathrm{WO}}_{3}$-Pd sensor is determined by the surface chemical reaction of the adsorbed gas species. For the quantitative investigation of the chemochromic properties, the spectrometer was employed to measure the light beam’s colour difference (dE) injected into the sensor and reflected subsequently. As a result, the optimized sensor was found to have a ${\mathrm{WO}}_{3}$:Pd molar ratio of 125:1 and a thickness of 5 μm. This chemochromic sensor demonstrated a rapid sensing response, with a colour change occurring within 15 s at room temperature. The sensor exhibited a deep blue colour in the presence of hydrogen, with the dE value of 18.5 at 1% hydrogen concentration. The lowest detection limit of the sensor could be achieved at 0.2% concentration, and the reversibility in colouration and recovery was demonstrated over 20 times with repeated exposures to hydrogen and air.

**Figure 31:**
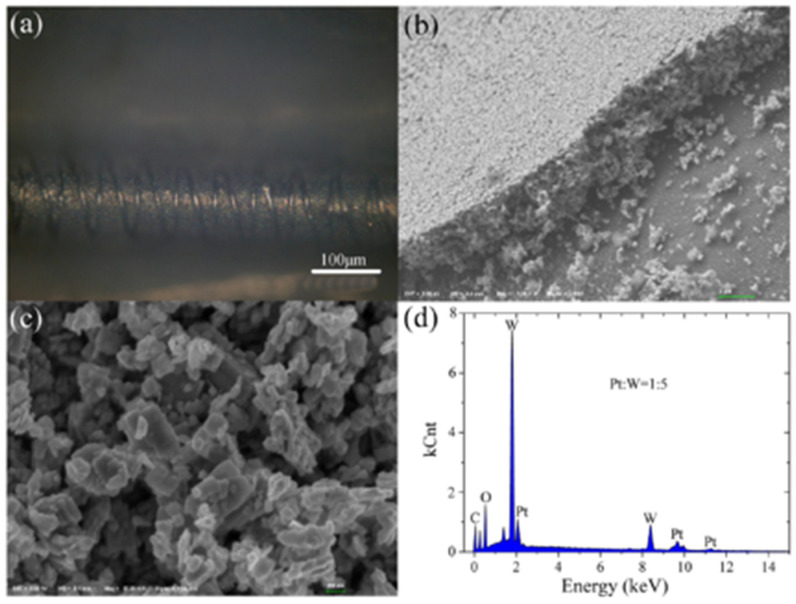
(**a**) Morphology of double spiral microstructure after depositing Pt-WO_3_ film. (**b**) The cross-section diagram of Pt-WO_3_ film. (**c**) The SEM picture of nanostructure of Pt-WO_3_. (**d**) The energy-dispersive X-ray spectrometer (EDS) pattern of Pt-WO_3_ film [136]. Copyright (2017), with permission from OPTICA Publishing Group.

Zhou et al. investigated an optical fibre Bragg grating (FBG) hydrogen sensor based on Pt-loaded WO_3_ in 2017, as shown in Figure 31 [136]. The grating structure was processed using a fermto second (fs) laser with a wavelength of 780 nm, a pulse duration of 180 fs and a repetition rate of 1 kHz. Unlike the single spiral grating structure, this sensor exhibited a better sensing performance through processing a double spiral grating structure in FBG. The sensing mechanism was based on thermal optic changes caused by the reaction of hydrogen gas and Pt-WO_3_ [137]. The wavelength shift induced by the temperature variation correlates with the thermo-optic effect, thermo-expansion and elasto-optical effects on the optical fibre [138]:

(23) $$\frac{\Delta \lambda_{B}}{\lambda_{B}}=\left[\;\zeta +\;\alpha \;+\left(1+p_{e}\right)\times \left(\alpha_{\mathrm{film}}-\;\alpha \right)\right]\Delta T$$

where $p_{e}$ is the elasto-optical coefficient, and α and ζ refer to thermal expansion and thermo-optic coefficients of fibre. Additionally, $\alpha_{\mathrm{film}}$ is the thermal expansion of film, $\Delta \lambda_{B}$ is the Bragg wavelength, and ΔT is the temperature change.

As reported in [139], the double spiral grating FBG structure sensor displayed 2–4 times improvement in H_2_ sensing compared to that based on a single spiral grating FBG structure. At the optimized mole ratio of Pt:WO_3_ 1:5, the sensor exhibited a higher sensitivity (522 pm% (*v*/*v*)) and faster response (15 s) at room temperature towards 1% H_2_ concentration. The main reason for this improvement was the large reaction surface created by the double spiral grating and the inhomogeneous stress distribution caused by fs ablation, which increased the wavelength shift and further improved the sensor’s sensitivity.

Another promising strategy to enhance the hydrogen sensing properties of metal oxide-based sensors is to form a heterojunction with the “backbone” materials, such as SnO_2_, TiO_2_ or ZnO, due to their stability, low cost, and superior electrical properties. As a heterojunction is formed, a renewed charge distribution occurs accordingly, creating electron depletion in one material and electron accumulation in another. This redistribution dramatically changes the total number of charge carriers and leads to measurable improvements in the sensitivity and selectivity of the sensor.

**Figure 32:**
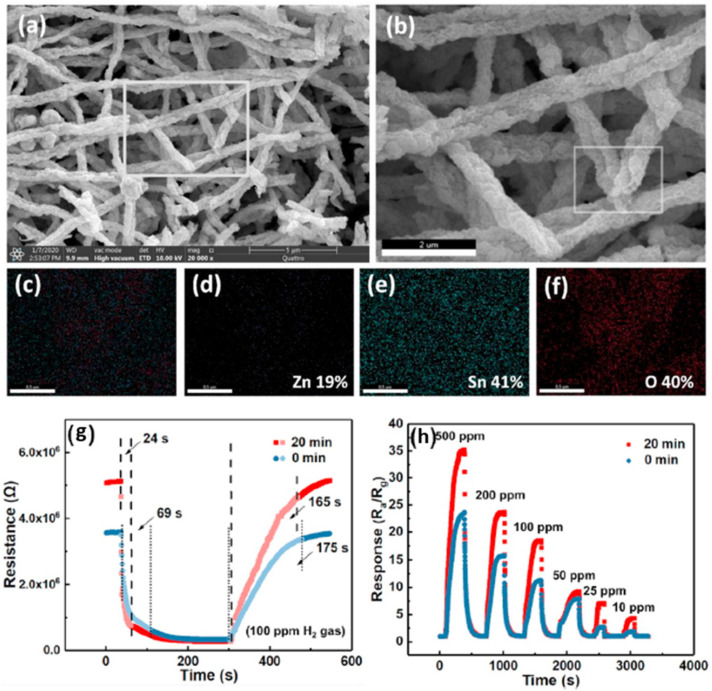
(**a**,**b**) SEM images of ZnO–SnO_2_ nanofibers, (**c**–**f**) images of area scan by EDS including the element content of Zn, Sn, and O. (**g**) Response and recovery at optimum operating temperature 300 °C. (**h**) Response curve with 10 ppm to 500 ppm H_2_ concentrations at an optimum operating temperature [123]. Copyright (2020), with permission from Elsevier.

Hu et al. synthesized ZnO-SnO_2_ heterojunction nanofibers by electrospinning and plasma treatment for hydrogen sensing applications in 2020 [123]. Figure 32a,f show the surface morphology of the ZnO-SnO_2_ nanofibers. For comparison, both plasma treated (optimized to 20 min) and the original samples (no plasma treatment) were exposed to 100 ppm H_2_ concentration. At the optimized working temperature of 300 °C, the plasma-treated sensor exhibited better performance with a sensor response (R_a_/R_g_) of 18, compared to a response of about 10 for the original sensor at 330 °C (the optimized temperature for the sensor without plasma treatment) as shown in Figure 32g,h. Moreover, the response time of the plasma-treated sensor was shorter at 24 s, compared to 69 s for the untreated sensor. This kind of plasma-treated sensor can detect the H_2_ concentration down to 10 ppm. Lee et al. controlled the content of SnO_2_ in their SnO_2_/ZnO heterojunction hydrogen gas sensor and reported in their paper [127] that the sensors with the xSnO_2_-loaded (x = 1) ZnO nanofiber also exhibited a high response (R_a_/R_g_ = 50.1) to 50 ppb hydrogen at 300 °C. Furthermore, this kind of sensor showed an excellent selectivity towards the hydrogen gas sensor.

**Figure 33:**
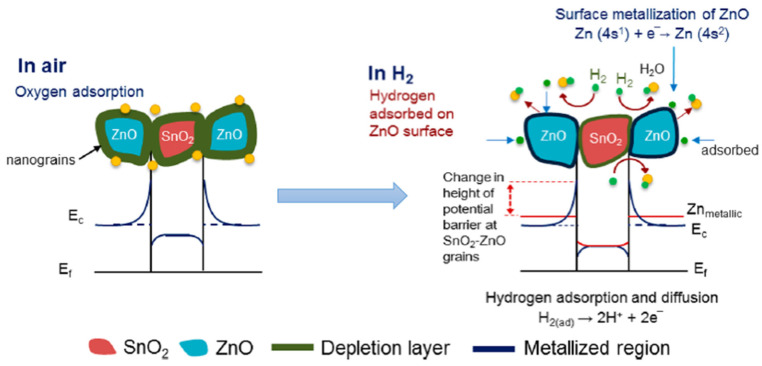
The schematic diagram for explanation of sensing mechanism of ZnO-SnO_2_ heterojunction [140]. Copyright (2015), with permission from American Chemical Society.

The hydrogen sensing mechanism has been illustrated graphically in Figure 33 from previous work in 2015 [140]. When ZnO-SnO_2_ heterojunctions are exposed in the air, the adsorbed oxygen molecules from the air are on ZnO-SnO_2_ grains and dissociated into oxygen ions. In particular, the chemisorption process takes place on vacant oxygen sites, which form a depletion layer in the grains, depleting their thickness, accompanied by an increase in grain resistance. When hydrogen is released, the adsorbed oxygen reacts rapidly with hydrogen. As a result, the depletion layer becomes thinner, and the resistance of grains is reduced accordingly. Meanwhile, ZnO grains are directly reduced to Zn metal, forming a metalized layer on the grain surface. This process significantly affects the potential heterojunction barriers and dramatically improves the range of resistance modulation in the ZnO-SnO_2_ heterojunction.

In 2019, Lee et al. and their team investigated the heterojunction gas sensor based on Pd-functionalized In_2_O_3_-loaded ZnO nanofiber and synthesized by electrospinning and an ultraviolet irradiation method for hydrogen sensing applications [141]. Besides the hydrogen active Pd NPs, the sensor also employed heterojunctions like In_2_O_3_/ZnO, homojunctions like ZnO-ZnO and In_2_O_3_-In_2Tablw_O_3_, and the Schottky junction Pd/ZnO, which all contribute to improved gas sensing performance. Sensors with an optimized mole ratio of 0.1 In_2_O_3_ have been reported to exhibit a high response of R_a_/R_g_ = 172 for an ultra-low hydrogen concentration of 50 ppb at 350 °C.

**Figure 34:**
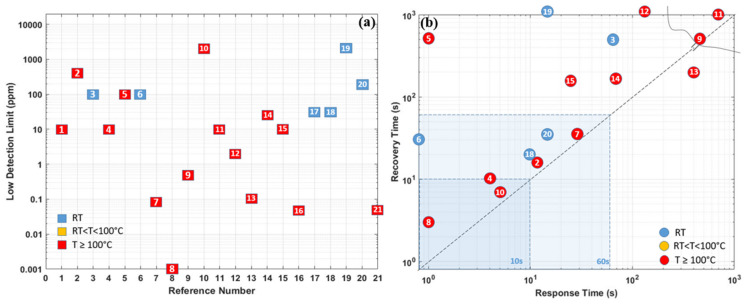
Regarding Table 2, schematic illustrations of the comparison of the reported: (**a**) low detection limits. The x-axis “Reference Number” represents the 1st column“Ref. Nr.“ in Table 2. (**b**) Response time/recovery time under experimental conditions in the literature.

A comparison of the MOS-based hydrogen sensors with their operating temperature, synthesis method, and performance parameters are presented in Table 2. Right after the table, Figure 34 schematically illustrates the temperature, response, and recovery time. Generally, MOS-based hydrogen sensors require a higher ambient operating temperature, which requires high power consumption. However, high temperature can also effectively reduce the weakening of sensor sensitivity by humidity, thus allowing the MOS sensor to reach a detection range of ppb level. On the other hand, the lifetime of the sensor is significantly reduced as high temperatures accelerate the fatigue of the sensing material.

## 4. Graphene and Its Derivatives-Based Hydrogen Gas Sensors

As mentioned in the previous sections, despite several advantages of the Pd based hydrogen gas sensors, there are also disadvantages, for example, high cost for low H_2_ concentrations (less than 1%). The Pd-film sensor has a long response time. For high H_2_ concentrations (more than 2%), the Pd lattice expansion leads to the Pd film buckle or collapse, making the sensor unstable, irreversible, and unreliable [23]. For Pd-nanostructure sensors like nanowires, and nanocomposites, the very low conductance of these discontinuous nanostructures induces a high electrical noise, degrading the sensor response. In contrast, as another promising hydrogen gas sensor, most of the typical commercial MOS-based sensors have cost limitations, such as high power consumption, rare sensitivity in ppb level, limited lifetime, poor selectivity and repeatability, and difficult miniaturization. Therefore, graphene(G) and its modified derivatives have been employed as promising special materials to enhance the sensing performance due to their unique features.

**Figure 35:**
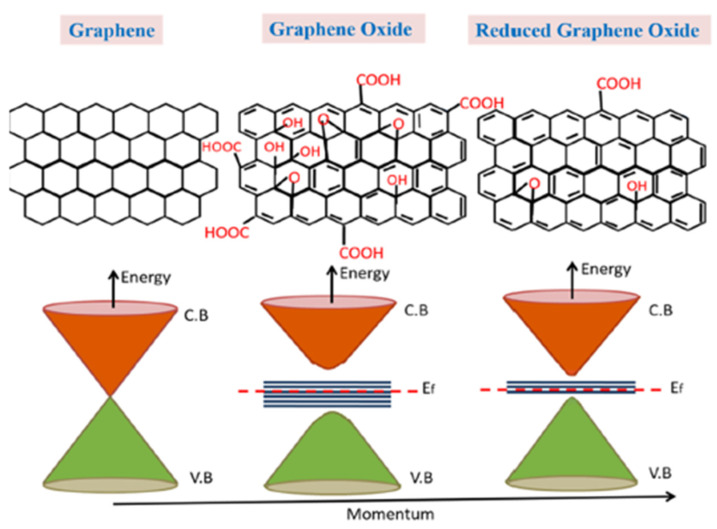
Schematic representation of lattice structure and corresponding energy diagrams of graphene, graphene oxide (GO) and reduced graphene oxide (rGO) [149]. Copyright (2018), with permission from Nature Publisher Group.

Graphene has attracted attention continuously for decades [142]. Graphene has been acknowledged as a novel material for sensor applications due to its high surface-to-volume ratio, specific large surface area, fast electron-transfer kinetics, and remarkable carrier mobility [138,143,144,145,146,147]. Its high carrier mobility ensures the desired low electrical noise, fast response, high sensitivity, and low energy consumption [138,145]. However, the pristine graphene sheets cannot be applied directly to the hydrogen sensor [148] because pure graphene has no bandgap (see illustration in Figure 35) nor functional groups on its surface for gas adsorption, thus presenting a low adsorption behaviour. Therefore, it is suggested that functional graphene or its derivatives are combined with other substances like transition metals, metal oxide semiconductors (MOS), or even polymers in sensing applications.

As one of the derivatives of graphene, graphene oxide (GO) is mainly fabricated by a modified Hummers method via oxidizing from graphite [150], which creates different functional groups and defects (it is much easier to obtain GO using a wet chemical method from graphite as compared to producing pure graphene). These newly gained groups are located on its basal plane and edges: carboxyl (–OOH), epoxy (–O) and hydroxyl (–OH) [151,152]. Since π-bonding is weak due to its low formation energy, numerous disorder-induced localized states can present in the band tail of the π-$\pi^{*}$ gap. With these functional groups, the electric structure of graphene changes, and a bandgap of about 2.2 eV can be achieved [149]. The sensitivity and repeatability of a sensor depend a lot on the amount of oxygen functional groups on the GO’s surface. However, the content of the oxygen functional groups is difficult to control during the synthesis process [153].

Due to the presence of the oxygen functional group, GO is by nature insulating. However, the electrical conductivity can be improved by removing some oxygen functional groups via a controlled reduction process and keeping some oxygen functional groups to limit electron transport. GO can be converted to another graphene derivative, reduced graphene oxide (rGO), by using a chemical reduction method. The bandgap of rGO varying between 1 eV to 1.69 eV depending on the degree of reduction [154,155,156]. The removal of some oxygen groups allows the bandgap to be adjusted during the reduction process, so that the oxygen presenting in rGO can be further engineered. Therefore, rGO behaves like a semi-metal or semiconductor, and its electrical conductivity can be tuned by controlling its oxygen content [155].

### 4.1. Transition Metals/Graphene Combinations

Knowing that graphene lacks active sites, Shao and Hayasaka et al. investigated a crumpled graphene structure-based stretchable gas sensor for hydrogen sensing [157]. The chemical vapour deposition (CVD)-grown monolayer flat graphene is first transferred on a substrate made of elastomer polymer during its fabrication. This substrate has a pre-strain of about 200% in both the *x* and *y*-direction. After the deposition of gold or palladium (Au/Pd) electrodes, it is interesting that the substrate releases pre-strain and crumples up accompanying the transferred graphene. Subsequently, this process increases the contact area, distorts π bonds and strengthens the interaction of the Au/Pd deposited surface with gas molecules. This deposition of Au/Pd electrodes works like an enhancement, and this material-based sensor has a sensitivity of 0.58% at room temperature surrounded by 0.1 vol% hydrogen concentration. It has a better sensitivity than the flat graphene on SiO_2_-based sensor, which has a sensitivity of 0.18%, as reported in [157]. Another derivative to improve the sensitivity of graphene towards hydrogen is the nanoporous graphene oxide (NGO). The work [158] found that the NGO has a large surface, high content of oxygen functional groups (hydroxyl, epoxy, carbonyl, and carboxyl groups) and high electric conductivity. Shaban et al. in [159] fabricated NGO film by a modified Hummer method followed by a spray pyrolysis method for hydrogen sensing application at room temperature. During its characterization, a hydrogen gas flew at 60 SCCM (standard cubic centimetres per minute) for 5 min, the nanoporous sensor has a faster response/recovery time of 100 s/437.2 s with a sensitivity of 16.16% compared to those using pure graphene [157]. However, the selectivity of this kind of NGO gas sensor is relatively low, since the sensitivity of CO_2_ sensing surrounded by a particular concentration of released gas is higher than its sensitivity towards hydrogen [157]. Compared with graphene and its derivatives, rGO has been proved to have a better response by Wang et al. [160]. The rGO-based hydrogen gas sensor is fabricated on a micro-hotplate by dielectrophoresis (DEP) and has a good sensitivity of around 6% with a relatively fast response and recovery time of 11 s/36 s for 200 ppm hydrogen at room temperature. However, when the working temperature increases to 300 °C, the sensitivity of the rGO gas sensor could approach a higher value of about 17% towards hydrogen, and the response time is around 1 s, much faster than at room temperature.

**Figure 36:**
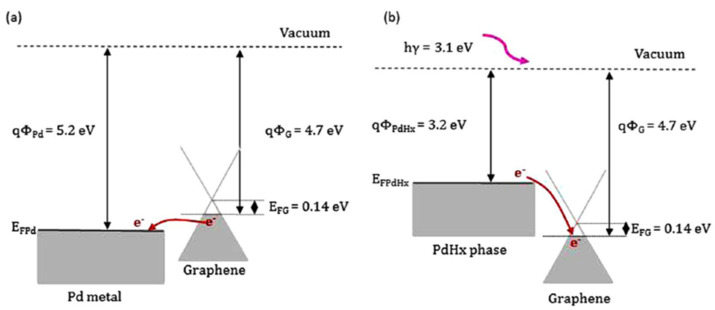
Energy band diagrams (**a**) for Pd decorated graphene and (**b**) for PdH_x_ and graphene [162]. Copyright (2019), with permission from Nature Publisher Group.

Since Pd nanoparticles have superior hydrogen solubility at room temperature [144,161], decoration using Pd nanoparticles can induce a change of work function of Pd with low noise and low energy consumption, thus changing the electron mobility of the Pd nanoparticles-decorated sensor [26,162,163,164,165]. In [162], Tang et al. developed a hydrogen detection gas sensor based on Pd nanoparticles-decorated graphene at room temperature, where Pd nanoparticles act as reaction sites for H_2_ absorption and graphene as an electron pathway. Without releasing hydrogen, the work function of the Pd nanoparticle is larger than that of the CVD fabricated graphene [166,167], as shown in Figure 36a. This difference led to electron transfer from graphene to Pd nanoparticles. Increased hole density in graphene improves the conductivity and reduces the resistance of graphene [168]. When the gas sensor is exposed to hydrogen, the work function of the hydride PdH_x_ formed has a lower work function of 3.2 eV than that of Pd [30], leading to a decrease in the hole density in graphene and hence an increase in its resistance, as shown in Figure 36b. This sensor has an extended response and recovery time of 3 min and 9 min, respectively, with a sensitivity of 5.88%. In order to improve the sensing response, Yokoyama et al. developed hydrogen sensors with suspended graphene films functionalized with Pd nanoparticles [164], which introduced self-heating changes in sensor performance. The suspended graphene film also avoids heat transfer to the substrate. With a low power consumption per unit graphene width of 0.93 mW/μm, graphene can create a working temperature of up to 180 °C.

The sensor achieves a fast response to hydrogen with a response time of 15 s at 100 ppm hydrogen concentration. For the same purpose, Sharma et al. functionalized graphene with Pd–Ag alloy nanoparticles in their dual-gate field-effect transistor (FET) [165]. The study [169] showed that the Pd–Ag alloy nanoparticles could improve mechanical stability, hardness, chemical inertness and hydrogen permeability. The working temperature can be tuned between 25 and 254.5 °C, employing an external micro heater Pt. At 245 °C, the sensor has a rapid response time of 16 s and recovery time of 14 s at 1000 ppm hydrogen concentration, and a low detection limit at around 1 ppm is observed.

**Figure 37:**
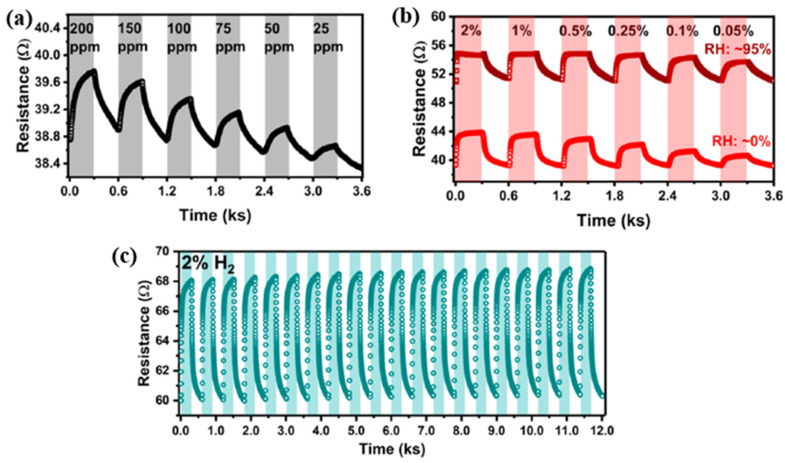
(**a**) Sensitivity of Pd-CGB sensor at ppm levels of H_2_ concentration from 25 to 200 ppm. (**b**) Effect of humidity on the sensor performance at two relative humidity (RH) levels: RH~0% and RH~95%. (**c**) Repeatability test of Pd-CGB sensor at 2% H_2_ for 20 cycles [170]. Copyright (2020), with permission from American Chemical Society.

With the application of reduced graphene oxide (rGO), Mohammadi et al. in [170] investigated Pd-decorated crumpled rGO balls for hydrogen detection at room temperature by using a flame technology [171]. A rapid, continuous high-temperature reducing jet (HTRJ) process produced the nanocomposites. Their three-dimensional structure facilitated gas sensing due to the highly accessible surface area, further improving the electron transfer between the material and the electrode surface [172,173]. In their work, the detection range of H_2_ concentration could be extended between 25 ppm and 20,000 ppm. In addition, this paper also investigated the effect of humidity on the sensor, as illustrated in Figure 37b. Humid air shifts the baseline of the sensing signals from ~38 Ω at ~0% RH (under dry condition) to ~51Ω at ~95% RH. Furthermore, Figure 37c exhibits the repeatability of the Pd-crumpled reduced graphene (CGB)-based gas sensor. After 20 cycles of testing with 2% H_2_ concentration at room temperature, the sensor presented a stable sensing performance with a response value of 14.8%, a response time of 73 s, and a recovery time of 126 s.

**Figure 38:**
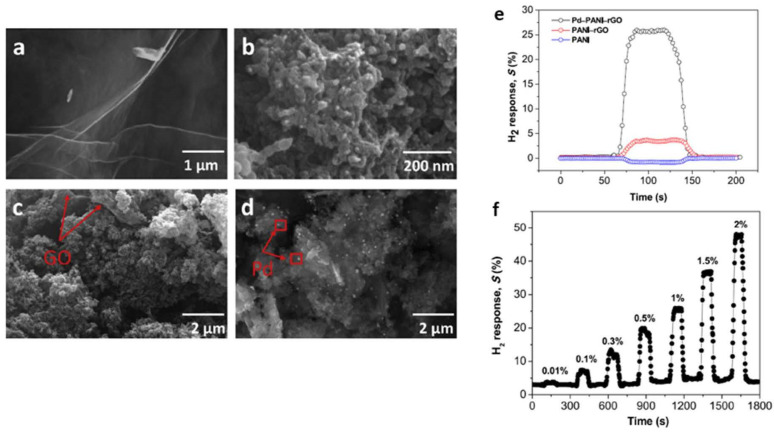
Scanning electron microscope (SEM) images of (**a**) rGO, (**b**) PANI (polyaniline), (**c**) PANI-rGO (reduced graphene oxide) and (**d**) Pd-PANI-rGO. (**e**) Gas sensing response of the PANI, PANI-rGO and Pd-PANI-rGO thin films exposed to 1% hydrogen concentration at room temperature. (**f**) Gas sensing response of the Pd-PANI-rGO based sensor to different concentrations of hydrogen at room temperature [176]. Copyright (2016), with permission from Elsevier.

In recent years, since sensitive polymers have attracted interest in gas sensing research, PANI-based sensors have also been expanded to hydrogen detection by combining PANI with other nanomaterials [174,175]. Zou et al. developed a gas sensor with PANI-rGO composite doped with Pd nanoparticles [176]. Figure 38a–f present images of material blending from rGO to Pd-PANI-rGO. As shown in Figure 38, the pure PANI forms short rod-like agglomerates with an average diameter of about 80 nm before being compounded with rGO in Figure 38c. After that, 1 mg of K_2_PdCl_6_ solution (density: 0.2 g/L) was first added, after 2 h, 1 mL of NaBH_4_ solution (density: 1 g/L) was added for the trying process of the mixture. The form of Pd-PANI-rGO composites has been shown in Figure 38d. In principle, similar to rGO, the PANI-rGO exhibits p-type behaviour [177], with electron transfer from PANI-rGO to Pd resulting in a modified level charge-neutrality at the Pd-PANI-rGO interface [178]. Due to the lower chemisorption work function of PdH_x_ formed by the release of hydrogen than that of PANI-rGO, electron transfers from PANI-rGO to PdH_x_ decrease and the resistance of the PANI-rGO surface increases. Figure 38e demonstrates the sensing response of PANI, PANI-rGO, and Pd-PANI-rGO in an environment with 1% H_2_ concentration at room temperature, which shows that Pd-PANI-rGO is significantly more sensitive towards hydrogen (~25%) due to its enhanced gas diffusion capability and the high H_2_ absorption capability of Pd. In Figure 38f, the hydrogen sensing response of the Pd-PANI-rGO based sensor was tested in various H_2_ concentrations at room temperature. A low detection limit of 0.01% H_2_ concentration was reported. At an H_2_ concentration of 1%, the response time and recovery time of the Pd-PANI-rGO based sensor were 20 s and 50 s, respectively. In their study, Zou et al. compared the sensing performance towards different gases (CH_3_OH, CO_2_, H_2_S) and further demonstrated the remarkable selectivity of the Pd-PANI-rGO sensor towards hydrogen.

**Figure 39:**
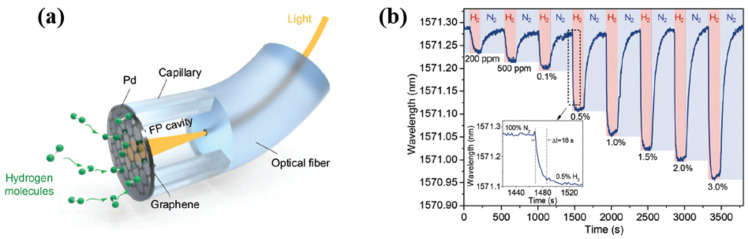
(**a**) Schematic of a fibre-optic H_2_-sensor with Pd-decorated MLG film, and (**b**) temporal response of the Pd/MLG sensor with a 5.6 nm Pd film for various H_2_ concentrations [163]. Copyright (2019), with permission from Royal Society of Chemistry.

Despite research investigating the chemi-resistive graphene-based hydrogen gas sensor, which achieved promising sensing performance, there have also been studies focusing on a Pd-decorated graphene-based optical gas sensor for hydrogen sensing application. For instance, Ma et al. in [163] demonstrated a miniature fibre-optic hydrogen sensor with Pd-decorated multilayer graphene (MLG), synthesized by the CVD method. This Pd/MLG film covered the air cavity at the optical fibre tip, forming a flexible Fabry–Pérot (FP) interferometer, as shown in Figure 39a. During H_2_ Adsorption, the expanded Pd lattice causes stretching and defects in the Pd/MLG film, reducing the length of the FP cavity and leading to a wavelength shift in the reflection spectrum within the cavity. Figure 39b presents the response of the MLG coated with a 5.6 nm-thick Pd film and exposed to a range of hydrogen concentrations up to 3%. A low detection limit of ~200 ppm can be achieved, and a wavelength shift of ~50 pm/ppm is induced. At an H_2_ concentration of 0.5%, the sensor can respond to H_2_ within ~18 s.

**Figure 40:**
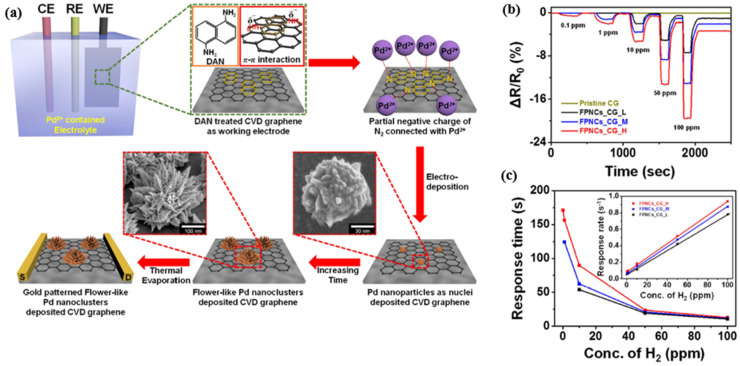
(**a**) Illustrative diagram for the fabrication steps of flower-like Pd nanoclusters (FPNCs) on chemical vapour deposition (CVD) graphene electrode. (**b**) Normalized resistance changes upon sequential exposure to H_2_ gas of various concentrations (0.1 to 100 ppm) at room temperature, and (**c**) response time and rate (inset) vary with H_2_ concentration [179]. Copyright (2015), with permission from Nature Publisher Group.

For further enhancement of the gas sensing performance, Shin et al. developed graphene decorated with flower-like Pd nanoclusters (FPNC) by treating it with 1.5-diaminonaphene (DAN) [179], as shown in Figure 40. Due to expanded reactive sites, the gas sensor achieved an ultra-low hydrogen detection limit of 0.1 ppm at room temperature. The decorated graphene was transferred to a flexible polyethylene naphthalate (PEN) film, used as the sensor substrate. The gas sensor was, therefore, mechanically stable and stretchable. Even after 100 bending cycles, the sensing response decreased by only 2%.

**Figure 41:**
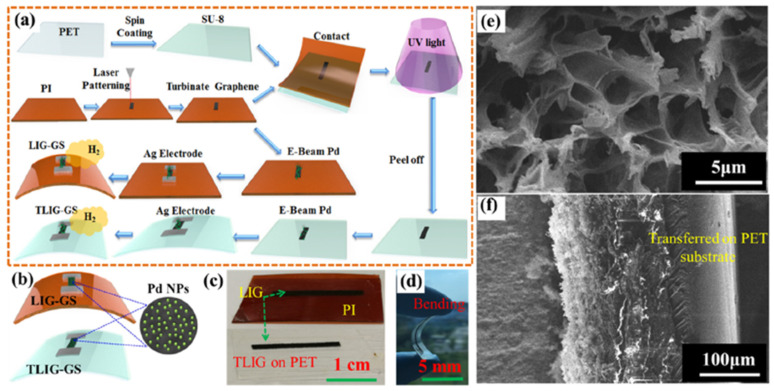
(**a**) The fabrication process of the laser-induced graphene gas sensor (LIG-GS) and transferred LIG-gas sensor (TLIG-GS). (**b**) Schematic of Pd NPs on LIG-GS and TLIG-GS. (**c**) Transferred line pattern of 3D porous LIG. (**d**) Image of bendable TLIG-GS. (**e**) Morphology of TLIG and (**f**) cross-sectional image of TLIG [180]. Copyright (2019), with permission from American Chemical Society.

In a similar effort to obtain a flexible gas sensor, Zhu et al. developed a biomimetic gas sensor based on 3D porous laser-induced graphene (LIG) decorated with Pd nanoparticles for hydrogen sensing at room temperature, inspired by the turbinate structure of the dogs’ olfaction system [180]. Figure 41a shows the synthesis process of a LIG-gas sensor (LIG-GS) on PI substrate and a transferred LIG-gas sensor (TLIG-GS) on PET substrate. Pd nanoparticles were decorated on the sensing surface using an e-beam evaporation process (Figure 41b). As illustrated, LIG-GS was decorated with an e-beam evaporation process directly after the formation of the turbinate graphene, but the TLIG-GS was treated with UV light exposure before the LIG microstructure was transferred onto the PET substrate. Figure 41e shows an SEM image of the 3D porous structure of the LIG, and Figure 41f presents a cross-sectional image of TLIG with a PET substrate. As reported, with the decorated Pd nanoparticles, the sensitivity of LIG-GS was on average 20% higher than that of TLIG-GS. At 1% H_2_ concentration, the sensing response of LIG-GS was ~3.2%, with a response time of ~6 min and a recovery time over 20 min.

**Figure 42:**
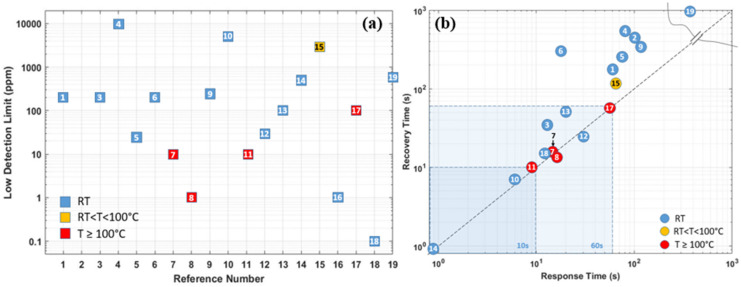
Regarding Table 3, schematic illustrations of the comparison of the reported: (**a**) low detection limits. The x-axis “Reference Number” represents the 1st column“Ref. Nr.“ in Table 3. (**b**) Response time/recovery time under experimental conditions in literature.

As the summary of this section, current research based on graphene and its derivatives or their combinations with transition metal nanoparticles is listed and compared in detail in Table 3. As shown in the table, the graphene and its derivatives-based sensors operate mainly at room temperature, which allows for power consumption. Even sensors operating at high temperatures require a much lower power consumption than that of the transition metal-based hydrogen gas sensors in Table 1 since their maximal operating temperature is below 260 °C [165]. That difference can be observed more clearly by comparing the sensing combinations of both graphene and MOS materials in the following section. Secondly, due to the excellent chemical and physical properties, the graphene (e.g., GO, rGO)-based sensors have been proved experimentally by exhibiting a better electrical conductivity, which leads to fast sensor response and equal response/recovery time, as shown in Figure 42.

### 4.2. Metal Oxide Semiconductor (MOS)/Graphene Combinations

Besides the hydrogen sensors based on transition metal-doped graphene and its derivatives, the combination of graphene (e.g., GO, rGO) with doped MOS particles offers other new possibilities to improve hydrogen detection in gas sensing applications [188,189,190,191,192,193,194,195,196,197,198,199,200,201,202,203,204,205,206,207,208,209]. Many work groups investigated the combination of graphene with n-type MOS such as ZnO [188], SnO_2_ [197,199], WO_3_ [208,209] and p-type MOSs like NiO [196] in combinations with graphene oxide [189] and reduced graphene oxide. These enhancements can significantly improve the sensitivity and selectivity while reducing operating temperature [190,191,192,193,194,195] due to the existence of the functional groups and defects mentioned earlier. Because of the p-type property of graphene and its derivatives, the combinations with MOS x essentially forms a heterojunction, and thus they exhibit heterojunction charge transfer properties during gas absorption and desorption processes. For better selectivity towards the hydrogen, the deposition of transition metal nano components (e.g., Pd, Pt, Au, Ag, and Ni) has been used to functionalize further MOS/graphene gas sensors, and significant improvements have been achieved as in the results of [192,193,197,198,206,208,209].

**Figure 43:**
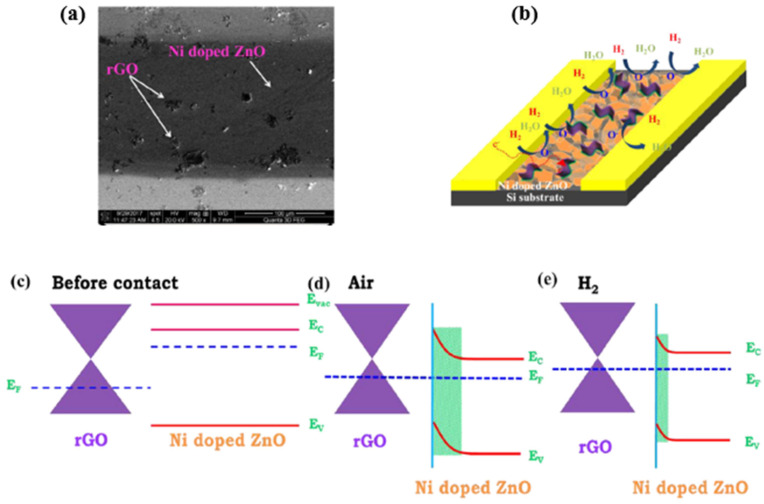
(**a**) SEM image of Ni-doped ZnO nanostructure and 0.75 wt% rGO loaded ZnO. (**b**) Schematic diagram of the gas sensing mechanism in the presence of hydrogen, (**c**–**e**) The band diagram illustration of rGO/ZnO heterojunction before contact and after contact in air and hydrogen) [198]. Copyright (2018), with permission from American Chemical Society.

Among the semiconductor metal oxide, ZnO nanostructures are widely used to functionalize graphene [188], graphene oxide [189] and reduced graphene oxide [191,192], aiming for a better gas sensing performance. Such combinations can improve the sensitivity [188,189,190,191,192] and reduce the working temperature [189,190]. For instance, Galstyan et al. reported in [188] that the ZnO/rGO composites responded 40–50% better to H_2_ at temperature 200 °C compared to pure ZnO sensors. As a further improvement, the transition metals like Pd, Pt, Ag were deposited on the ZnO/rGO gas sensors [192,193,197]. Innovatively, Ni-doped ZnO sensors decorated with reduced graphene oxide (rGO) for hydrogen sensing at 150 °C were investigated by Bhati et al. in [198]. As shown in Figure 43a, the Ni-doped ZnO nanoparticles were synthesized using the RF sputtering method, while rGO was synthesized using Hummer’s method. The purpose of Ni-doping is to create more active sites for the chemisorption of oxygen, thereby enhancing the sensing response at lower operation temperatures [210]. Since rGO (p-Type) and ZnO (n-Type) have different MOS types, a Schottky heterojunction is formed at the rGO/ZnO interface. As illustrated in Figure 43c, rGO has a more extensive work function than Ni-doping ZnO. Since rGO has a smaller bandgap, which makes it behave like a metal, the contact with ZnO generates a Schottky barrier at the heterojunction [200,201] due to the electron transfer from ZnO to rGO at the interface. As a result, it forms a thick depletion layer between ZnO and rGO, accompanied by a decrease in total carrier concentration and an increase in resistance. When these heterojunctions are exposed to the air, electrons are extracted from the conduction band to form oxygen ions adsorbed on the sensor surface, thus increasing the thickness of the depletion layer at the heterojunction interface, which also leads to the increase of the Schottky barrier, as shown in Figure 43d. As hydrogen is filled into the test environment, the hydrogen molecules react with the oxygen ions adsorbed on the sensor surface, and release electrons back into the heterojunction (Figure 43b), which causes the Schottky barrier to decrease, and the depletion layer to become thinner. As a consequence, the total carrier concentration rises, and the resistance falls.

**Figure 44:**
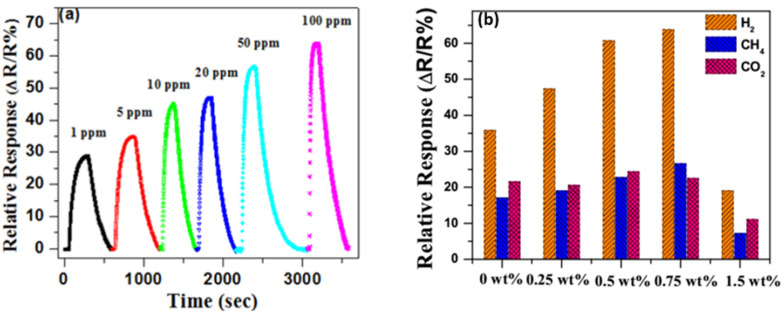
(**a**) Relative response of a 0.75 wt% rGO loaded sensor at an operating temperature of 150 °C with increasing gas concentration from 1 ppm to 100 ppm. (**b**) Selectivity histograms for pristine and rGO-loaded sensors with Ni-doped ZnO nanostructures [198]. Copyright (2018), with permission from American Chemical Society.

As shown in Figure 44a, this heterojunction-based hydrogen gas sensor has a low detection limit of 1 ppm with a relative response of about 30% at the test temperature (150 °C), for a 0.75% wt% rGO-loaded sensor and a response time as fast as 28 s. This heterojunction-based hydrogen gas sensor has, surprisingly, a lower detection limit of 1 ppm with a relative response of about 30% at test temperature for 0.75% wt% rGO loaded sensor and fast response time 28 s, as shown in Figure 44a. Moreover, the sensor has also better selectivity for hydrogen compared to pure ZnO [109]. The optimal rGO concentration was found at 0.75 wt% H_2_ concentration, as shown in Figure 44b.

**Figure 45:**
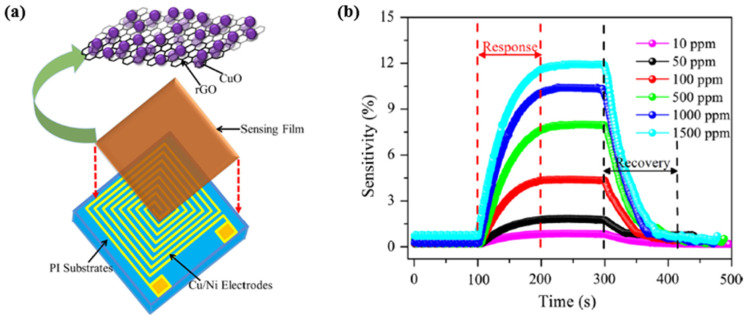
(**a**) Structure illustration of CuO-rGO-CuO hydrogen sensor, and (**b**) response–recovery curves for various concentrations of hydrogen [202]. Copyright (2017), with permission from Springer.

Zhang et al. investigated a 2D material for a hydrogen sensor functionalized with copper oxide (CuO) nanorods deposited on the top and bottom surfaces of rGO, forming a sandwich nanostructure as shown in Figure 45a [202]. CuO is a p-type semiconductor with a bandgap of about 1.2–1.9 eV and is reported in many gas sensor applications [203,211]. The reduced graphene oxide layer provided inherent chemical defects and functional groups, while CuO nanorods can efficiently prevent rGO nanosheets’ aggregation and generate more active sites. This CuO-rGO-CuO hybrid film sensor exhibits excellent response with a low detection limit and high sensitivity at room temperature, as Figure 45b demonstrates. At various hydrogen concentrations from 10 to 1500 ppm, the response time and recovery time were observed to be less than 80 and 60 s, respectively, and the sensitivity increased from 1% (10 ppm) to 12% (1500 ppm).

Peng et al. in [206] compared two hydrogen sensors based on Pd/rGO and Pd/SnO_2_/rGO. The Pd/SnO_2_/rGO nanocomposites-based sensor exhibited higher sensitivity and faster response to H_2_ gas compared to the Pd/rGO based sensor. Over a range of H_2_ concentrations from 100 ppm to 10,000 ppm, the Pd/SnO2/rGO-based sensor has an approximate 50% advantage in response and is about 10% faster in response time and recovery time. Similarly, Dhall et al. investigated Pd-SnO_2_ nanocomposites on a graphene surface in [197] and reported that the sensing response of Pd/SnO_2_/G was significantly higher than that of Pd/G (~70% advantage) and SnO_2_/G (~30 times advantage) at room temperature. Both Pd/SnO_2_/rGO in [206] and Pd/SnO_2_/G in [197] have good sensitivity, but the sensing response of the Pd/SnO_2_/G-based hydrogen gas sensor is faster than the other. The possible reason for this is that graphene has better conductivity than reduced graphene oxide due to its functional groups and defects.

**Figure 46:**
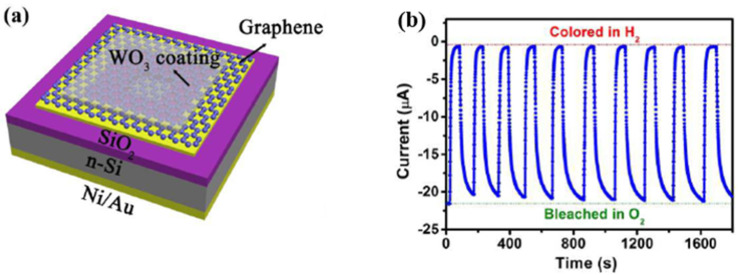
(**a**) Schematic of the optoelectronic Pd-WO_3_/Graphene sensor. (**b**) Cyclic performance of the device [209]. Copyright (2018), with permission from Elsevier.

Since its two-dimensionality, high optical transmittance and electrical conductivity, graphene sheets can also be used as an active layer for electrons/holes separation [212,213] and optoelectronic hydrogen sensing [209]. Chen et al. reported in [209] an optoelectronic hydrogen sensor based on a gasochromic Pd-loaded WO_3_ (Pd-WO_3_) film and a graphene/Si heterojunction structure, as shown in Figure 46a. However, when Pd-WO_3_ was exposed to a hydrogen gas test environment, it absorbed light through the intraband absorption due to the formation of H_x_WO_3_, reducing the light transmittance of the film [214]. When the sensor was exposed to hydrogen gas (4% H_2_/Ar mixed gas) and a 10 mW laser with a wavelength of 980 nm, it had rapid response and recovery time as reported. This optoelectronic hydrogen sensor has a low detection limit at room temperature, even down to 0.05 vol%. Furthermore, as shown in Figure 37b, it exhibits good repeatability, improving the sensor’s stability and lifetime.

**Figure 47:**
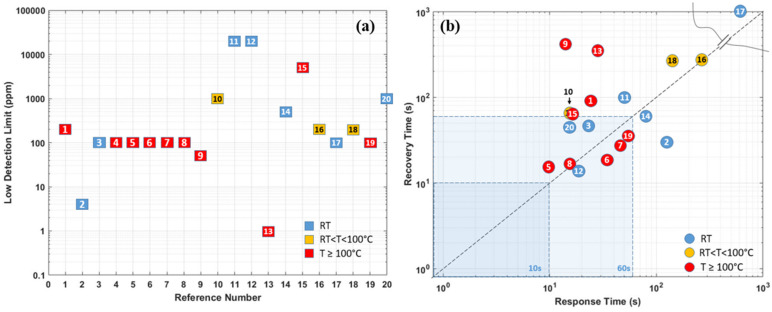
Regarding to Table 4, schematic illustrations of the comparison of the reported: (**a**) low detection limits. The x-axis “Reference Number” represents the 1st column“Ref. Nr.“ in Table 4. (**b**) Response time/recovery time under experimental conditions in literature.

Table 4 shows a list of comparisons of the sensing performance of several current MOS/graphene (e.g., GO, rGO)-based hydrogen gas sensors. The critical parameters, such as low detection limits, operating temperature, response/recovery time, are illustrated in Figure 47 schematically. Despite their high operating temperature, the power consumption is significantly lower than that of the MOS-based gas sensor because the maximal operating temperatures are mainly lower than 250 °C. Additionally, graphene’s unique physical and chemical properties (e.g., GO, rGO) improved the sensing response both in response/recovery time and selectivity.

## 5. Conclusions

Nanotechnology- and nanostructure-based hydrogen gas sensors were reviewed in this article, and in particular their unique sensing mechanisms, operating environments, and sensing responses.

With ongoing technological developments in nanotechnology and synthesis methods, several materials have been employed as the sensing materials, such as conventional approaches like transit metals, metal oxide semiconductors and their combinations, and new materials like graphene and its derivatives. Furthermore, innovative nanostructures and nanocomposites are investigated in many pieces of research to improve the sensing performance and stability due to high safety requirements in hydrogen energy-based facilities and vehicles avoiding hydrogen leakages and explosion hazards.

The conventional materials, like the nanostructured transit metals or MOS, have been synthesized and dispersed on the sensor surfaces, which on the one hand reduces the power consumption and increases the sensing response. On the other hand, however, it can lead to low conductivity, inhomogeneity of hydride reaction, and noise in the sensing signals. Moreover, the requirement of an external heater for MOS to maintain high operation temperatures also means high energy consumption and thermal safety problems. Thus, other material is required to form a highly conductive surface or pathways for electrons transition during the sensing process. Transition metal oxides such as RuO_2_, NiO, MnO_2_, Co_3_O_4_, and SnO_2_ are being studied continuously as candidate materials for gas sensing, and the metal oxide nanostructures such as SnO_2_, ZnO and Cu_2_O nanowires or nanorods with heterojunction structures show high sensitivity to various gases.

In comparison, graphene and its derivatives have the advantage of high sensitivity, low detection limit, and facile operation at low temperatures. Their unique structures and electronic properties also ensure high electron mobility and low electronic noise. With the help of CVD methods, the functional groups on their surfaces can be modified and engineered for sensing target gas molecules even at ppm/ppb levels. These advantages are generating a trend in the application of novel materials for nanoscale sensing performances. The application of graphene and its derivatives (e.g., GO, rGO) provides a new millstone in research towards nanoscale structures or clusters in gas sensing, achieving significant electrical conductivity and sensing selectivity through graphene functionalization. However, most research announcements fail to deliver adequate information on the chemical stability of graphene-based materials working under specific conditions required by chemical processes that occur in the sensing layer. Notably, there are still not many reliable graphene-based hydrogen leakage sensors available in the market that meet industry safety requirements.

Generally, nanomaterials-based gas sensors have obvious advantages:

(1) Very high surface-to-volume ratio. Large exposed surfaces of sensing materials ensure a high density of defects for possible improvement of electron transfer, such as vacancies or dangling bonds. As reported in many works, certain optimized particle sizes can raise the sensing performance of the nanomaterial-based gas sensor;
(2) The operating temperature of nanomaterials-based gas sensors is lower than traditional gas sensors based on bulk materials because of adsorption sites created by dangling bonds on the surface;
(3) The unsatisfied bonds on the surface of nanosized materials also enable the surface functionalization and activation towards the target gas in sensing applications, which allows precise control and surface engineering methodologies to enhance the sensing performance in a wide range.

In conclusion, nanotechnology- and nanocomposite-based materials enable an ideal platform for hydrogen gas sensing and other sensing applications. The remarkable advantages encourage innovative sensor designs focusing on increasing catalytic activity, adsorption/desorption parameters, reducing operating temperatures, rapid sensing response, and improving sensor performance. In-depth research and understanding of the influence of the size-effect of nanomaterials on physical and chemical properties modifications can help us develop more effective methodologies in surface activation and surface functionalization in order to improve the operation of gas sensors.

## Figures and Tables

**Table 1:** Comparison of sensing performances towards hydrogen gas of Pd-based gas sensors in literature. Unless specified, the sensor response is defined by the given Equation (1). (*) Sensor response given by the form R_a_/R_g_. (**) The recovery times were not given directly in their publications and were approximately read out from the sensing response curves. RT: room temperature, DC: direct current, RF: radio frequence, SAW: surface acoustic wave, PVD: physical vapour deposition.

Ref. Nr.	Sensor Materials	Operating Temperature	Synthesis Methods	Low Detection Limit	$\mathrm{Response}/\mathrm{Recovery}\;\mathrm{Time}\;(\mathrm{Response})\;\mathrm{at}\;H_{2}\%$	Literature
1	Pd film	RT	DC sputtering	100 ppm	25/10 s (17%) at 1.2%	[58]
2	Pd film/Pt heater	50 °C	---	200 ppm	30/17 s (0.2%) at 4000 ppm	[59]
3	Pd film/Pt heater	400 °C	---	200 ppm	10/15 s (4%) at 4000 ppm	[59]
4	Pd/SnSe film	RT	DC magnetron sputtering	0.91 ppb	73.1/23.7 s (3225 *) at 1000 ppm	[61]
5	$\mathrm{Pd}/{\mathrm{TiO}}_{2}$ film	RT	DC magnetron sputtering	0.4%	4/8 s (−) at 0.4%	[60]
6	Pd-Ni alloy thin-film	RT	RF magnetron co-sputtering, SAW	0.01%	7/30 s (2.75 kHz) at 0.1%,	[33]
7	Pd/ZnO film	RT	pulsed laser deposition	0.2%	12/16 s (−) at 2%	[39]
8	Pd/Y alloy film	RT	magnetron co-sputtering	0.1%	33/27 s (24.1 mV) at 1%	[32]
9	Pd/Co NWs	RT	electrodeposition	0.1%	200/500 s (~0.2%) at 1%	[64]
10	PA Pd NWs	RT	deposition	0.1%	70/140 s (~7%) at 1%	[66]
11	Pd-Si NWs	200–400 °C	PVD	0.01%	10/30 s (1.5 *) at 0.1%	[69]
12	Pd NTs	296–336 K	electrospun, deposition	314 ppm	12/18 s **(2.1%) at 1.8%	[72]
13	Pd/Cu NWs	RT	electrodeposition	7 ppm	4/4 s (1.5 kHz) at 1%	[73]
14	Pd/Si NWs	RT	DC magnetron sputtering	5 ppm	−/−	[74]
15	Pd NWs @ZIF-8_2h	RT	lithographically patterned nanowire, electrodeposition	1000 ppm	13/6 s (0.3%) at 0.1%	[75]
16	Pd NWs @ZIF-8_4h	RT	lithographically patterned nanowire, electrodeposition	600 ppm	30/8 s (0.7%) at 0.1%	[75]
17	$\mathrm{Pd}\;\mathrm{NPs}\;\mathrm{coated}\;{\mathrm{SnO}}_{2}$	160 °C	electrospun, magnet sputtering	0.25 ppm	4 s/− (30 *) at 100 ppm	[77]
18	$\mathrm{Pd}\;\mathrm{NPs}\;/{\;\mathrm{SiO}}_{2}$ /Si	RT	Deposition	2%	6/45 s (51.4%) at 2%	[78]
19	$\mathrm{Pd}\;\mathrm{NPs}\;/{\;\mathrm{SiO}}_{2}$ /Si	RT	Deposition	1%	1.4 s/14 s (88%) at 1%	[79]
20	$\mathrm{PMMA}/\mathrm{Pd}\;\mathrm{NPs}\;/{\;\mathrm{SiO}}_{2}$ /Si	RT	deposition, Spin coating	100 ppm	−/−	[82]
21	Pd NPs / ZnO	RT	RF magnetron sputtering	0.5 ppm	18.8 s/− (91.2%) at 0.1%	[86]
22	$\mathrm{Pd}\;\mathrm{NPs}/{\;\mathrm{SnO}}_{2}$	200 °C	solvothermal method	10 ppm	4/10 s (315.34 *) at 3000 ppm	[87]
23	$\mathrm{Pd}\;\mathrm{NPs}\;/{\;\mathrm{SnO}}_{2}$ NWs	300 °C	chemical Deposition	1 ppm	150/400 s ** (27.8 *) at 100 ppm	[88]
24	Pd/BN/ZnO NWs	200 °C	atomic layer deposition	0.5 ppm	160/90 s (7.95 *) at 10 ppm	[93]

**Table 2:** Comparison of sensing performances towards hydrogen gas of metal oxide semiconductor (MOS)-based gas sensors in the literature. Unless specified, the sensor response is defined by the given Equation (1). (*) Sensor response given by the form R_a_/R_g_. (**) The recovery times were not given directly in their publications and were approximately read out from the sensing response curves. RT: room temperature, DC: direct current.

Ref. Nr.	Sensor Materials	Operating Temperature	Synthesis Methods	Low Detection Limit	$\mathrm{Response}/\mathrm{Recovery}\;\mathrm{Time}\;(\mathrm{Response})\;\mathrm{at}\;H_{2}\%$	Literature
1	${\mathrm{SnO}}_{2}$ NW	150 °C	DC sputtering	10 ppm	−/− () at 1000 ppm	[105]
2	${\mathrm{SnO}}_{2}$ NPs	350 °C	thermal evaporation method	400 ppm	11/17 s ** (4.5 V) at 400 ppm	[106]
3	${\mathrm{SnO}}_{2}$ NFs	RT	hydrothermal method	100 ppm	63/500 s (80.20%) at 1000 ppm	[111]
4	$\mathrm{Pd}/{\;\mathrm{SnO}}_{2}$	200 °C	solvothermal method	10 ppm	4/10 s (315.34 *) at 3000 ppm	[87]
5	$\mathrm{Pd}/{\;\mathrm{SnO}}_{2}$ Film	175 °C	magnetron sputtering method	100 ppm	1/512 s (115.9 kHz) at 2000 ppm	[112]
6	$\mathrm{Pt}/{\;\mathrm{SnO}}_{2}$	RT	hydrothermal method, irradiated photochemical reduction method	100 ppm	0.33/29.60 s (88.35%) at 1000 ppm	[113]
7	$\mathrm{Pt}/{\;\mathrm{SnO}}_{2}$	350 °C	sol-gel and hydrothermal methods	0.08 ppm	29/36 s (~60 *) at 100 ppm	[114]
8	$\mathrm{Au}/{\;\mathrm{SnO}}_{2}$	200 °C	hydrothermal method	1 ppb	1/3 s (25 *) at 100 ppm	[115]
9	Pd/ZnO NFs	250 °C	hydrothermal method, UV reduction method	0.5 ppm	450/500 s (2.5 *) at 50 ppm	[122]
10	ZnO	400 °C	Wet chemical method	2000 ppm	5/7 s (10 *) at 2000 ppm	[107]
11	ZnO rods	350 °C	---	10 ppm	700/1100 s ** (−) at 100 ppm	[121]
12	Hollow ZnO particles	225 °C	hydrothermal	2 ppm	139 s/>30 min ** (89%) at 100 ppm	[125]
13	ZnO NFs	350 °C	electrospinning	0.1 ppm	400/200 s ** (74.7 *) at 100 ppb	[126]
14	$\mathrm{ZnO}/{\;\mathrm{SnO}}_{2}$ NFs	330 °C	electrospinning	25 ppm	69/175 s (10 *) at 100 ppm	[123]
15	$\mathrm{ZnO}/{\;\mathrm{SnO}}_{2}$ NFs	300 °C	electrospinning, Ar plasma	10 ppm	24/165 s (18 *) at 100 ppm	[123]
16	$\mathrm{ZnO}/0.1{\;\mathrm{SnO}}_{2}$ NFs	300 °C	electrospinning	50 ppb	−/− (50.1 *) at 50 ppb	[127]
17	TiO	RT	reactive DC magnetron sputtering	30 ppm	$(10^{4}\;$*) at 1000 ppm	[128]
18	Pt/TiO	RT	pressing, sintering	30 ppm	10/20 s (6000 *) at 1000 ppm	[129]
19	$\mathrm{Pt}/{\mathrm{WO}}_{3}$	RT	electrostatic spray deposition (ESD)	0.2%	15 s/10 min (18.5 *) at 1%	[131]
20	$\mathrm{Pt}/{\mathrm{WO}}_{3}$	RT	fermto second laser, hydrothermal method	0.02%	15/33 s (522 pm%(*v*/*v*)) at 1%	[136]
21	$\mathrm{Pd}/0.1{\mathrm{In}}_{2}O_{3}$/ZnO	350 °C	electrospinning, ultraviolet irradiation method	50 ppb	−/− (172 *) at 50 ppb	[141]

**Table 3:** Comparison of sensing performances towards hydrogen gas of the transition metal/graphene (e.g., GO, rGO)-based gas sensors in literature. Unless specified, the sensor response is defined by Equation (1). RT: room temperature, CVD: chemical vapour deposition, HTRJ: high-temperature reducing jet.

Ref. Nr.	Sensor Material	Operating Temperatur	Method	Low Detection Limit	$\mathrm{Response}/\mathrm{Recovery}\;\mathrm{Time}\;(\mathrm{Response})\;\mathrm{at}\;H_{2}\%$	Liter-ature
1	G	RT	CVD, e-beam evaporation	200 ppm	1/3 min (−) at 0.1%	[157]
2	GO	RT	Modified Hummer method and spray pyrolysis method	60 sccm	100/437.2 s (16.16%)	[159]
3	rGO	RT	Modified Hummers method	200 ppm	11/36 s (6%) at 200 ppm	[160]
4	Pd/G	RT	CVD	1%	3/9 min (5.88%) at 1%	[162]
5	Pd/rGO	RT	HTRJ process	25 ppm	73/126 s (14.8%) at 2%	[170]
6	Pd/G	RT	CVD, dip-coating process	200 ppm	18/300 s (−) at 0.5%	[163]
7	Pd/G	180 °C	Exfoliation, Soft lithography	10 ppm	15/16 s (−) at 100 ppm	[164]
8	Pd/G	254 °C	Deposition	1 ppm	16/14 s (−) at 1000 ppm	[165]
9	PMMA/Pd/G	RT	CVD	250 ppm	1.81/5.52 min (66.7%) at 2%	[85]
10	Pd/GO	RT	CVD	0.5%	6/7 s (−) at 1%	[181]
11	Pt/G	200 °C	Polymer-assisted hydrothermal (HT) method	10 ppm	9/10 s (−) at 1%	[182]
12	PEDOT:PSS/GO	RT	Modified Hummers’ method	30 ppm	30/25 s (4.2%) at 100 ppm	[183]
13	Pd/PANI/rGO	RT	Chemical reduction	100 ppm	20/50 s (25%) at 1%	[176]
14	Pt/G	RT	Hummers’ method	500 ppm	0.97/0.92 s (1.6%) at 1%	[184]
15	Pt/rGO	50 °C	Modified Hummers’ method	0.3%	63/104 s (−) at 0.5%	[185]
16	Pt/rGO	RT	Modified Hummers and Offman method	1 ppm	−/−	[186]
17	Pd/Ag/G	70–190 °C	Spin Coating, Sputter	100 ppm	56 s/− (15.23%) at 500 ppm	[187]
18	Pd/G	RT	CVD	0.1 ppm	12/15 s (−) at 100 ppm	[179]
19	Pd/LIG	RT	Laser-induced graphene (LIG)	600 ppm	6/20 min (−) at 1%	[180]

**Table 4:** Comparison of sensing performances towards hydrogen gas of metal oxide semiconductor (MOS)/graphene (e.g., GO, rGO)-based gas sensors in literature. Unless specified, the sensor response is defined by the given Equation (1). (*) Sensor response given by the form R_a_/R_g_. (**) The recovery times were not given directly in their publications and were read out approximately from the sensing response curves in the corresponding literature. RT: room temperature, CVD: chemical vapour deposition.

Ref. Nr.	Sensor Material	Operating Temperature	Method	Low Detection Limit	$\mathrm{Response}/\mathrm{Recovery}\;\mathrm{Time}\;(\mathrm{Response})\;\mathrm{at}\;H_{2}\%$	Literature
1	ZnO/G	150 °C	Hummer’s method	200 ppm	22/90 s (3.5 *) at 200 ppm	[188]
2	ZnO/GO	RT	simple wet-chemical coating technique	4 ppm	114/30 s (3.42 *) at 1000 ppm	[189]
3	ZnO/rGO	RT	Electrochemical exfoliation	100 ppm	21.04/47.09 s (484.1% *) at 100 ppm	[190]
4	ZnO/rGO	250 °C	Modified Hummers method [C23]	100 ppm	−/− (30%) at 500 ppm	[191]
5	ZnO/Ag/Pd/rGO	150 °C	Modified Hummers method	100 ppm	10/14 s (59%) at 100 ppm	[192]
6	ZnO/rGO	150 °C	Modified Hummers method	100 ppm	33/19 s (46%) at 100 ppm	[192]
7	Ag/ZnO/rGO	150 °C	Modified Hummers method	100 ppm	45/27 s (51%) at 100 ppm	[192]
8	ZrO2/ZnO/rGO	150 °C	Modified Hummers method	100 ppm	15/16 s (52%) at 100 ppm	[192]
9	Pt/ZnO/rGO	100 °C	Modified Hummers method	50 ppm	12/412 s (99%) at 400 ppm	[193]
10	SnO_2_/rGO	80 °C	Modified Hummers method	1000 ppm	15/61 s(1.58%) at 1000 ppm	[195]
11	Pd/SnO_2_/G	RT	CVD	2%	50/100 s (11%) at 2%	[197]
12	SnO_2_/G	RT	CVD	2%	18/12 s (0.35%) at 2%	[197]
13	Ni/ZnO/rGO	150 °C	Hummer‘s method	1 ppm	28/320 s (63.8%) at 100 ppm	[198]
14	CuO/rGO	RT	Thermal heating from GO at 180 °C	50 ppm	80/60 s (12%) at 1500 ppm	[202]
15	TiO_2_/G	125 °C	Hummers’ chemical method	5000 ppm	16/61 s (18%) at 5000 ppm	[204]
16	MoS_2_/rGO	60 °C	Modified Hummers method	200 ppm	261/260 s (15.6%) at 200 ppm	[205]
17	Pd/SnO_2_/rGO	RT	Modified Hummers method	100 ppm	600 s/>2000 s ** (55%) at 10,000 ppm	[206]
18	SnO_2_/rGO	60 °C	Modified Hummers method	200 ppm	119.6 s/265 s (19.6%) at 1000 ppm	[207]
19	Pd/WO_3_/rGO	100 °C	Modified Hummers method	100 ppm	52/35 s (150 *) at 500 ppm	[208]
20	Pd/WO_3_/G	RT	CVD-Method	0.1%	13/43 s(90%) at 4%	[208]

## References

[1] Adoption of the Paris Agreement, FCCC/CP/2015/L.9/Rev.1. UNFCCC Secretariat. https://unfccc.int/resource/docs/2015/cop21/eng/l09r01.pdf.

[2] Toyota Mirai 2019 Electronic Brochure. https://www.toyota.com/content/ebrochure/2019/mirai_ebrochure.pdf.

[3] Hübert T., Boon-Brett L., Black G., Banach U. (2011). Hydrogen sensors-a review. Sens. Actuators B Chem..

[4] Hübert T., Boon-Brett L., Buttner W.J. (2016). Hydrogen properties and technologies of its production and use. Sensors for Safety and Process Control in Hydrogen Technologies.

[5] Buttner W., Burgess R., Post M., Rivikin C. (2011). Summary and Findings from the NREL/DOE Hydrogen Sensor Workshop (8 June 2011).

[6] Hydrogen and Fuel Cell Technologies Office Multi-Year Research, Development, and Demonstration Plan. https://www.energy.gov/eere/fuelcells/downloads/hydrogen-and-fuel-cell-technologies-office-multi-year-research-development.

[7] Buttner W.J., Post M.B., Burgess R., Rivkin C. (2011). An overview of hydrogen safety sensors and requirements. Int. J. Hydrogen Energy.

[8] Penner R.M. (2017). A nose for hydrogen gas: Fast, sensitive H_2_ sensors using electrodeposited nanomaterials. Acc. Chem. Res..

[9] Yang F., Donavan K., Kung S.C., Penner R.M. (2012). The surface scattering-based detection of hydrogen in air using a platinum nanowire. Nano Lett..

[10] Deng Y.H., Hübert T., Boon-Brett L., Buttner W.J. (2019). Development of semiconducting metal oxide gas sensors. Semiconducting Metal Oxides for Gas Sensing.

[11] Fukai Y., Hull R., Parisi J., Osgood R.M., Warlimont H. (2005). Atomistic states of hydrogen in metals. The Metal-Hydrogen System: Basic Bulk Properties.

[12] Li J., Hu H.F., Yao C.B. (2018). Hydrogen sensing performance of silica microfiber elaborated with Pd nanoparticles. Mater. Lett..

[13] Dekura S., Kobayashi H., Kusada K., Kitagawa H. (2019). Hydrogen in palladium and storage properties of related nanomaterials: Size, shape, alloying, and metal-organic framework coating effects. ChemPhysChem.

[14] Nakatsuji H., Hada M. (1985). Interaction of a hydrogen molecule with palladium. J. Am. Chem. Soc..

[15] Bartczak W.M., Stawowska J. (2004). Interaction of dihydrogen with transition metal (Pd, Ni, Ag, Cu) clusters. Struct. Chem..

[16] Johnson N., Lam B., Macloed B.P., Sherbo R., Moreno-Gonzalez M., Fork D.K., Berlinguette C.P. (2019). Facets and vertices regulate hydrogen uptake and release in palladium nanocrystals. Nat. Mater..

[17] Nanba Y., Tsutsumi T., Ishimoto T., Koyama M. (2017). Theoretical study of the hydrogen absorption mechanism into a palladium nanocube coated with a metal-organic framework. J. Phys. Chem. C.

[18] Grammatikkopoulos P., Cassidy C., Singh V., Sowwan M. (2014). Coalescence-induced crystallisation wave in Pd nanoparticles. Sci. Rep..

[19] Halgren T.A., Lipscomb W.N. (1977). The synchronous-transit method for determining reaction pathways and locating molecular transition states. Chem. Phys. Lett..

[20] Flanagen T.B., Oates W.A. (1991). The palladium-hydrogen system. Annu. Rev. Mater. Sci..

[21] Brian D.A., Chen A.C. (2011). The role of palladium in hydrogen economy. Mater. Today.

[22] Abens P.C., Burgers W.G. (1962). Surface structure and electrochemical potential of palladium while adsorbing hydrogen in aqueous solution. Trans. Faraday Soc..

[23] Lewis F.A. (1982). The palladium-hydrogen system: A survey of hydride formation and effects of hydrogen contained within the metal lattices. Platin. Met. Rev..

[24] Frieske H., Wicke E. (1973). Magnetic susceptibility and equilibrium diagram of PdHn. Ber. Bunsenges. Für Phys. Chem..

[25] Akiba H., Kobayashi H., Kitagawa H., Kofu M., Yamamuro O. (2015). Glass transition and positional ordering of hydrogen in bulk and nanocrystalline palladium. Phys. Rev. B.

[26] Lässer R., Klatt K.H. (1983). Solubility of hydrogen isotopes in palladium. Phys. Rev. B.

[27] Gupta D., Dutta D., Kumar M., Barman P.B., Sarkar C.K., Hazra S.K. (2014). A low temperature hydrogen sensor based on palladium nanoparticles. Sens. Actuators B Chem..

[28] Lee E., Lee J.M., Koo J.H., Lee W.L., Lee T. (2010). Hysteresis behavior of electrical resistance in Pd thin films during the process of absorption and desorption of hydrogen gas. Int. J. Hydrogen Energy.

[29] Othonos A., Kalli K., Tsai D.P. (2000). Optically thin palladium films on silicon-based substrates and nanostructure formation: Effects of hydrogen. Appl. Surf. Sci..

[30] Dus R., Nowakowski R., Nowicka E. (2005). Chemical and structural components of work function changes in the process of palladium hydride formation with thin Pd film. J. Alloy. Compd..

[31] Ollagnier A., Fabre A. (2013). Activation process of reversible Pd thin film hydrogen sensors. Sens. Actuators B.

[32] Sharma B., Yadav H.M., Kim J.S. Multilayer thin film deposition of Pd/Ag alloy as an application for hydrogen. Proceedings of the 2017 IEEE Sensors Applications Symposium (SAS).

[33] Wang W., Liu X.L., Mei S.C., Liu M.W., Lu C., Lu M.H. (2019). Development of a high stability Pd-Ni alloy thin-film coated SAW device for sensing hydrogen. Sensors.

[34] Wadell C., Nugroho F.A.A., Lidström E., Landolo B., Wagner J.B., Langhammer C. (2015). Hysteresis-free nanoplasmonic Pd-Au alloy hydrogen sensors. Nano Lett..

[35] Yoshimura K., Nakano S., Uchinashi S., Yamaura S., Kimura H., Inoue A. (2007). A hydrogen sensor based on Mg-Pd alloy thin film. Meas. Sci. Technol..

[36] Zhao M., Wong M.H., Man H.C., Ong C.W. (2017). Resistive hydrogen sensing response of Pd-decorated ZnO nanosponge film. Sens. Actuators B Chem..

[37] Liu Q., Yao J.Y., Wang Y.P., Sun Y.N., Ding G.F. (2019). Temperature dependent response/recovery characteristics of Pd/Ni thin film based hydrogen sensor. Sens. Actuator B. Chem..

[38] Lee E., Lee J.M., Lee E., Noh J.S., Joe J.H., Jung B., Lee W. (2010). Hydrogen gas sensing performance of Pd-Ni alloy thin films. Thin Solid Film.

[39] Viespe C., Miu D. (2017). Surface acoustic wave sensor with Pd/ZnO bilayer structure for room temperature hydrogen detection. Sensors.

[40] Song H., Chen Y.P., Gang Z., Liu Y. (2015). Optical fiber hydrogen sensor based on an annealing-stimulated Pd-Y thin film. Sens. Actuators B Chem..

[41] Deng X.L., Zhou C.D., Li Z.L., Cheng K. (2014). Research about WO_3_/Pd film preparation for reflection type optical fiber hydrogen sensor. Appl. Mech. Mater..

[42] Yamauchi M., Ikeda R., Kitagawa H., Takata M. (2008). Nanosize effects on hydrogen storage in palladium. J. Phys. Chem. C.

[43] Bardhan R., Hedges L.O., Pint C.L., Javey A., Whitelam S., Urban J.J. (2013). Uncovering the intrinsic size dependence of hydriding phase transformation in nanocrystals. Nat. Mater..

[44] Sachs C., Pundt A., Kirchheim R., Winter M., Reetz M.T., Fritsch D. (2001). Solubility of hydrogen in single-sized palladium cluster. Phys. Rev. B.

[45] Stuhr U., Wipf H., Udovic T.J., Weissmuller J., Gleiter H. (1995). The vibrational excitations and the position of hydrogen in nanocrystalline palladium. J. Phys. Condens. Matter.

[46] Stuhr U., Wipf H., Andersen K.H., Hahn H. (2000). Neutron scattering study of the vibrational behaviour of H-doped nanocrystalline Pd. Phys. B Condens. Matter.

[47] Suleiman M., Jisrawi N.M., Dankert O., Reetz M.T., Bähtz C., Kirchheim R., Pundt A. (2003). Phase transition and lattice expansion during hydrogen loading of nanometer sized palladium clusters. J. Alloy. Compd..

[48] Eastman J., Thompson L.J., Kestel J. (1993). Narrowing of the palladium-hydrogen miscibility gap in nanocrystalline palladium. Phys. Rev. B.

[49] Baldi A., Narayan T.C., Koh A.L., Dionne J.A. (2014). In situ detection of hydrogen-induced phase transitions in individual palladium nanocrystals. Nat. Mater..

[50] Griessen R., Strohfeldt N., Giessen H. (2016). Thermodynamics of the hybrid interaction of hydrogen with palladium nanoparticles. Nat. Mater..

[51] Griessen R., Feenstra R. (1985). Volume changes during hydrogen absorption in metals. J. Phys. F Met. Phys..

[52] Narayan T.C., Hayee F., Baldi A., Koh A.L., Sinclair R., Dionne J.A. (2017). Direct visualization of hydrogen absorption dynamics in individual palladium nanoparticles. Nat. Commun..

[53] Alefeld G. (1972). Phase transitions of hydrogen in metals due to elastic interaction. Ber. Bunsenges. Für Phys. Chem..

[54] Wadell C., Pingel T., Olsson E., Zoric L., Zhdanov V.P., Langhammer C. (2014). Thermodynamics of hydrogen formation and decomposition in supported sub-10nm Pd nanoparticles of different sizes. Chem. Phys. Lett..

[55] Maxwell J.C. (1875). On the dynamical evidence of the molecular constitution of bodies. Nature.

[56] Schwarz R.B., Khachaturyan A.G. (1995). Thermodynamics of open two-phase systems with coherent interfaces. Phys. Rev. Lett..

[57] Schwarz R.B., Khachaturyan A.G. (2006). Thermodynamics of open two-phase systems with coherent interfaces: Application to metal-hydrogen systems. Acta Mater..

[58] Hu Y.M., Lei J.M., Wang Z., Yang S.L. (2016). Rapid response hydrogen sensor based on nanoporous Pd thin films. Int. J. Hydrogen Energy.

[59] Liu Q., Yao J.Y., Wu Y.J., Wang Y.P., Ding G.F. (2019). Two operating modes of palladium film hydrogen sensor based on suspended micro hotplate. Int. J. Hydrogen Energy.

[60] Mao S., Zhou H., Wu S.H., Yang J.J., Li Z.Y., Wei X.B., Wang X.R., Wang Z.Y., Li J. (2018). High performance hydrogen sensor based on Pd/TiO_2_ composite film. Int. J. Hydrogen Energy.

[61] Xu H.Y., Liu Y.J., Liu H., Dong S.C., Wu Y.P., Wang Z.G., Wang Y.M., Wu M.S., Han Z.D., Hao L.Z. (2021). Pd-decorated 2D SnSe ultrathin film on SiO_2_/Si for room-temperature hydrogen detection with ultrahigh response. J. Alloy. Compd..

[62] Pak Y., Lim N., Kumaresn Y., Lee R., Kim K., Kim T.H., Kim S.M., Ham M.H., Jung G.Y. (2015). Palladium nanoribbon array for fast hydrogen gas sensing with ultrahigh sensitivity. Adv. Mater..

[63] Hughes R.C., Schubert W.K., Zipperian T.E., Rodriguez J.L., Plut T.A. (1987). Thin-film palladium and silver alloys and layers for metal-insulator-semiconductor sensors. J. Appl. Phys..

[64] Du L.L., Feng D.L., Xing X.X., Fu Y., Fonsecs L.F., Yang D.C. (2019). Palladium/cobalt nanowires with improved hydrogen sensing stability at ultra-low temperatures. Nanoscale.

[65] Yang D., Valentin L., Carpena J., Otano W., Resto O., Fonseca L.F. (2012). Temperature-activated reverse sensing behavior of Pd nanowire hydrogen sensors. Small.

[66] Lee J.S., Seo M.H., Choi K.W., Yoo J.Y., Jo M.S., Yoon J.B. (2019). Stress-engineered palladium nanowires for wide range (0.1%–3.9%) of H_2_ detection with high durability. Nanoscale.

[67] Ahn J.H., Yun J., Moon D.I., Choi Y.K., Park I.K. (2015). Self-heated silicon nanowires for high performance hydrogen gas detection. Nanotechnology.

[68] Lupan O., Postica V., Hoppe M., Wolff N., Polonsky O., Pauporte T., Viana B., Majerus O., Kienle L., Faupel F. (2018). PdO/PdO_2_ functionalized ZnO : Pd films for lower operating temperature H_2_ gas sensing. Nanoscale.

[69] Yun J., Ahn J.H., Moon D.I., Choi Y.K., Park I.K. (2019). Joule-Heated and Suspended Silicon Nanowire Based Sensor for Low-Power and Stable Hydrogen Detection. ACS Appl. Mater. Interfaces.

[70] Offermans P., Tong H.D., van Riji C.J.M., Merken P., Brongersam S.H., Crego-Calama M. (2009). Ultralow-power hydrogen sensing with single palladium nanowires. Appl. Phys. Lett..

[71] Yang F., Taggart D.K., Penner R.M. (2010). Joule heating a palladium nanowire sensor for accelerated response and recovery to hydrogen gas. Small.

[72] Cho M., Zhu J.X., Kim H., Kang K., Park I.K. (2019). Half-pipe palladium nanotube-based hydrogen sensor using a suspended nanofiber scaffold. ACS Appl. Mater. Interfaces.

[73] Wang W., Liu X.L., Mei S.C., Jia Y.N., Liu M.W., Xue X.F., Yang D.C. (2019). Development of a Pd/Cu nanowires coated SAW hydrogen gas sensor with fast response and recovery. Sens. Actuators B Chem..

[74] Baek J., Jang B.J., Kim M.H., Kim W., Kim J., Rim H.J., Shin S., Lee T., Cho S., Lee W.Y. (2017). High-performance hydrogen sensing properties and sensing mechanism in Pd-coated p-type Si nanowire arrays. Sens. Actuators B Chem..

[75] Koo W., Qiao S.P., Ogata A.F., Jha G., Jang J.S., Chen V.T., Kim I.D., Penner R.M. (2017). Accelerating palladium nanowire H_2_ sensors using engineered nanofiltration. ACS Nano.

[76] Zheng L.J., Zheng S.Z., Wei H.R., Du L.L., Zhu Z.Y., Chen J., Yang D.C. (2019). Palladium/Bismuth/Copper hierarchical nano-architectures for efficient hydrogen evolution and stable hydrogen detection. ACS Appl. Mater. Interfaces.

[77] Wang F.P., Hu K.L., Liu H.C., Zhao Q., Wang K.Z., Zhang Y.X. (2020). Low temperature and fast response hydrogen gas sensor with Pd coated SnO_2_ nanofiber rods. Int. J. Hydrogen Energy.

[78] Kumar R., Malik S., Mehta B.R. (2015). Interface induced hydrogen sensing in Pd nanoparticle/graphene composite layers. Sens. Actuators B Chem..

[79] Behzadi Pour G., Fekri Aval L. (2017). Comparison of fast response and recovery Pd nanoparticles and Ni thin film hydrogen gas sensors based on metal-oxide-semiconductor structure. Nano.

[80] Bentarzi H. (2011). The MOS structure. Transport in Metal-Oxide-Semiconductor Structure: Mobile Ions Effects on the Oxide Properties.

[81] Kumar M.P., Logesh K., Dutta S., Sharma R.K., Rawal D.S. (2019). Limitations of Mott-Schottky analysis for organic metal-insulator-semiconductor capacitors. The Physics of Semiconductor Devices.

[82] Chen M.R., Mao P., Qin Y.Y., Wang J., Xie B., Wang X.Z., Han D.Y., Wang G.H., Song F.Q., Han M. (2017). Response characteristics of hydrogen sensors based on PMMA-membrane-coated palladium nanoparticle films. ACS Appl. Mater. Interfaces.

[83] Hulme J., Komaki M., Nishimura C., Gwak J. (2011). The effect of gas mixtures on hydrogen permeation through Pd-Ag/V-Ni alloy composite membrane. Curr. Appl. Phys..

[84] Miguel C.V., Mendes A., Tosti S., Madeira M. (2012). Effect of CO and CO_2_ on H_2_ permeation through finger-like Pd-Ag membranes. Int. J. Hydrogen Energy.

[85] Hong J., Lee S., Seo J., Pyo S., Kim J., Lee T. (2015). A highly sensitive hydrogen sensor with gas selectivity using a PMMA membrane-coated Pd nanoparticle/single-layer graphene hybrid. ACS Appl. Mater. Interfaces.

[86] Rashid T.R., Phan D.T., Chung G.S. (2014). Effect of Ga-modified layer on flexible hydrogen sensor using ZnO nanorods decorated by Pd catalysts. Sens. Actuators B.

[87] Li Y.X., Deng D.Y., Chen N., Xing X.X., Liu X., Xiao X.C., Wang Y.D. (2017). Pd nanoparticles composited SnO2 microspheres as sensing materials for gas sensors with enhanced hydrogen response performances. J. Alloy. Compd..

[88] Kim J.H., Mirzael A., Kim H.W., Kim S.S. (2019). Improving the hydrogen sensing properties of SnO_2_ nanowires-based conductometric sensors by Pd-decoration. Sens. Actuators B Chem..

[89] Annanouch F.E., Roso S., Haddi Z., Vallejos S., Blackmann C., Vilic T., Llobet E. (2016). p-Type PdO nanoparticles supported on n-type WO_3_ nanoneedles for hydrogen sensing. Thin Solid Films.

[90] Wang Z.H., Huang S.N., Men G.L., Han D.M., Gu F.B. (2018). Sensitization of Pd loading for remarkably enhanced hydrogen sensing performance of 3DOM WO_3_. Sens. Actuators B.

[91] Sanger A., Kumar A., Kumar A., Chandra R. (2016). Highly sensitive and selective hydrogen gas sensor using sputtered grown Pd decorated MnO_2_ nanowalls. Sens. Actuators B.

[92] Baek D.H., Kim J. (2017). MoS_2_ gas sensor functionalized by Pd for the detection of hydrogen. Sens. Actuators B Chem..

[93] Weber M., Kim J.Y., Lee J.H., Kim J.H., Iatsunskyi I., Coy E., Miele P., Bechelany M., Kim S.S. (2019). Highly efficient hydrogen sensors based on Pd nanoparticles supported on boron nitride coated ZnO nanowires. J. Mater. Chem. A.

[94] Gao L.J., Fu Q., Wei M.M., Zhu Y.F., Liu Q., Crumlin E., Liu Z., Bao X.H. (2016). Enhanced nickel catalyzed methanation confined under hexagonal boron nitride Shells. ACS Catal..

[95] Weber M., Lamboux C., Navarra B., Miele P., Zanna S., Dufond M.E., Santinacci L., Bechelany M. (2018). Boron nitride as a novel support for highly stable palladium nanocatalysts by atomic layer deposition. Nanomaterials.

[96] Ganose A.M., Scanlon D.O. (2016). Band gap and work function tailoring of SnO_2_ for improved transparent conducting ability in photovoltaics. J. Mater. Chem. C.

[97] Guo Y., Ma L., Mao K.K., Ju M.G., Bai Y.Z., Zhao J.J., Zeng X.C. (2019). Eighteen functional monolayer metal oxides: Wide bandgap semiconductors with superior oxidation resistance and ultrahigh carrier mobility. Nanoscale Horiz..

[98] Deng Y.H. (2019). Sensors for environmental gases. Semiconducting Metal Oxides for Gas Sensing.

[99] Nakate U.T., Lee G.H., Ahmad R., Patil P., Bhopate D.P., Hahn Y.B., Yu Y.T., Suh E.K. (2018). Hydrothermal synthesis of p-type nanocrystalline NiO nanoplates for high response and low concentration hydrogen gas sensor application. Ceram. Int..

[100] Zhang J., Qin Z.Y., Zeng D.W., Xie C.S. (2017). Metal-oxide-semiconductor based gas sensors: Screening, preparation, and integration. Phys. Chem. Chem. Phys..

[101] Barsan N., Weimar U. (2001). Conduction model of metal oxide gas sensors. J. Electroceram..

[102] Min B.K., Friend C.M. (2007). Heterogeneous gold-based catalysis for green chemistry: Low-temperature CO oxidation and propene oxidation. Chem. Rev..

[103] Kim H.J., Lee J.H. (2014). Highly sensitive and selective gas sensors using p-type oxide semiconductors: Overview. Sens. Actuators B Chem..

[104] Barsan N., Simion C., Heine T., Pokhrel S., Weimar U. (2010). Modeling of sensing and transduction for p-type semiconducting metal oxide based gas sensors. J. Electroceram..

[105] Shen Y.B., Wang W., Fan A.F., Wei D.Z., Liu W.G., Han C., Shen Y.S., Meng D., San X.G. (2015). Highly sensitive hydrogen sensors based on SnO_2_ nanomaterials with different morphologies. Int. J. Hydrogen Energy.

[106] Zhu L., Zeng W., Li Y.Q. (2019). A non-oxygen adsorption mechanism for hydrogen detection of nanostructured SnO_2_ based sensors. Mater. Res. Bull..

[107] Krishnakumar T., Kiruthiga A., Jozwiak E., Moulaee K., Neri G. (2020). Development of ZnO-based sensors for fuel cell cars equipped with ethanol steam-reformer for on-board hydrogen production. Ceram. Int..

[108] Hübert T., Boon-Brett L., Buttner W.J. (2018). Hydrogen Sensors. Sensors for Safety and Process Control in Hydrogen Technologies.

[109] Lavanya N., Sekar C., Fazio E., Neri F., Leonardi S.G., Neri G. (2017). Development of a selective hydrogen leak sensor based on chemically doped SnO_2_ for automotive applications. Int. J. Hydrogen Energy.

[110] Korotcenkov G., Cho B.K. (2012). Ozone measuring: What can limit application of SnO_2_-based conductometric gas sensors?. Sens. Actuators B Chem..

[111] Liu G., Wang Z., Chen Z.H., Yang S.L., Fu X.X., Huang R., Li X.K., Xiong J., Hu Y.M., Gu H.S. (2018). Remarkably enhanced room-temperature hydrogen sensing of SnO_2_ nanoflowers via vacuum annealing treatment. Sensors.

[112] Yang L., Yin C.B., Zhang Z.L., Zhou J.J., Xu H.H. (2017). The investigation of hydrogen gas sensing properties of SAW gas sensor based on palladium surface modified SnO_2_ thin film. Mater. Sci. Semicond. Process..

[113] Chen Z.H., Hu K.Y., Yang P.Y., Fu X.X., Wang Z., Yang S.L., Xiong J., Zhang X.H., Hu Y.M., Gu H.S. (2019). Hydrogen sensors based on Pt-decorated SnO_2_ nanorods with fast and sensitive room-temperature sensing performance. J. Alloy. Compd..

[114] Yin X.T., Zhou W.D., Li J., Wang Q., Wu F.Y., Dastan D., Wang D., Garmestani H., Wang X.M., Ţălu Ş. (2019). A highly sensitivity and selectivity Pt-SnO_2_ nanoparticles for sensing applications at extremely low level hydrogen gas detection. J. Alloy. Compd..

[115] Wang Y., Zhao Z.T., Sun Y.J., Li P.W., Ji J.L., Chen Y., Zhang W.D., Hu J. (2017). Fabrication and gas sensing properties of Au-loaded SnO_2_ composite nanoparticles for highly sensitive hydrogen detection. Sens. Actuators B Chem..

[116] Abdulah Q.N., Yam F.K., Hassan Z., Bououdina M. (2014). Hydrogen gas sensing performance of GaN nanowires-based sensor at low operating temperature. Sens. Actuators B Chem..

[117] Ma S.Y., Hu M., Zeng P., Li M.D., Yan W.J., Qin Y.X. (2014). Synthesis and low-temperature gas sensing properties of tungsten oxide nanowires/porous silicon composite. Sens. Actuators B Chem..

[118] Gurlo A. (2011). Nanosensors: Towards morphological control of gas sensing activity. SnO_2_, In_2_O_3_, ZnO and WO_3_ case studies. Nanoscale.

[119] Yu H., Yang T.Y., Wang Z.Y., Li Z.F., Xiao B.X., Zhao Q., Zhang M.Z. (2017). Facile synthesis cedar-like SnO_2_ hierarchical micro-nanostructures with improved formaldehyde gas sensing characteristics. J. Alloy. Compd..

[120] Yang W.H., Lu W.C., Xue X.Y., Zang Q.J. (2015). A theoretical study on CO sensing mechanism of In-doped SnO2 (1 1 0) surface. Comput. Theor. Chem..

[121] Vallejos S., Gràcia I., Lednický T., Vojkuvka L., Figueras E., Hubálek J., Cané C. (2018). Highly hydrogen sensitive micromachined sensors based on aerosol-assisted chemical vapor deposited ZnO rods. Sens. Actuators B Chem..

[122] Kim J.H., Mirzaei A., Osada M., Kim H.W., Kim S.S. (2021). Hydrogen sensing characteristics of Pd-decorated ultrathin ZnO nanosheets. Sens. Actuators B Chem..

[123] Hu K.L., Wang F.P., Shen Z.J., Liu H.C., Zeng W., Wang Y. (2020). Ar plasma treatment on ZnO–SnO_2_ heterojunction nanofibers and its enhancement mechanism of hydrogen gas sensing. Ceram. Int..

[124] Jang S., Jung S., Baik K.H. (2020). Hydrogen sensing performance of ZnO Schottky diodes in humid ambient conditions with PMMA membrane layer. Sensors.

[125] Nakate U.T., Ahmad R., Patil P., Bhat K.S., Wang Y.S., Mahmoudi T., Yu Y.T., Suh E.K., Hahn Y.B. (2019). High response and low concentration hydrogen gas sensing properties using hollow ZnO particles transformed from polystyrene@ ZnO core-shell structures. Int. J. Hydrogen Energy.

[126] Kim J.H., Mirzaei A., Kim H.W., Kim S.S. (2019). Combination of Pd loading and electron beam irradiation for superior hydrogen sensing of electrospun ZnO nanofibers. Sens. Actuators B Chem..

[127] Lee J.H., Kim J.Y., Kim J.H., Kim S.S. (2019). Enhanced hydrogen detection in ppb-level by electrospun SnO_2_-loaded ZnO nanofibers. Sensors.

[128] Krško O., Plecenik T., Roch T., Grančič B., Satrapinskyy L., Truchlý M., Ďurina P., Gregor M., Kúš P., Plecenik A. (2017). Flexible highly sensitive hydrogen gas sensor based on a TiO_2_ thin film on polyimide foil. Sens. Actuators B Chem..

[129] Chen W.P., Xiong Y., Li Y.S., Cui P., Guo S.S., Chen W., Tang Z.L., Yan Z.J., Zhang Z.Y. (2016). Extraordinary room-temperature hydrogen sensing capabilities of porous bulk Pt–TiO_2_ nanocomposite ceramics. Int. J. Hydrogen Energy.

[130] Herrmann J.M., Pichat P. (1982). Metal-support interactions: An in situ electrical conductivity study of Pt/TiO_2_ catalysts. J. Catal..

[131] Lee J., Koo H., Kim S.Y., Kim S.J., Lee W. (2021). Electrostatic spray deposition of chemochromic WO_3_-Pd sensor for hydrogen leakage detection at room temperature. Sens. Actuators B Chem..

[132] Watanabe T., Okazaki S., Nakagawa H., Murata K., Fukuda K. (2010). A fiber-optic hydrogen gas sensor with low propagation loss. Sens. Actuators B Chem..

[133] Boudiba A., Roussel P., Zhang C., Olivier M.G., Snyders R., Debliquy M. (2013). Sensing mechanism of hydrogen sensors based on palladium-loaded tungsten oxide (Pd–WO_3_). Sens. Actuators B Chem..

[134] Liu B., Cai D.P., Liu Y., Wang D.D., Wang L.L., Wang Y.R., Li H., Li Q.H., Wang T.H. (2014). Improved room-temperature hydrogen sensing performance of directly formed Pd/WO3 nanocomposite. Sens. Actuators B Chem..

[135] Saenger M.F., Höing T., Robertson B.W., Billa R.B., Hofmann T., Schubert E., Schubert M. (2008). Polaron and phonon properties in proton intercalated amorphous tungsten oxide thin films. Phys. Rev. B.

[136] Zhou X., Dai Y.T., Karanja J.M., Liu F.F., Yang M.H. (2017). Microstructured FBG hydrogen sensor based on pt-loaded WO_3_. Opt. Express.

[137] Caucheteur C., Debliquy M., Lahem D., Mégret P. (2008). Catalytic fiber Bragg grating sensor for hydrogen leak detection in air. IEEE Photon. Technol. Lett..

[138] Geim A.K., Novoselov K.S., Rodgers P. (2010). The rise of graphene. Nanoscience and Technology: A Collection of Reviews from Nature Journals.

[139] Magne S., Rougeault S., Vilela M., Ferdinand P. (1997). State-of-strain evaluation with fiber Bragg grating rosettes: Application to discrimination between strain and temperature effects in fiber sensors. Appl. Opt..

[140] Katoch A., Kim J.H., Kwon Y.J., Kim H.W., Kim S.S. (2015). Bifunctional sensing mechanism of SnO2–ZnO composite nanofibers for drastically enhancing the sensing behavior in H2 gas. ACS Appl. Mater. Interfaces.

[141] Lee J.H., Kim J.H., Kim J.Y., Mirzaei A., Kim H.W., Kim S.S. (2019). ppb-Level selective hydrogen gas detection of Pd-functionalized In_2_O_3_-loaded ZnO nanofiber gas sensors. Sensors.

[142] Novoselov K.S., Geim A.K., Morozov S.V., Jiang D.E., Zhang Y., Dubonos S.V., Grigorieva I.V., Firsov A.A. (2004). Electric field effect in atomically thin carbon films. Science.

[143] Novoselov K.S., Geim A.K., Morozov S.V., Jiang D., Katsnelson M.I., Grigorieva I.V., Dubonos S.V., Firsov A.A. (2005). Two-dimensional gas of massless Dirac fermions in graphene. Nature.

[144] Emtsev K.V., Bostwick A., Horn K., Jobst J., Kellogg G.L., Ley L., McChesney J.L., Ohta T., Reshanov S.A., Röhrl J. (2009). Towards wafer-size graphene layers by atmospheric pressure graphitization of silicon carbide. Nat. Mater..

[145] Dan Y.P., Lu Y., Kybert N.J., Luo Z.T., Johnson A.T.C. (2009). Intrinsic response of graphene vapor sensors. Nano Lett..

[146] Morozov S.V., Novoselov K.S., Katsnelson M.I., Schedin F., Elias D.C., Jaszczak J.A., Geim A.K. (2008). Giant intrinsic carrier mobilities in graphene and its bilayer. Phys. Rev. Lett..

[147] Wu Z.S., Ren W.C., Wen L., Gao L.B., Zhao J.P., Chen Z.P., Zhou G.M., Li F., Cheng H.M. (2010). Graphene anchored with Co_3_O_4_ nanoparticles as anode of lithium ion batteries with enhanced reversible capacity and cyclic performance. ACS Nano.

[148] Ma L.P., Wu Z.S., Li J., Wu E.D., Ren W.C., Cheng H.M. (2009). Hydrogen adsorption behavior of graphene above critical temperature. Int. J. Hydrogen Energy.

[149] Sehrawat P., Islam S.S., Mishra P., Ahmad S. (2018). Reduced graphene oxide (rGO) based wideband optical sensor and the role of temperature, defect states and quantum efficiency. Sci. Rep..

[150] Shahriary L., Athawale A.A. (2014). Graphene oxide synthesized by using modified hummers approach. Int. J. Energy Environ. Eng..

[151] Dreyer D.R., Park S.J., Bielawski C.W., Ruoff R.S. (2010). The chemistry of graphene oxide. Chem. Soc. Rev..

[152] Wang X., Li X., Zhao Y., Chen Y., Yu J., Wang J. (2016). The influence of oxygen functional groups on gas-sensing properties of reduced graphene oxide (rGO) at room temperature. RSC Adv..

[153] Choi Y.R., Yoon Y.G., Choi K.S., Kang J.H., Shim Y.S., Kim Y.H., Jang H.W. (2015). Role of oxygen functional groups in graphene oxide for reversible room-temperature NO_2_ sensing. Carbon.

[154] Tian H., Yang Y., Xie D., Cui Y.L., Mi W.T., Zhang Y., Ren T.L. (2014). Wafer-scale integration of graphene-based electronic, optoelectronic and electroacoustic devices. Sci. Rep..

[155] Mattson E.C., Johns J.E., Pande K., Bosch R.A., Cui S., Gajdardziska-Josifovska M., Weinert M., Chen J.H., Hersam M.C., Hirschmugl C.J. (2014). Vibrational excitations and low-energy electronic structure of epoxide-decorated graphene. J. Phys. Chem. Lett..

[156] Liang H. (2014). Mid-infrared response of reduced graphene oxide and its high-temperature coefficient of resistance. AIP Adv..

[157] Shao Z.C., Hayasaka T., Liu H.L., Liang J.M., Wu Y.C., Lin L.W. Crumpled and stretchable graphene gas sensor with enhanced sensitivity to hydrogen. Proceedings of the 2019 IEEE 32nd International Conference on Micro Electro Mechanical Systems (MEMS).

[158] Celebi K., Buchheim J., Wyss R.M., Droudian A., Gasser P., Shorubalko I., Kye J., Lee C., Park H.G. (2014). Ultimate permeation across atomically thin porous graphene. Science.

[159] Shaban M., Ali S., Rabia M. (2019). Design and application of nanoporous graphene oxide film for CO_2_, H_2_, and C_2_H_2_ gases sensing. J. Mater. Res. Technol..

[160] Wang J.W., Singh B., Maeng S., Joh H.I., Kim G.H. (2013). Assembly of thermally reduced graphene oxide nanostructures by alternating current dielectrophoresis as hydrogen-gas sensors. Appl. Phys. Lett..

[161] Johnson J.L., Behnam A., Pearton S.J., Ural A. (2010). Hydrogen sensing using Pd-functionalized multi-layer graphene nanoribbon networks. Adv. Mater..

[162] Tang X.H., Haddad P.A., Mager N., Geng X., Reckinger N., Hermans S., Debliquy M., Paskin J.P. (2019). Chemically deposited palladium nanoparticles on graphene for hydrogen sensor applications. Sci. Rep..

[163] Ma J., Zhou Y.L., Bai X., Chen K., Guan B.O. (2019). High-sensitivity and fast-response fiber-tip Fabry–Pérot hydrogen sensor with suspended palladium-decorated graphene. Nanoscale.

[164] Yokoyama T., Tanaka T., Shimokawa Y., Yamachi R., Saito Y., Uchida K. (2018). Pd-functionalized, suspended graphene nanosheet for fast, low-energy multimolecular sensors. ACS Appl. Nano Mater..

[165] Sharma B., Kim J.S. (2018). MEMS based highly sensitive dual FET gas sensor using graphene decorated Pd-Ag alloy nanoparticles for H_2_ detection. Sci. Rep..

[166] Zhang M., Brooks L.L., Chartuprayoon N., Bosze W., Choa Y.H., Myung N.V. (2014). Palladium/single-walled carbon nanotube back-to-back Schottky contact-based hydrogen sensors and their sensing mechanism. ACS Appl. Mater. Interfaces.

[167] Yu Y.J., Zhao Y.R., Ryu S., Brus L.E., Kim K.S., Kim P. (2009). Tuning the graphene work function by electric field effect. Nano Lett..

[168] Haddad P.A., Flandre D., Raskin J.P. (2018). Intrinsic rectification in common-gated graphene field-effect transistors. Nano Energy.

[169] Sun Y.G., Tao Z.L., Chen J., Herricks T., Xia Y.N. (2004). Ag nanowires coated with Ag/Pd alloy sheaths and their use as substrates for reversible absorption and desorption of hydrogen. J. Am. Chem. Soc..

[170] Mohammadi M.M., Kumar A., Liu J., Liu Y., Thundat T., Swihart M.T. (2020). Hydrogen sensing at room temperature using flame-synthesized palladium-decorated crumpled reduced graphene oxide nanocomposites. ACS Sens..

[171] Liewhiran C., Tamaekong N., Wisitsoraat A., Tuantranont A., Phanichphant S. (2013). Ultra-sensitive H_2_ sensors based on flame-spray-made Pd-loaded SnO_2_ sensing films. Sens. Actuators B Chem..

[172] Nazarian-Samani M., Kim H.K., Park S.H., Youn H.C., Mhamane D., Lee S.W., Kim M.S., Jeong J.H., Haghighat-Shishavan S., Roh K.C. (2016). Three-dimensional graphene-based spheres and crumpled balls: Micro-and nano-structures, synthesis strategies, properties and applications. RSC Adv..

[173] Xia Y., Li R., Chen R.S., Wang J., Xiang L. (2018). 3D architectured graphene/metal oxide hybrids for gas sensors: A review. Sensors.

[174] Srivastava S., Kumar S., Singh V.N., Singh M., Vijay Y.K. (2011). Synthesis and characterization of TiO2 doped polyaniline composites for hydrogen gas sensing. Int. J. Hydrogen Energy.

[175] Nasirian S., Moghaddam H.M. (2014). Hydrogen gas sensing based on polyaniline/anatase titania nanocomposite. Int. J. Hydrogen Energy.

[176] Zou Y.J., Wang Q.Y., Xiang C.L., Tang C.Y., Chu H.L., Qiu S.J., Yan E.H., Xu F., Sun L.X. (2016). Doping composite of polyaniline and reduced graphene oxide with palladium nanoparticles for room-temperature hydrogen-gas sensing. Int. J. Hydrogen Energy.

[177] Sekar P., Anothumakkool B., Kurungot S. (2015). 3D polyaniline porous layer anchored pillared graphene sheets: Enhanced interface joined with high conductivity for better charge storage applications. ACS Appl. Mater. Interfaces.

[178] Kumar R., Varandani D., Mehta B.R., Singh V.N., Wen Z.H., Feng X.L., Müllen K. (2011). Fast response and recovery of hydrogen sensing in Pd–Pt nanoparticle–graphene composite layers. Nanotechnology.

[179] Shin D.H., Lee J.S., Jun J., An J.H., Kim S.G., Cho K.H., Jang J. (2015). Flower-like palladium nanoclusters decorated graphene electrodes for ultrasensitive and flexible hydrogen gas sensing. Sci. Rep..

[180] Zhu J.X., Cho M., Li Y.T., Cho I., Suh J.H., Orbe D.D., Jeong Y., Ren L.T., Park I. (2019). Biomimetic turbinate-like artificial nose for hydrogen detection based on 3D porous laser-induced graphene. ACS Appl. Mater. Interfaces.

[181] Li D.S., Le X.H., Pang J.T., Xie J. A SAW hydrogen sensor based on the composite of graphene oxide and palladium nanoparticles. Proceedings of the IEEE 32nd International Conference on Micro Electro Mechanical Systems (MEMS).

[182] Phan D.T., Youn J.S., Jeon K.J. (2019). High-sensitivity and fast-response hydrogen sensor for safety application using Pt nanoparticle-decorated 3D graphene. Renew. Energy.

[183] Zheng Y., Lee D., Koo H.Y., Maeng S. (2015). Chemically modified graphene/PEDOT: PSS nanocomposite films for hydrogen gas sensing. Carbon.

[184] Harley-Trochimczyk A., Chang J.Y., Zhou Q., Dong J., Pham T., Worsley M.A., Maboudian R., Zettl A., Mickelson W. (2015). Catalytic hydrogen sensing using microheated platinum nanoparticle-loaded graphene aerogel. Sens. Actuators B Chem..

[185] Lu X.J., Song X.J., Gu C.P., Ren H.B., Sun Y.F., Huang J.R. (2018). Freeze drying-assisted synthesis of Pt@ reduced graphene oxide nanocomposites as excellent hydrogen sensor. J. Phys. Chem. Solids.

[186] Lee J.S., Oh J., Jun J., Jang J. (2015). Wireless hydrogen smart sensor based on Pt/graphene-immobilized radio-frequency identification tag. ACS Nano.

[187] Sharma B., Kim J.S. (2018). Graphene decorated Pd-Ag nanoparticles for H_2_ sensing. Int. J. Hydrogen Energy.

[188] Anand K., Singh O., Singh M.P., Kaur J., Singh R.C. (2014). Hydrogen sensor based on graphene/ZnO nanocomposite. Sens. Actuators B Chem..

[189] Rasch F., Postica V., Schütt F., Mishra Y.K., Nia A.S., Lohe M.R., Feng X.L., Adelung R., Lupan O. (2020). Highly selective and ultra-low power consumption metal oxide based hydrogen gas sensor employing graphene oxide as molecular sieve. Sens. Actuators B Chem..

[190] Das S., Roy S., Bhattacharya T.S., Sarkar C.K. (2020). Efficient room temperature hydrogen gas sensor using ZnO nanoparticles-reduced graphene oxide nanohybrid. IEEE Sens. J..

[191] Galstyan V., Comini E., Kholmanov I., Faglia G., Sberveglieri G. (2016). Reduced graphene oxide/ZnO nanocomposite for application in chemical gas sensors. RSC Adv..

[192] Pal P., Yadav A., Chauhan P.S., Parida P.K., Gupta A. (2021). Reduced graphene oxide based hybrid functionalized films for hydrogen detection: Theoretical and experimental studies. Sens. Int..

[193] Drmosh Q.A., Yamani Z.H., Hendi A.H., Gondal M.A., Moqbel R.A., Saleh T.A., Khan M.Y. (2019). A novel approach to fabricating a ternary rGO/ZnO/Pt system for high-performance hydrogen sensor at low operating temperatures. Appl. Surf. Sci..

[194] Huang J.R., Wang L.Y., Shi C.C., Dai Y.J., Gu C.P., Liu J.H. (2014). Selective detection of picric acid using functionalized reduced graphene oxide sensor device. Sens. Actuators B Chem..

[195] Ren H.B., Gu C.P., Joo S.W., Cui J.Y., Sun Y.F., Huang J.R. (2018). Preparation of SnO_2_ nanorods on reduced graphene oxide and sensing properties of as-grown nanocomposites towards hydrogen at low working temperature. Mater. Express.

[196] Kamal T. (2017). High performance NiO decorated graphene as a potential H_2_ gas sensor. J. Alloy. Compd..

[197] Dhall S., Kumar M., Bhatnagar M., Mehta B.R. (2018). Dual gas sensing properties of graphene-Pd/SnO_2_ composites for H_2_ and ethanol: Role of nanoparticles-graphene interface. Int. J. Hydrogen Energy.

[198] Bhati V.S., Ranwa S., Rajamani S., Kumari K., Raliya R., Biswas P., Kumar M. (2018). Improved sensitivity with low limit of detection of a hydrogen gas sensor based on rGO-loaded Ni-doped ZnO nanostructures. ACS Appl. Mater. Interfaces.

[199] Zhang M., Zhen Y.H., Sun F.H., Xu C. (2016). Hydrothermally synthesized SnO_2_-graphene composites for H_2_ sensing at low operating temperature. Mater. Sci. Eng. B.

[200] Van Quang V., Van Dung N., Sy Trong N., Duc Hoa N., Van Duy N., Van Hieu N. (2014). Outstanding gas-sensing performance of graphene/SnO_2_ nanowire Schottky junctions. Appl. Phys. Lett..

[201] Lee J.H., Katoch A., Choi S.W., Kim J.H., Kim H.W., Kim S.S. (2015). Extraordinary improvement of gas-sensing performances in SnO2 nanofibers due to creation of local p–n heterojunctions by loading reduced graphene oxide nanosheets. ACS Appl. Mater. Interfaces.

[202] Zhang D.Z., Yin N.L., Jiang C.X., Xia B.K. (2017). Characterization of CuO–reduced graphene oxide sandwiched nanostructure and its hydrogen sensing characteristics. J. Mater. Sci. Mater. Electron..

[203] Zhang J.T., Liu J.F., Peng Q., Wang X., Li Y.D. (2006). Nearly monodisperse Cu_2_O and CuO nanospheres: Preparation and applications for sensitive gas sensors. Chem. Mater..

[204] Dutta D., Hazra S.K., Das J., Sarkar C.K., Basu S. (2015). Studies on p-TiO_2_/n-graphene heterojunction for hydrogen detection. Sens. Actuators B Chem..

[205] Venkatesan A., Rathi S., Lee I.Y., Park J., Lim D., Kang M., Joh H.I., Kim G.H., Kannan E.S. (2017). Molybdenum disulfide nanoparticles decorated reduced graphene oxide: Highly sensitive and selective hydrogen sensor. Nanotechnology.

[206] Peng Y.T., Zheng L.L., Zou K., Li C. (2017). Enhancing performances of a resistivity-type hydrogen sensor based on Pd/SnO_2_/RGO nanocomposites. Nanotechnology.

[207] Venkatesan A., Rathi S., Lee I.Y., Park J., Lim D., Kim G.H., Kannan E.S. (2016). Low temperature hydrogen sensing using reduced graphene oxide and tin oxide nanoflowers based hybrid structure. Semicond. Sci. Technol..

[208] Esfandiar A., Irajizad A., Akhavan O., Ghasemi S., Gholami M.R. (2014). Pd–WO_3_/reduced graphene oxide hierarchical nanostructures as efficient hydrogen gas sensors. Int. J. Hydrogen Energy.

[209] Chen M.Y., Zou L.P., Zhang Z.H., Shen J., Li D., Zong Q.J., Gao G.H., Wu G.M., Shen J., Zhang Z.X. (2018). Tandem gasochromic-Pd-WO_3_/graphene/Si device for room-temperature high-performance optoelectronic hydrogen sensors. Carbon.

[210] Bhati V.S., Ranwa S., Fanetti M., Valant M., Kumar M. (2018). Efficient hydrogen sensor based on Ni-doped ZnO nanostructures by RF sputtering. Sens. Actuators B Chem..

[211] Ayesh A.I., Alyafei A.A., Anjum R.S., Mohamed R.M., Abuharb M.B., Salah B., El-Muraikhi M. (2019). Production of sensitive gas sensors using CuO/SnO_2_ nanoparticles. Appl. Phys. A.

[212] Li X.M., Zhu H.W., Wang K.L., Cao A.Y., Wei J.Q., Li C.Y., Li Z., Li X., Wu D.H. (2010). Graphene-on-silicon Schottky junction solar cells. Adv. Mater..

[213] Zhang Q.C., Wang X.J., Li D., Zhang Z.X. (2014). Direct growth of nanographene on silicon with thin oxide layer for high-performance nanographene-oxide-silicon diodes. Adv. Funct. Mater..

[214] Feng W., Wu G.M., Gao G.H. (2014). Ordered mesoporous WO_3_ film with outstanding gasochromic properties. J. Mater. Chem. A.

